# Experimental Lineage and Computational Analysis of a General Aminoxyl-Based Oxidation Catalyst: Generality from Substrate-Specific Interactions

**DOI:** 10.1021/acscatal.5c05893

**Published:** 2025-10-07

**Authors:** Soren D. Rozema, Nicolò Tampellini, Jonas Rein, Matthew S. Sigman, Song Lin, Scott J. Miller

**Affiliations:** 1 Department of Chemistry, 5755 Yale University, New Haven, Connecticut 06520, United States; 2 Department of Chemistry and Chemical Biology, 5922 Cornell University, Ithaca, New York 14853, United States; 3 Department of Chemistry, 7060 University of Utah, Salt Lake City, Utah 84112, United States

**Keywords:** substrate generality, catalyst optimization, asymmetric catalysis, computational modeling, DFT

## Abstract

Traditionally, catalyst optimization in asymmetric catalysis is approached as an iterative, heuristic-based process, where point modifications of a hit catalyst are tested against one (or a small number of) model substrate(s). While optimization to high levels of selectivity is sometimes successful, catalyst generality with respect to substrate scope (i.e., its ability to deliver a diverse set of target products with consistently high levels of selectivity) is more elusive. This work describes and models computationally a successful peptide catalyst optimization campaign carried out on a verifiably diverse set of substrates, which delivered a highly selective and general chiral catalyst. The success of every generational improvement of the catalyst design is now rationalized with atomistic resolution by *ab initio* modeling of the individual substrate that mostly benefits from that generational improvement. Structural and topological insights about the noncovalent interaction networks orchestrating both the catalyst conformation and the substrate–catalyst interactions were dissected, culminating on the underpinnings of the optimized catalyst’s generality. Surprisingly and significantly, the generality of high selectivity for many substrates was found to be consistent with alternative, and indeed substrate-specific, interactions, suggesting that functional generality need not be a result of mechanistic homology.

## Graphical abstract

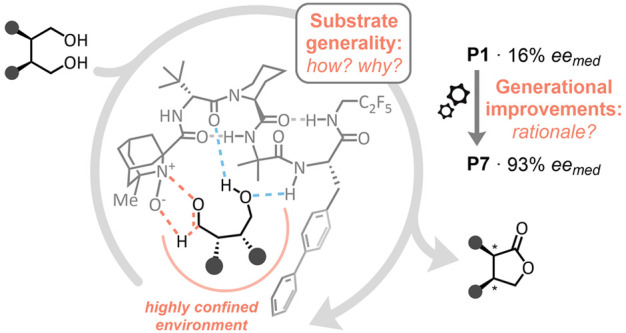

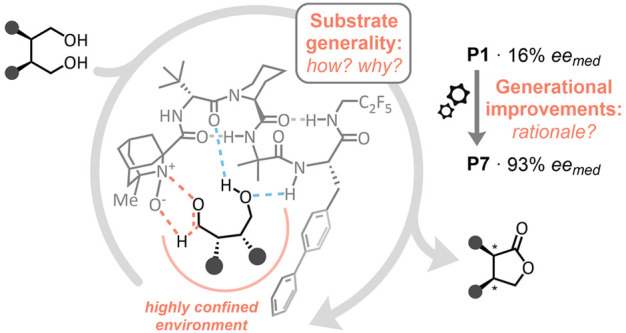

## Introduction

Asymmetric catalysis is now a central activity in the heart of organic synthesis.^1^ Catalyst optimization, however, remains a bottleneck due to selectivity or generality challenges, especially as substrate diversity increases.^2^ Optimization toward highly differentiated diastereomeric transition states, as required for high selectivity, can often conflict with structural features of diverse substrates.^3^ Accordingly, the pursuit of generality for chiral catalysts has been a daunting challenge,^2^ but momentum in the field is growing as generality-based catalyst optimization approaches are now emerging.^2,4–13^

We recently reported a peptide-based catalyst that achieves high enantioselectivities in an oxoammonium-mediated desymmetrization of diols.^8^ The best catalyst was found to be pentameric structure **P7** (Figure 1a) that possesses many of the desirable features of peptide-based catalysts.^14,15^ Among these, the capacity for multivalent noncovalent interactions and conformational flexibility, as a matter of speculation, was thought to be potentially operative *a priori*. Accordingly, our screening campaign began with well-known conformationally biased β-turn peptides,^16^ applied previously in many catalytic reactions across a variety of mechanistic manifolds.^14^ Yet, the optimization campaign rapidly diverged to unprecedented catalyst structures. Described herein are (a) the iterative decision process that resulted in catalyst evolution, not discussed in our previous manuscript,^8^ and (b) an expansive retrospective computational study that illuminates possible mechanistic underpinnings of the catalyst’s generality. This analysis resulted in the conclusion that the observed substrate generality involved substrate-specific interactions, which are not homologous across the scope—a highly unexpected outcome.

**Figure 1:**
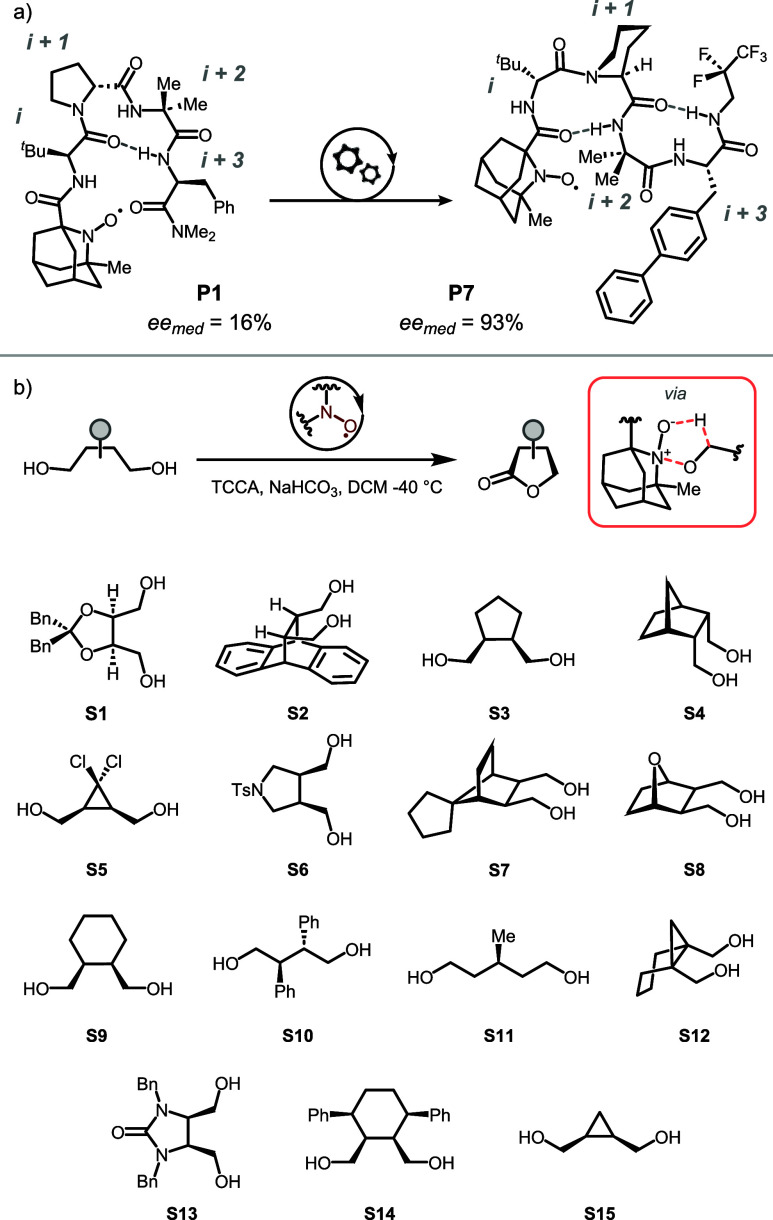
(a) Catalyst evolution: from the hit (**P1**) to the most general catalyst (**P7**). (b) Optimization conditions and substrate set for the *meso*-diol desymmetrization.

## Results and Discussion

### Catalyst Optimization Campaign

Catalyst hit identification within the Miller group often relies on the use of prior optimized peptide scaffolds and their adaptation to new systems. Due to both their historical reliability and their tendency for preorganization, β-turn biased peptides are often surveyed first. In the study of *meso*-diol desymmetrization, a new aminoxyl-containing catalytic residue, **Azc**, was utilized (3-methyl-2-azaadamantane carboxylate *N*-oxyl). Screening against dibenzyl ketal **S1**, a peptide with the **Azc** residue appended to the *N*-terminal “*n*”-position^17^ (**P1**, Table 1) demonstrated high selectivity (75% *ee*) and was selected for further optimization efforts. Three key features were identified for a series of single point modifications to answer significant questions for each element. First, the optimal diastereomeric configuration of the catalyst was examined; the four amino acids comprising peptide **P1** were in the *LDXL* configuration, where *X* denotes an achiral residue. Second, the β-turn biasing elements at *n* + 2 and *n* + 3 were modulated. Finally, a subsequent evaluation of both esters and amides was surveyed at the peptide *C*-terminus.

**Table 1:**
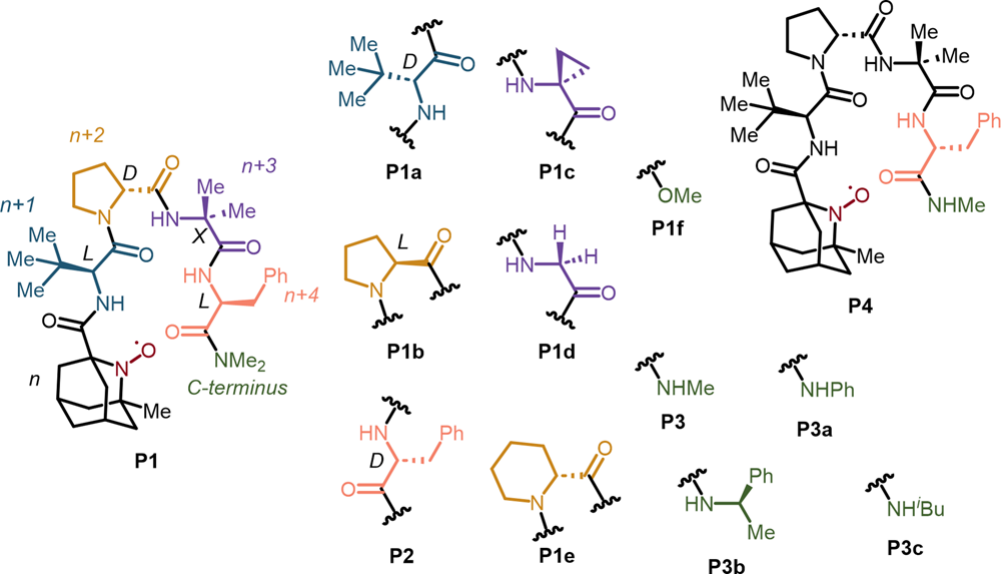

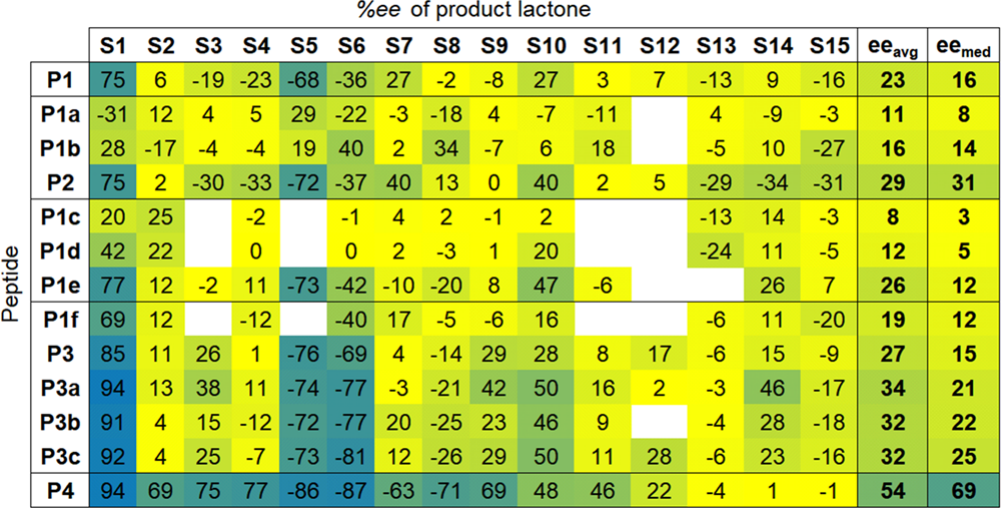
Initial Rounds of Catalyst Optimization to Catalyst **P4**

Optimization against a single substrate failed to provide a catalyst that was general across a range of diverse substrates, although some promising results were obtained (**P1**/**S1**, 75% *ee*, Table 1). Thus, a multisubstrate screening approach was undertaken, wherein each new catalyst prepared was screened against all (or most) of a set of 15 algorithmically selected diverse prochiral diols. The aim of this approach was to optimize toward general catalyst–substrate interactions as opposed to the specific interactions that may be unearthed using a single optimization substrate. This strategy led to larger amounts of data to further inform catalyst optimization.

The *LDXD* diastereomeric configuration of catalyst **P2** proved to be slightly more selective than the initial **P1** hit (Table 1), with small but noticeable increases in selectivity across many substrates (**S3**, **S4**, **S7**, **S10**, **S13**, **S14**, and **S15**). The other two diastereomeric configurations (**P1a** and **P1b**) failed to provide high levels of selectivity across the selection of diols, although it must be said that several individual pairings do indeed show increased selectivity (both **P1a** and **P1b** show increased selectivity for **S8**). Individual results such as these highlight the advantages of a generality-based optimization approach in circumventing local, narrow selectivity maxima in favor of optimizing toward higher, more global ones.

When the β-turn biasing elements (DPro-Aib) were analyzed, it was quickly noted that substitution of Aib for the cyclopropane-bearing Acpc (**P1c**) or Gly (**P 1d**) failed to provide enhanced selectivity. Curiously, however, substitution of ^D^Pro for ^D^Pip (**P1e**) showed selectivities comparable to or better than **P1**. At the time, this observation failed to elicit deeper thinking into the conformational changes this homologation would impart. Both Pro and Pip are well-known for inducing turn motifs in small peptides; however, their propensity to nucleate one specific secondary structure over another is markedly different. Proline has been shown, in particular with an adjacent disubstituted residue linked to the *C*-terminus, to nucleate β-turns with Pro at the *i* + 1 position.^18^ In contrast, Pip demonstrates a strong tendency to nucleate β-turns with itself at the *i* + 2 position. The fact that Pip behaved comparably in this system should have immediately alerted us that the conformation of the peptide in some bound state may not lie as the previously depicted β-turn but perhaps one where Pro and Pip occupy the *i* + 2 position relative to the β-turn.

Finally, analysis of the *C*-terminus caused rather significant selectivity perturbations for what we considered to be a minor change in the linear sequence. Modulation of the *N*,*N*-dimethyl *C*-terminus to a methyl ester (**P 1f**; Table 1) slightly diminished the median selectivity. Conversely, modification to the *C*-terminal *N*-methyl secondary amide (**P3**) led to significant enhancements in enantioselectivity for individual substrates (**S1**, **S5**, and **S6**). Although the median *ee* did not improve significantly, the individual increases led us to believe that the *C*-terminus would have an unexpectedly central role to play in peptide optimization. Encouragingly, we also observed similar increases utilizing *C*-terminal *N*-phenyl (**P3a**), *N*-α-methyl benzyl (**P3b**), and *N*-isobutyl (**P3c**) caps, with these peptides demonstrating modest increases in selectivity for a broader range of substrates as well (**S8**, **S9**, **S10**, and **S14**). Although we found enhanced selectivity for *C*-termini beyond methyl, analogue synthesis proved to be a challenge for many peptides, requiring alternative synthetic routes.

With a breadth of information on the catalyst behavior, the next phase of catalyst optimization was selection of a new starting point for single-point modifications. The selectivity-unlocking nature of **P3** was encouraging; if the contacts leading to high selectivity for **S1**, **S5**, and **S6** could be generalized to more substrates, this campaign would prove to be highly successful. Subsequently, we determined that the *C*-terminal secondary amide would be used in conjunction with the changes that resulted in both **P1e** and **P2**. Retaining the *C*-terminal secondary amide in combination with swapping ^D^Pro for ^D^Pip did not lead to substantial enhancements in individual selectivities but provided quantifiable increases for nearly every substrate examined (**P1e**). These incremental improvements, however, paled in comparison to altering the configuration of the sequence from *LDXL* to *LDXD*, providing **P4** from **P3**. Dramatic increases in selectivity were observed for nearly every substrate analyzed, with an increase in median *ee* from 31 to 69%.

### Further Optimization: More Specialized Residues

While crystallization of catalyst **P3** proved unattainable, we were able to obtain a single-crystal X-ray structure of analog **P3a**. This catalyst adopted a 3_10_-helix conformation in the solid state, which also features the expected β-turn motif nucleated by Pro-Aib. It was then hypothesized that this conformation would be retained in peptide **P4**, with only an inversion of stereochemistry at *n* + 4 present relative to **P3**. At the time, we hypothesized that this 3_10_-helix might be the active conformation of the substrate-bound peptide and would be responsible for the high levels of selectivity. Modifications for catalyst optimization were then selected on the basis of their potential to (1) stabilize the 3_10_-helix conformation or (2) destabilize the β-hairpin conformation. Two parameters were then prioritized to be tuned in the next round of optimization starting from **P4**: the backbone Pro-Aib segment and the *C*-terminal cap. It must be noted that the absolute stereochemistry of the catalysts was inverted at this point due to increased availability of ^“L”^ amino acids. The configuration of the *n* + 1 residue remained fixed as ^D^ *tert*-leucine (^D^Tle).

The *n* + 3 position was not surveyed at this stage due to diminished selectivities when replacing Aib for other disubstituted residues in the initial optimization (*vide supra*). However, the *n* + 2 residue was analyzed with mixed success. The substituted analogue α-methyl Pro (**P4a**) which has shown success in several other campaigns, dramatically reduced selectivity in substrates that showed improvement with **P4** (Table 2). Utilizing the previously beneficial Pip (**P5**) provided a noticeable boost in enantioselectivity overall with several substrates being particularly responsive to this change (**S4**, **S7**, and **S10**). Substitution for the benzannulated tetrahydroisoquinoline (Tic, **P4b**) also showed improved selectivity over **P4** (Pro); however, it was less advantageous than **P5** (Pip), which was chosen as the replacement for Pro going forward.

**Table 2:**
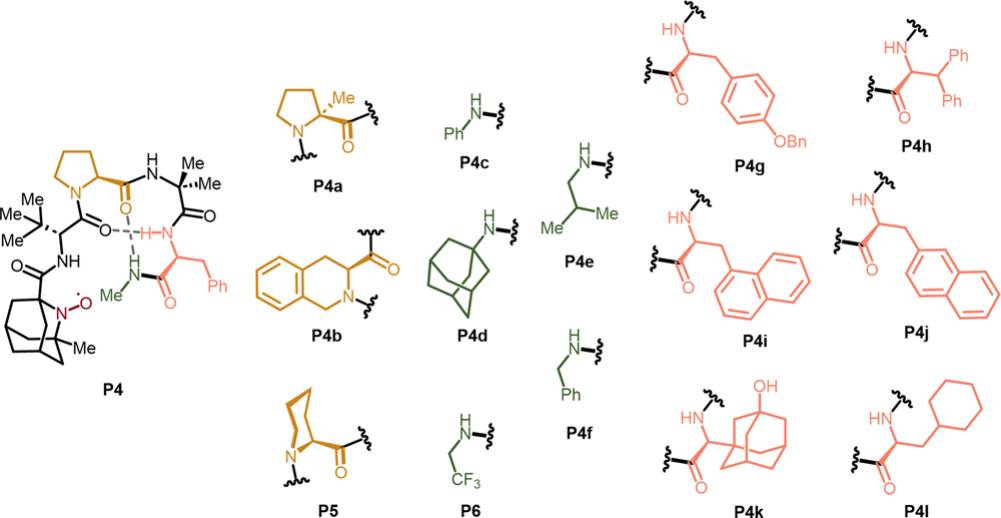

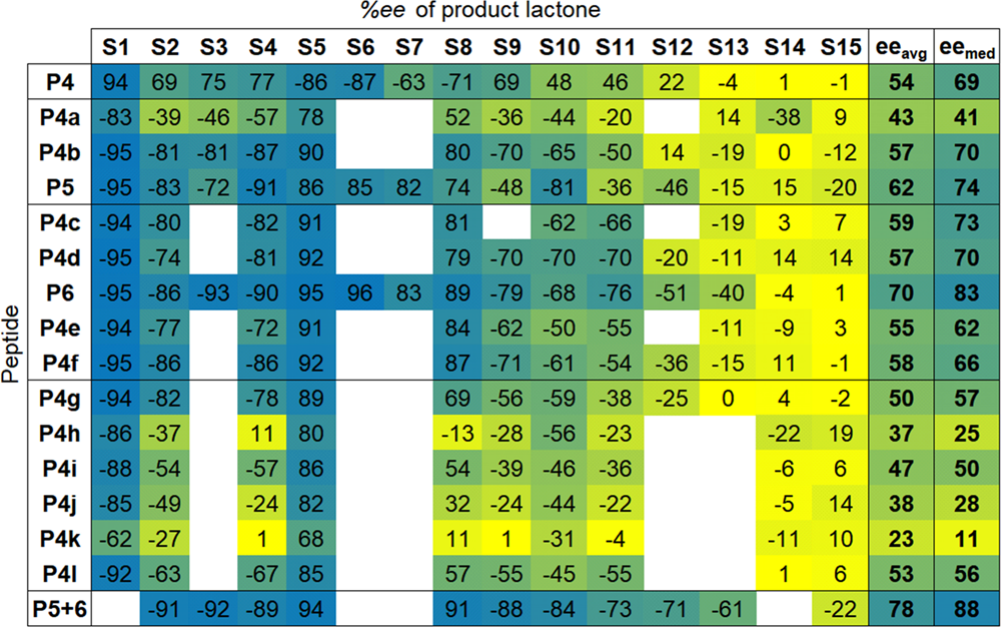
Optimization of Catalyst **P4** toward **P5 + 6**

Attention then turned to tuning the *C*-terminus. We hypothesized that increasing the acidity of the NH group would lead to increased selectivity, as we had seen in the earlier round of optimization (**P3a** compared to **P3**; Table 1). An enhanced hydrogen bond donor could potentially reinforce a rigidified catalyst conformation. It was also noticed that a slightly increased steric profile seemed to enhance the selectivity (**P3b** compared to **P3**; Table 1); therefore, several peptides with larger, more electron-deficient *C*-terminal secondary amide caps were prepared.

Two aniline-capped peptides were successfully prepared, with a simple *N*-phenyl cap (**P4c**) providing good boosts in enantioselectivity for several previously recalcitrant substrates (**S10** and **S11**). Increasing steric bulk at the cap also provided an increase in selectivity, as an *N*-adamantyl capped peptide (**P 4d**) provided similarly enhanced selectivities for **S10** and **S11**. Most notably, the combination of both a more electron-deficient and slightly larger cap (*N*-trifluoroethyl capped **P6**) caused the greatest improvement of the series, providing an increase in selectivity for every single substrate examined. Largely, we attributed the increase in selectivity to an increased electron-withdrawing character as opposed to a significant steric contribution. *C*-terminal caps with steric profiles similar to those of **P6** but with simple aliphatic (**P4e**) or aromatic (**P 4f**) substituents failed to provide substantive increases in selectivity even over **P4.**

Finally, the *n* + 4 residue was analyzed in relation to **P4**; an array of residues resembling phenylalanine were assayed. Direct analogs to Phe were analyzed, with *O*-benzyl tyrosine (**P4g**) providing a mix of increased and decreased selectivities and diphenylalanine (**P4h**), 1-naphthylalanine (**P4i**), and 2-naphthylalanine (**P4j**) diminishing selectivity in almost every case. Large aliphatic substituents similarly provided no increase in selectivity: 3-hydroxyadamantyl glycine (**P4k**) and cyclohexylalanine (**P4l**) substituted peptides diminished selectivity for all but one peptide–substrate combination.

### Final Optimization: Fine-Tuning

With two major advances for selectivity in hand, the substitutions leading from **P4** to both **P5** (Pro to Pip) and **P6** (methyl to trifluoroethyl C-terminus) were combined to provide an intermediate peptide **P5 + 6** that would be fine-tuned to provide the final catalyst. **P5 + 6** expectedly provides excellent selectivities across a diverse substrate set (Table 2), and only minor structural modifications were considered further. Namely, two positions were considered at this stage: the identity of the polyfluorinated *C*-terminal cap and the residue at the *n* + 4 position. First, pentafluoropropyl (**P5 + 6a**), heptafluorobutyl (**P5 + 6b**), as well as the sterically encumbered (1-trifluoromethyl)­cyclopropyl (**P5 + 6c**) amines were appended to the *C*-terminus; however, only minor changes were observed (Table 3). Peptide **P5 + 6a** showed a slight increase in performance against substrate **S10**, and therefore, the pentafluoropropyl tail was selected as the most advantageous C-terminus. Finally, tuning the *n* + 4 residue was attempted once again. Previous attempts at optimizing this position led to highly selective catalysts only when a phenylalanine or a derivative thereof (Nal, Tyr, etc.) was used. Unfortunately, substitution with homophenylalanine **P5 + 6d**, *O*-benyzl tyrosine **P5 + 6e**, or diphenylalanine **P5 + 6f** failed to provide any improvement over that of **P5 + 6**. In a final attempt to increase the selectivity, a single derivative of **P5 + 6a** was prepared with biphenylalanine at *n* + 4. This peptide, **P7**, provided slight enhancements across all *all* substrates tested and was selected as the final, fully optimized catalyst. **P7** provided high selectivities for substrates beyond the screening set, as even prochiral 1,3- and 1,5- diols were efficiently desymmetrized. This concluded the optimization campaign and led to our publication of enantioselective *meso*-diol oxidation.

**Table 3:**
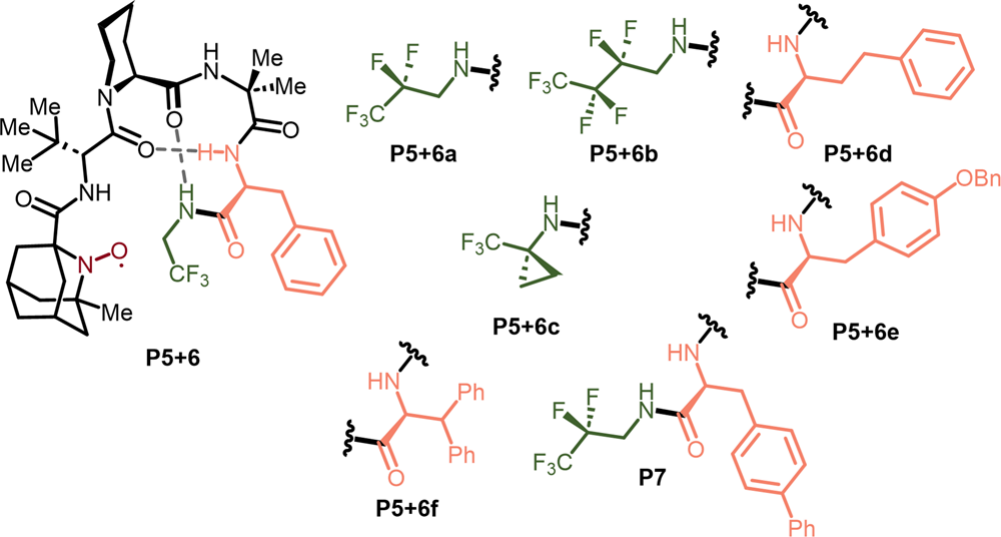

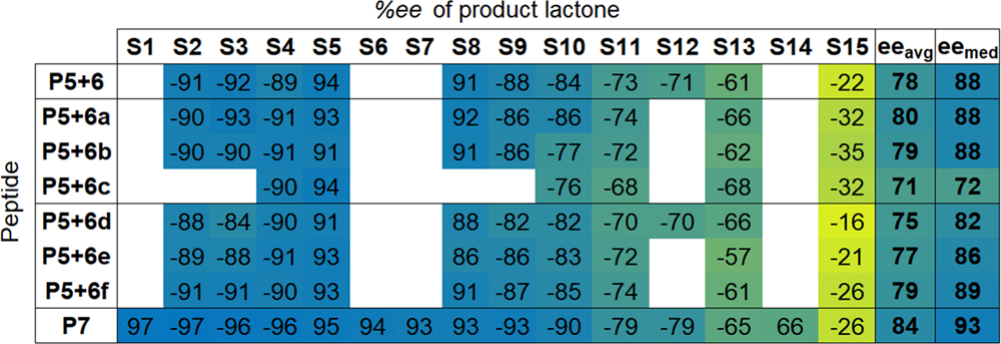
Final Rounds of Optimization

Throughout the later stages of optimization, obtaining conformational information via single-crystal X-ray analysis became a feature sought largely to confirm the hypothesis of a 3_10_ helix motif being responsible for selectivity. To our surprise, upon obtaining single crystal X-ray analysis of **P7**, we observed a double β-turn motif, with the most prominent turn nucleated with the catalytic residue (*n*) at the *i* position. Computational analysis of peptides **P1**–**P7** revealed an unexpected prominence of this β-turn in earlier generations of the catalyst, prompting a fuller computational investigation as to what conformational changes and alterations to interaction networks were elicited by residue-by-residue changes.

## Computational Methods

Computational modeling of enantioselective catalysis is known to be a complicated enterprise for different factors, including catalyst flexibility, additivity of numerous weak interactions, and of course the computational cost of performing numerous calculations at a high level of theory.^19–24^ Nevertheless, very significant progress has been made in the cost-to-accuracy ratio in recent years, with accurate geometrical and energetic descriptions of relatively small organic molecules coming at a computational cost that allows larger-scale modeling efforts to be carried out with reasonable time and resources.^22^ We present here a self-consistent and rational basis for the success of the catalyst optimization described above, encompassing the ground state free energy characterization of ∼6000 different structures across the optimization campaign.

The computational workflow was designed to rationalize what stereoelectronic effects enabled the development of the highly general catalyst for *meso*-diols desymmetrization. Our goal was to understand what role each generational modification of the catalyst played toward reaching the final structure (**P7**), ideally with atomic-level resolution of the interaction mode landscape. In this workflow, pairs of peptide catalysts were analyzed for a single substrate. A substrate was chosen for each peptide pair such that the generational advancement of the catalyst would induce the greatest improvement in selectivity for that substrate (as measured by enantiomeric excess), with a few substrate choices also affected by computational cost considerations. Once we defined the pairs, peptide–substrate covalent adducts were modeled as four possible stereoisomers, as alcohol binding to the oxoammonium moiety generates two new chiral elements: the first arising from the choice of which of the two *meso* alcohol groups is bound to the oxoammonium moiety and the second from which oxoammonium face was used for coordination.

Modeling of these covalent adducts consisted of a multilevel computational workflow to characterize their conformational landscape (see the Supporting Information for detailed computational procedures). In short, an initial metadynamics-based, unrestricted conformational search via CREST^25^ was followed by layers of geometrical and similarity refinement via XTB,^26^ FIRECODE,^27^ and ORCA,^28,29^ up to the ωB97M-V^30^/def2-TVZPP^31^/SMD^32^­(CH_2_Cl_2_)//R^2^SCAN-3c^33^/CPCM^34^­(CH_2_Cl_2_) level, from which free energy data were composed.

At this composite level, the DFT equilibrium geometries within 3 kcal/mol from the most stable (*G*_233 K_) for each oxoammonium alkoxide complex were compared to their stereoisomeric ensembles as well as the ensembles of the same substrate with the adjacent generation of the catalyst. These interaction modes were then used to qualitatively trace the origin of the success of each new generation of catalysts through ground-state conformational analysis. Mindful of the Curtin–Hammett principle,^35^ the choice to use ground states as a proxy for transition state conformational landscapes was due to the large scale of the modeling effort and the desire to avoid the computation of Hessian matrices for thousands of structures. The use of this heuristic is also justified by the very small geometrical difference between the oxoammonium alkoxide complexes and the following Cope elimination transition states, which causes the relative conformational energetics to be dominated by the backbone hydrogen bond networks of the catalyst and substrate. Moreover, electroanalytical independent rate measurements show that the Δ*G* ^‡^_obs_ for both diol **S9** and cyclohexylmethanol (as a proxy mono-ol) is very similar, while the one-pot competition experiment between the two substrates shows a selectivity ratio of 26.8 (Figure S6). This shows a mechanistic scenario where the energetic differentiation of the transition state structure reflects that of the oxoammonium alkoxide ground-state complexes (Δ*G*° ∼ ΔΔ*G* ^‡^_obs_) and offers experimental support for the modeling approach using ground states.

To further validate the use of ground states as a proxy for transition states, full transition state modeling of a minimal system (Azc-NHMe-1,4-butanediol adduct) was carried out at the same level of theory and provided the insight that the transition states for the Cope-type elimination^36–38^ (Figure 1b) are geometrically very similar to the relative ground states (<1 Å RMSD, root-mean-squared deviation of atomic positions) and that energetic trends for ground-state conformers closely reflect transition state ones, allowing a qualitative analysis of activation topologies based on ground-state interaction modes. The same modeling also revealed that, for the model system, all kinetically relevant transition states feature activation modes where the second alcohol interacts with another Lewis basic moiety, i.e., either the bound alcohol oxygen atom or the carbonyl oxygen of the Azc residue. This observation also supports the rationale of a *two-point interaction mode* of the substrate as having an influence on rate acceleration, as we forwarded in our previous work. This is also in agreement with the observation that two-point interaction ground states are dominant in the modeling of pentameric peptide catalysts.

### **P1** to **P3** and **P2** to **P4**: NMe_2_ to NHMe

The initial hit catalyst **P1** was obtained via a short screen of peptides and showed inconsistent performance across the diol test set. For this reason, we expected a complex conformational space featuring a multitude of topologically distinct activation modes. Indeed, the final ensemble for interactions of **S6** (desymmetrized in only 36% *ee*) featured many accessible states (six within 2 kcal/mol for the major enantiomer), and interestingly, the most stable ones all feature a secondary structure where Pro is *not* at the *i* + 1 position of a β-turn (as expected at the time of its synthesis) but at the *i* + 2, with ^D^Tle occupying *i* + 1 (Figure 2a–c). This structural feature will be maintained across all of the catalysts throughout the optimization campaign. Modifying the terminal NMe_2_ group to NHMe gave catalyst **P3**, which was able to desymmetrize substrate **S6** in 69% *ee* (+0.44 kcal/mol improvement in ΔΔ*G* ^‡^_obs_). Tracing back this effect to the most stable interaction modes for these two catalysts, it became evident that the additional hydrogen bond present for the NHMe end-cap imparted a significant improvement in the catalyst preorganization. Inspection of the most stable interaction modes showed how this change disfavors an activation mode for the minor enantiomer, where the second alcohol moiety coordinates to the top face of the catalyst rather than the bottom, which will ultimately become the dominant one in the following generations.

**Figure 2:**
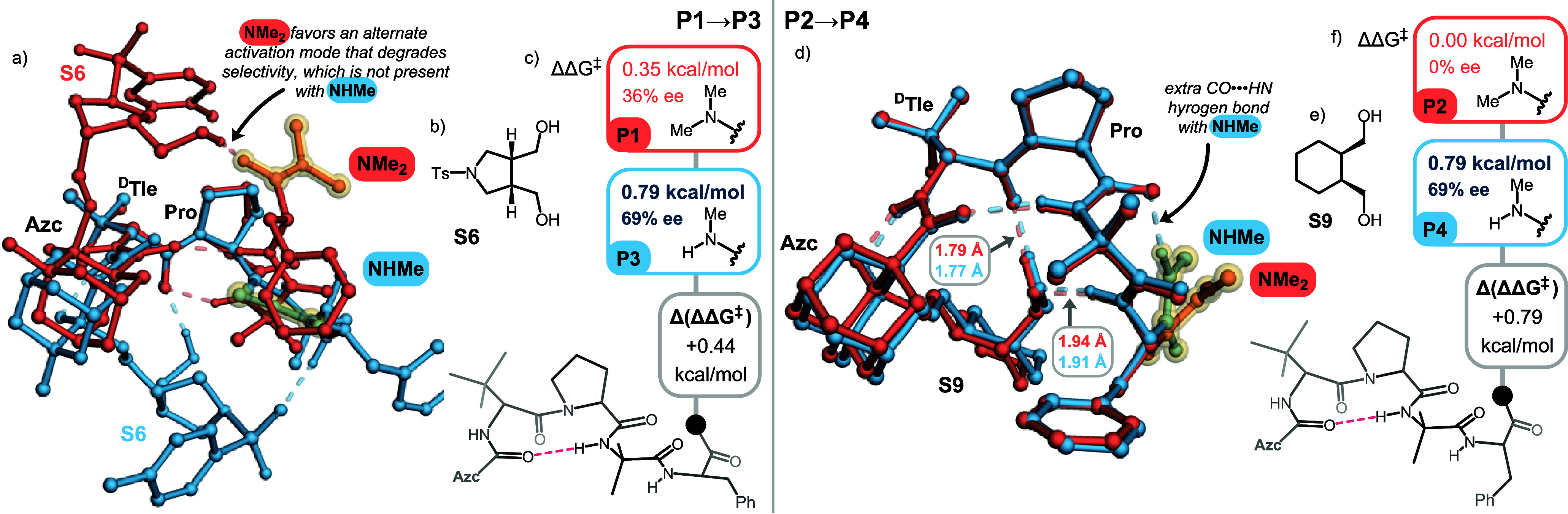
*C*-terminus cap effect: generational improvement from **P1** to **P3** (a–c) and from **P2** to **P4** (d–f). The free energy of activation differences (ΔΔ*G* ^‡^_obs_) was calculated from experimental selectivities (−40 °C). (a) Overlay of the divergent activation modes for the *minor* diastereomer of **P1** and **P3** with **S6**, with the latter featuring a spurious mode from the catalyst top face. (b) Diol **S6** used in the modeling. (c) Structural differences of **P1** and **P3** and their effect on the stereoselectivity in desymmetrizing **S6**. (d) Overlay of the divergent activation modes for the *major* diastereomer of **P2** and **P4** with **S9**. The additional hydrogen bond donor of **P4** tightens the second alcohol noncovalent interactions and reduces the complexity of the conformational space. (e) Diol **S9** used in the modeling. (f) Structural differences of **P2** and **P4** and their effect on the stereoselectivity in desymmetrizing **S9.**.

The same point change was even more beneficial for **P2**, a diastereomer of **P1** featuring the opposite absolute configuration of Phe (Figure 2d–f). Substrate **S9** improved from 0% *ee* with **P2** to a remarkable 69% *ee* with **P4** by the effect of this modification alone (+0.79 kcal/mol improvement in ΔΔ*G* ^‡^_obs_). The effect of the additional hydrogen bond donor was traced back to imparting a higher degree of preorganization to the interaction mode, leading to the major enantiomer of the product, by virtue of an extra hydrogen bond of the catalyst backbone with *itself*. This modification not only resolved conformational landscape for the interaction of **S9** with the catalyst (major enantiomer, from three to only one state within 2 kcal/mol from the most stable) but also tightened the internal network of hydrogen bonds between the catalyst and the free alcohol moiety of the substrate (Figure 2d).

### **P3** to **P4** (**S4**): ^D^Phe to ^L^Phe

Inversion of stereochemistry at the *i* + 3 position proved pivotal at this stage and afforded **P4**. The great success of **P4** across the diols set arose from combining an end-cap modification (NMe_2_ to NHMe, as from **P1** to **P3**) with the inversion of the configuration of Phe (^D^Phe to ^L^Phe, **P3** to **P4**). We assessed the influence of the phenylalanine configuration modeling substrate **S4**, which was desymmetrized in just 1% *ee* with **P3** but improved drastically to 77% *ee* with **P4** (+0.93 kcal/mol improvement in ΔΔ*G* ^‡^_obs_, Figure 3). Notably, while the interaction modes leading to the major enantiomer of the product feature identical hydrogen bond topology, **P4** features a much tighter binding pocket compared to **P3**, which instead adopts a relatively flat conformation. In addition, the accessible conformational space is also significantly reduced (major enantiomer complex, 3 kcal/mol window: **P3**: 14 conformers, **P4**: 3 conformers).

**Figure 3:**
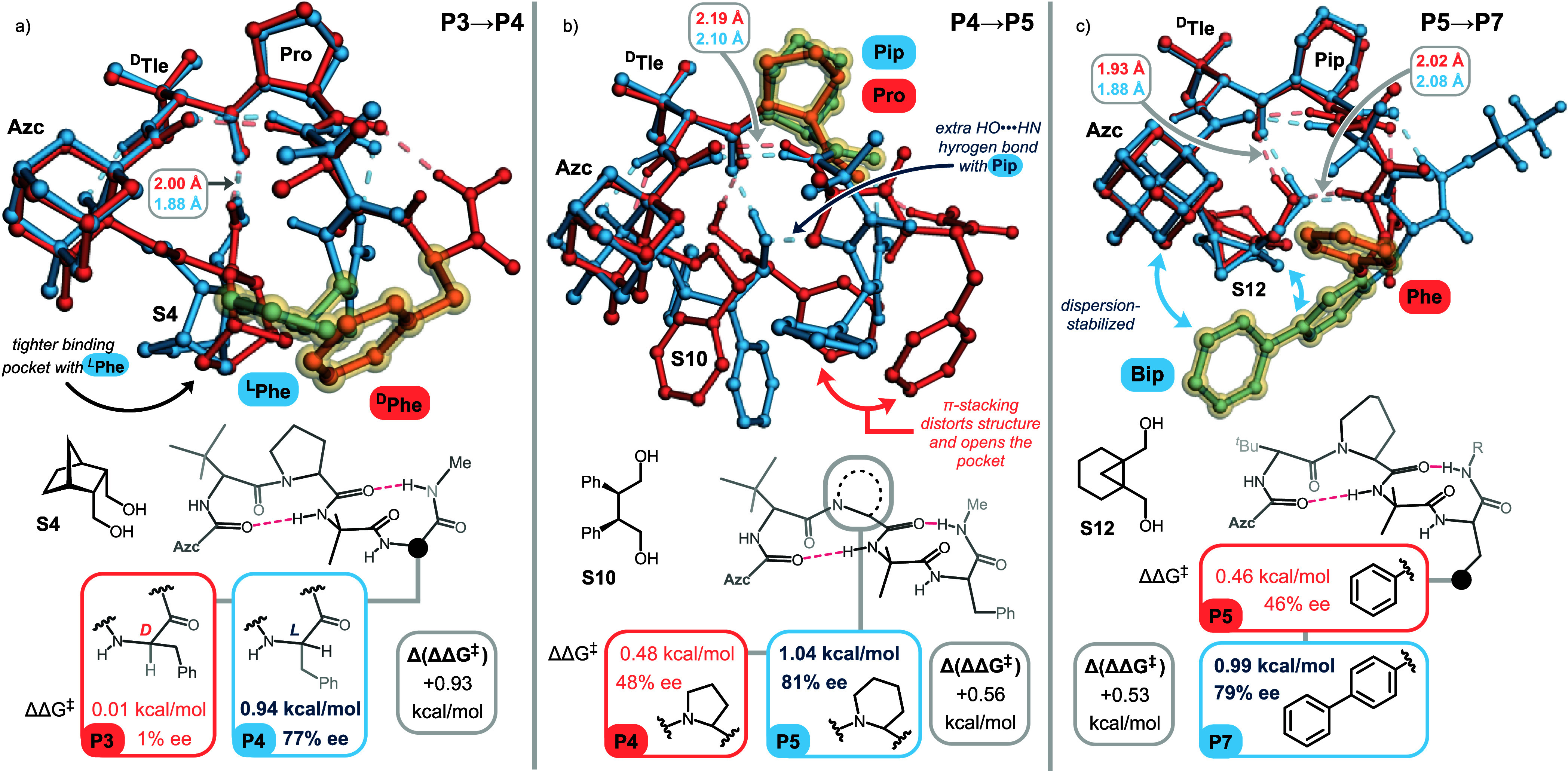
Three major catalyst improvements: from **P3** to **P4** (a), from **P4** to **P5** (b), and from **P5** to **P7** (c). Free energy of activation differences (ΔΔ*G* ^‡^_obs_) calculated from experimental selectivities (−40 °C). (a) Overlay of the activation modes for the *major* diastereomer of **P3** and **P4** with **S4**. While **P3** (^D^Phe) features a relatively flat double β-turn, **P4** (^L^Phe) shows a more enclosed geometry that interacts with the substrate more tightly. This is also reflected in the shorter hydrogen bond distances with the free alcohol of **S4**. (b) Overlay of the activation modes for the *major* diastereomer of **P4** and **P5** with **S10**. While **P4** (Pro) is more flexible and the favored conformation of the adduct gives up the enclosed, nonplanar geometry of the double β-turn in exchange for substrate π-stacking, **P5** (^L^Pip) features a stricter geometrical enforcement of the out-of-plane secondary structure and retains a conformation similar to the ones previously observed for other substrates. This is also reflected in the shorter hydrogen bond distances with the free alcohol of **S10**. (c) Overlay of the activation modes for the *major* diastereomer of **P5** and **P7** with **S12**. The larger polarizable surface of Bip (**P7**) relative to Phe (**P5**) induces a tighter fit of the pedant substituent thanks to attractive dispersive interactions with the adamantyl core of the **Azc** residue. This is also reflected in shorter NH···OH hydrogen bond distances with the free alcohol of **S10**.

### **P4** to **P5**: Pro to Pip

The next significant advancement in the generality of **P4** was obtained when replacing Pro with Pip (**P5**, Figure 3b). Analyzing the interactions of **S10**, which improved from 48% *ee* with **P4** to 81% *ee* with **P5** (+0.56 kcal/mol improvement in ΔΔ*G* ^‡^_obs_), we noted that the increased propensity of Pip to nucleate β-turns at the *i* + 2 position was also reflected in an increased rigidity of the catalyst structure against substrate-induced deformations. This can also be observed in the flattening (and shortening) of the β-turn hydrogen bond. In the case of **S10**, we attribute the success of the point change to the additional hydrogen bond between the phenylalanine N–H bond and the nonreacting −OH group present in the complex leading to the major enantiomer of the product (Figure 3b). This is not present in any accessible state of the **P4**/**S10** complex. We believe this is not present for **P4** due to the increased flexibility of Pro relative to Pip that allows the “flattening” of the catalyst geometry in exchange for the π-stacking interaction with aromatic substrate **S10.**

### **P5** to **P7**: Phe to Bip

Notwithstanding the obtainment of a quite general catalyst for diol desymmetrization (**P5**, up to 95% *ee*, 74% median *ee*), further optimization was still undertaken. Notably, not only was further improvement achieved, but we were amazed to observe that some point changes still proved beneficial across the *entirety* of the test set. The last of such modifications was the replacement of Phe for Bip (phenylalanine to biphenylalanine, **P5** to **P7**, Figure 3c). Substrate **S12** was desymmetrized in 46% *ee* with **P5** and in 79% *ee* with **P7** (+0.53 kcal/mol improvement in ΔΔ*G* ^‡^_obs_). Analysis of the interactions leading to the major enantiomer of the product highlighted how Bip induces subtle but important geometrical alterations of the binding pocket. Chiefly, it does so by shielding a region of space in front of the Azc residue by virtue of the association of two dispersion energy-donating groups: the biphenyl and adamantyl moieties. Moreover, numerous C–H bonds on the substrate are within 3.5 Å of carbon atoms of the Bip residue, engaging in additional weak interactions. This pocket deformation also causes a significative contraction and a slight elongation, respectively, of the two key hydrogen bonds involving the free alcohol moiety of substrate **S12**. It is noteworthy that while the interaction modes of both **P5** and **P7** with **S12** show three conformations within 3 kcal/mol (G_233 K_), only one of these is transition state-like (i.e., features the catalyst’s N^+^–O^–^ moiety next to an alcohol C–H bond).

### Interactions of Different Diols with **P7**

After observing the convergence of many substrates to a unified interaction topology across the different catalyst generations, we wanted to assess if multiple substrates that are effectively desymmetrized by the optimized catalyst **P7** also favor the same interaction network, in addition to the already modeled **S12**. Interestingly, we observed different preferred interaction modes for different substrates (Figure 4). Certain constrained, cyclic diols like **S4** and **S15** bind with a slight alteration of this main mode, trading one of the hydrogen bonds (alcohol (O)–H to amide (C)=O) for a better overall geometrical fit within the pocket (Figure 4a, “incomplete” mode). This remarkable effect causes conformations of bound diols **S4** and **S15** that have one *less* hydrogen bond relative to the “main” mode to be 5 to 6 kcal/mol *more* stable than the latter. An “alternate” mode could also be observed as the preferred mode for the minor diastereomer of **P7** with **S10**. Yet, most rigid substrates retain high levels of selectivity regardless of their preferred interaction network (96% *ee* for **S4** and 90% *ee* for **S10**). Evidently, this activation mode promiscuity is not necessarily tied to a degradation in selectivity as long as the coexistence of multiple modes retains a large energetic splitting between the diastereomeric states (Δ*G*° = 7.71 kcal/mol for **S4** and 5.98 kcal/mol for **S10** between the most stable diastereomeric TS-like ground-state conformers).

**Figure 4:**
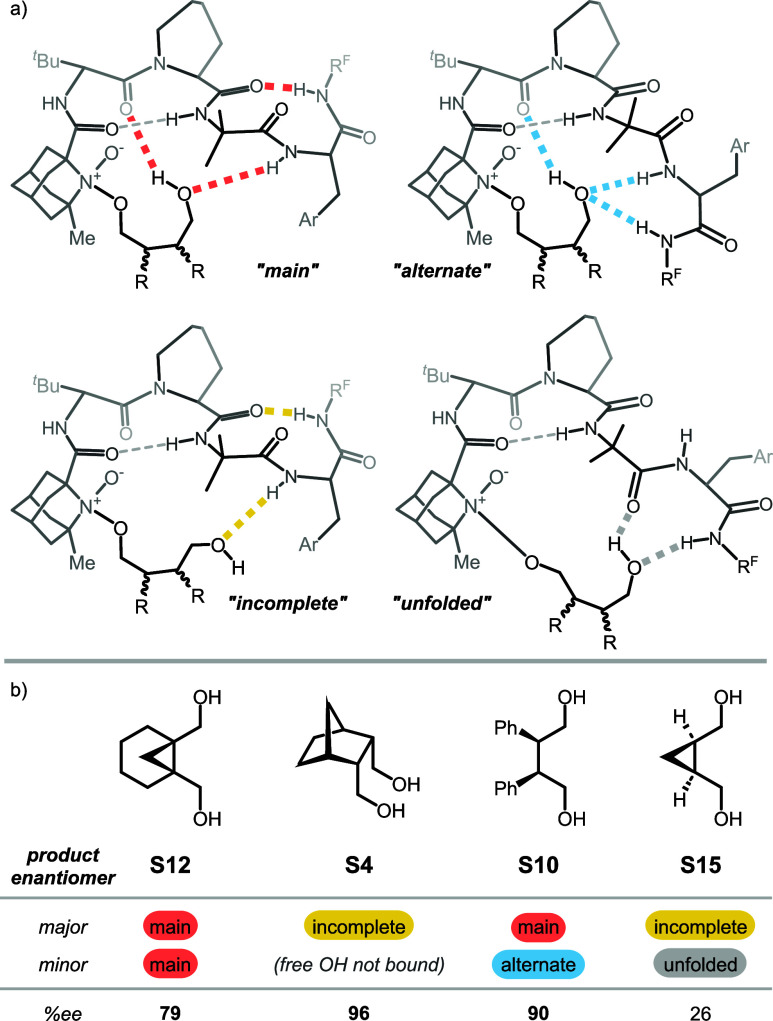
Analysis of the preferred interaction modes of **P7** with different substrates from computational modeling. (a) Schematic representation of the different modes. (b) Most stable modes for each diastereomer of the reported substrates with **P7** (“major” oxoammonium alkoxide diastereomer leading to the major observed enantiomer of the lactone product in the original work^8^ and *vice versa*).

On the contrary, **P7** performs poorly on substrate **S15** (26% *ee*). In the original report, a rationale was put forward discussing the low buried volume of this substrate inside the binding pocket.^8^ The atomistic modeling corroborates and complements this analysis by also unveiling how this substrate features the presence of a previously unobserved “unfolded” interaction mode, where the catalyst second β-turn hydrogen bond is lost (Figure 4). Substrate **S15** also showed less energetically differentiated states (Δ*G*° = 2.36 kcal/mol between the most stable diastereomeric TS-like ground-state conformers). It is noteworthy that while all of the modeled complexes feature the same number of hydrogen bonds in the major and minor diastereomers, substrate **S4** features one more hydrogen bond in the major. This is consistent with the substrate’s superior performance in the reaction (**P7**/**S4**, 96% *ee*, see Figure S7 for the three-dimensional superposition of the interaction modes). While a quantitative energy decomposition analysis^39–41^ would tease out the main components of Δ*G*° for different substrate–catalyst complexes, we limit this work to a more qualitative description of the noncovalent interaction networks. Moreover, our analysis emphasizes the likely contribution of multiple members of a transition state ensemble to overall selectivity and the feature that different interaction modes within the ensemble may contribute to varying degrees for different substrates.

### Application to Regioselective Diol Oxidations

Recently, Lin's group^42^ reported the use of an oxo-analog of **P7** (**OAzcP7**, Figure 5) to enable the regiocontrolled oxidation of unsymmetrical diols. In this reaction, the highly preorganized peptidyl template enabled the selective oxidation of the more hindered alcohol group over the more accessible group, overriding intrinsic steric biases. The authors rationalized this selectivity with the ability of **OAzcP7** to selectively sequester the more accessible primary alcohol within the sterically demanding hydrogen bond peptidyl framework, with the more hindered alcohol available to form a more stable oxoammonium alkoxide adduct (Figure 5a, blue). This heuristic is in alignment with the ground-state computational modeling we conducted on representative substrate **D2** (Figure 5b), in analogy to the rest of the catalyst–substrate pairings of this work. The energetic difference between the best conformer of each interaction mode is 1.86 kcal/mol, which is in qualitative agreement with the experimental selectivity obtained (Δ*G*°_calc., 228 K_ = 1.86 kcal/mol, ΔΔ*G* ^‡^_obs., 228 K_ ∼ 2.0 kcal/mol). The favored interaction mode also features a hydrogen bond network involving the unbound alcohol group of the substrate that is strengthened relative to the unfavored regioisomeric complex (Figure 5a, blue structure featuring a tighter hydrogen bond network relative to orange).

**Figure 5:**
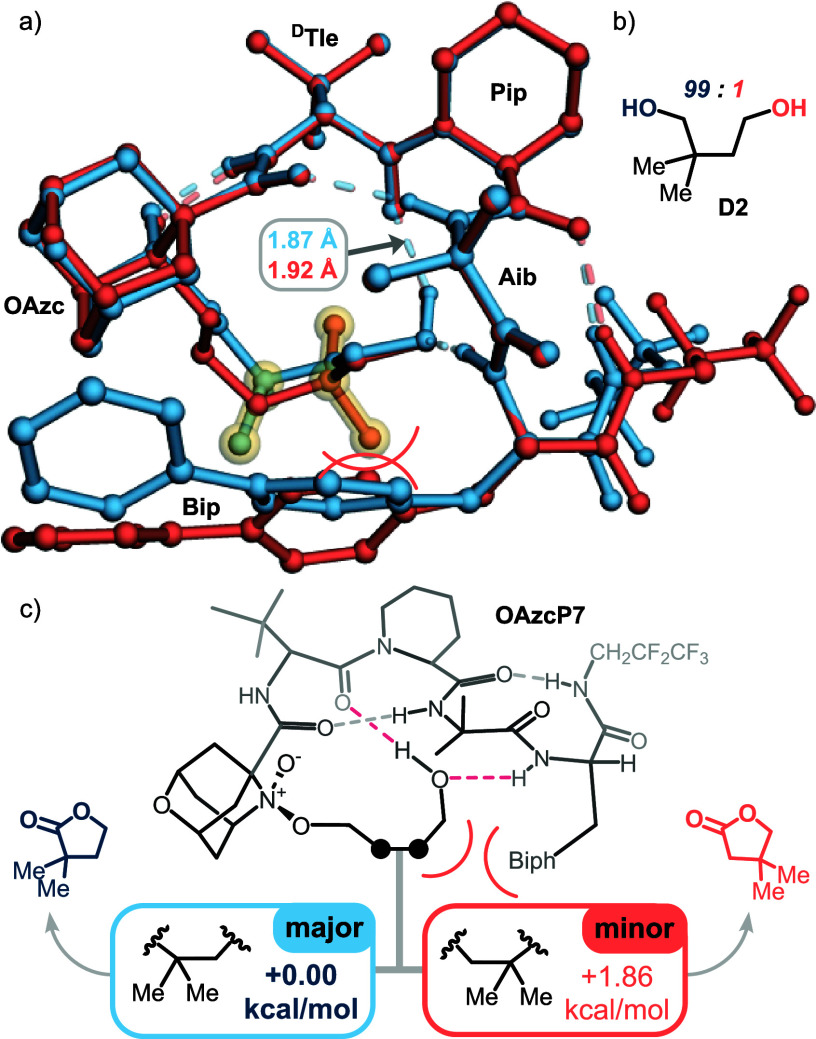
Application of the rigid, preorganized peptidic template of **P7** to the regioselective oxidation of primary alcohols. The secondary structure of the catalyst (**OAzcP7**) is highly sensitive to steric effects, and the preferential noncovalent interaction of the *least* hindered alcohol with the peptide backbone results in the formation of the covalent oxoammonium adduct of the *most* hindered alcohol, resulting in contrasteric oxidation. (a) Overlay of the best interaction modes for the different regioisomers of diol adducts. (b) Substrate **D2** used in the modeling. (c) Structural differences of the regioisomeric adducts and effect on the regioselectivity of the oxidation. Energetic values are computational relative energies of regioisomeric oxoammonium alkoxide ground states (Δ*G*°, −40 °C).

## Conclusions

In conclusion, we described our approach to the experimental optimization of an asymmetric *meso*-diol oxidation catalyst toward high generality. We then performed a large-scale DFT modeling of many catalyst–substrate pairs (∼6000 structures) to unearth the underpinnings of each generational catalyst improvement, as well as the drivers behind the selectivity of the optimized catalyst toward different substrates. Highlights of the modeling include the clarification of the conformational evolution throughout the catalyst generations, divergence of catalytically relevant conformations from those observed in several catalysts’ crystal structures, and unexpected diversity of catalyst–substrate interactions that might not have been expected with a “general” catalyst. Our results show that catalyst flexibility and the coexistence of different catalyst–substrate interaction modes for different substrates are *not* at odds with the goals of enantioselectivity and substrate generality. In fact, an alternative perspective on the system may relate to the concept of “confinement”.^43^ In fact, enclosed environments seem to facilitate the energetic splitting between multiple diastereomeric or topologically different states regardless of their exact nature, which can in fact vary across substrates, while still contributing to high selectivity.

## Supplementary Material



## Data Availability

The .xyz files containing the computational coordinates for these ensembles can be found on the Github repository for this work

## Notes

Experimental details, computational methodologies, and energies for final DFT ensembles (PDF)

## ABBREVIATIONS

Acpc: aminocyclopropane carboxylic acid
Aib: aminoisobutyric acid
Bip: biphenylalanine
Pip: pipecolic acid
Phe: phenylalanine
Pro: proline
RMSD: root-mean-squared deviation (of atomic positions, in Å)
Tic: tetrahydroquinoline carboxylic acid
