# Supplementary material for: Experimental Lineage and Computational Analysis of a General Aminoxyl-Based Oxidation Catalyst: Generality from Substrate-Specific Interactions
Source: ACS Catal. 2025 Oct 7;15(20):17548–57. doi: 10.1021/acscatal.5c05893 (PMC12539552; doi:10.1021/acscatal.5c05893)
Supplement: Supplementary file 1 [file cs5c05893_si_001.pdf]

Supporting Information for

Experimental Lineage and Computational Analysis of a General Aminoxyl-based Oxidation Catalyst: Generality from Substrate-Specific Interactions

Soren D. Rozema[‡1], Nicolò Tampellini[‡1], Jonas Rein[2], Matthew S. Sigman[3], Song Lin[2], Scott J. Miller*[1]

[1]Department of Chemistry, Yale University, New Haven, CT 06520, USA
[2]Department of Chemistry and Chemical Biology, Cornell University, Ithaca, NY 14853, USA
[3]Department of Chemistry, University of Utah, Salt Lake City, UT 84112, USA

[‡]These authors contributed equally and are listed in alphabetical order.

Correspondence: Scott J. Miller
scott.miller@yale.edu

Table of Contents

1. Practical considerations....2
2. Cyclic Voltammetry Studies....2
2. Computational Workflow....7
 2.1. Diol Oxidation: Catalyst Optimization Energetic Dataset....8
 2.2. TS-like ensembles....21
 2.3. Ensemble sizes....26
 2.4. Additional P7 modeling....27
3. Additional figures....31
4. References....32

1. Practical considerations

All aminoxyl catalysts based on the AzcMe core reported in this work were bench-stable solids that could be handled and stored at room temperature without any special precautions to protect them from air or moisture. The catalysts, whether stored as solids or as stock solutions in DCM, showed no change in activity over the course of our studies (approximately 12 months). Based on our experience with aminoxyl-peptides, we anticipate that **P7** may be stored indefinitely at room temperature; however, no long-term studies (>12 months) have been conducted. Three batches of **P7** were prepared. As reported in the original work, the catalysts were isolated as a mixture of aminoxyl radical and hydroxylamine (both on-cycle species) with the ratio showing some variation across batches. The selectivity of the catalyst batches showed no statistically significant differences in systematic comparisons. **P7** and other aminoxyl catalysts were isolated via reverse phase chromatography on C8 modified silica but also demonstrated good stability on regular silica using both DCM/MeOH and EtOAc/Hex mixtures as eluents.

2. Cyclic Voltammetry Studies

To gain further insight into the mode of enantioinduction, competition studies of the oxidation of diols over simple alcohols were conducted. Specifically, we hoped to understand if highly selective catalyst provided an acceleration of the reaction rate for diol oxidation or if the catalyst preferentially oxidized the diol due to enhanced stability of the diol-alkoxide-oxoammonium complex when compared to the analogous alkoxide-oxoammonium complex arising from a simple alcohol.

This was achieved by comparing the rate differences between oxidation of **$CyCH_2OH$** and **S9** (cyclohexane-1,2-diyldimethanol) in independent rate measurements. To do so we carried out cyclic voltammetry (CV) experiments using a rotating disk electrode (RDE).

Methods: All cyclic voltammetry studies were conducted inside the glovebox with the BASi Epsilon. Measurements were performed in 0.1 M $TBAPF_6$ in DCE using an RDE-2 Cell Stand and a glass cell vial. The working electrode is a 3 mm diameter glassy carbon working electrode for RDE and was polished with 1.0, 0.3 and 0.05 μm aluminum oxide and then sonicated in distilled water and acetone then air dried before each scan. An $Ag/AgNO_3$ (0.01 M in MeCN (0.1 M TBAPF6)) reference electrode is used. After every batch of experiments (variation of rotation frequency) the final potential is referenced to the ferrocenium/ferrocene redox couple ($Fc^{+/0}$). A platinum wire that is sonicated in acetone and then burnt for 10 seconds with a butane torch is used as a counter electrode. Cyclic voltammograms were acquired with a scan rate of 5 mV/s and a positive scan direction.

A stock solution containing 150 mM 2,6-di-tert-butylpyridine in 0.1 M $TBAPF_6$ in DCE was prepared. A cyclic voltammogram was acquired which served as the background and was subtracted from subsequent cyclic voltammograms.

The data was acquired following analogous methodologies in the literature.[1, 2]

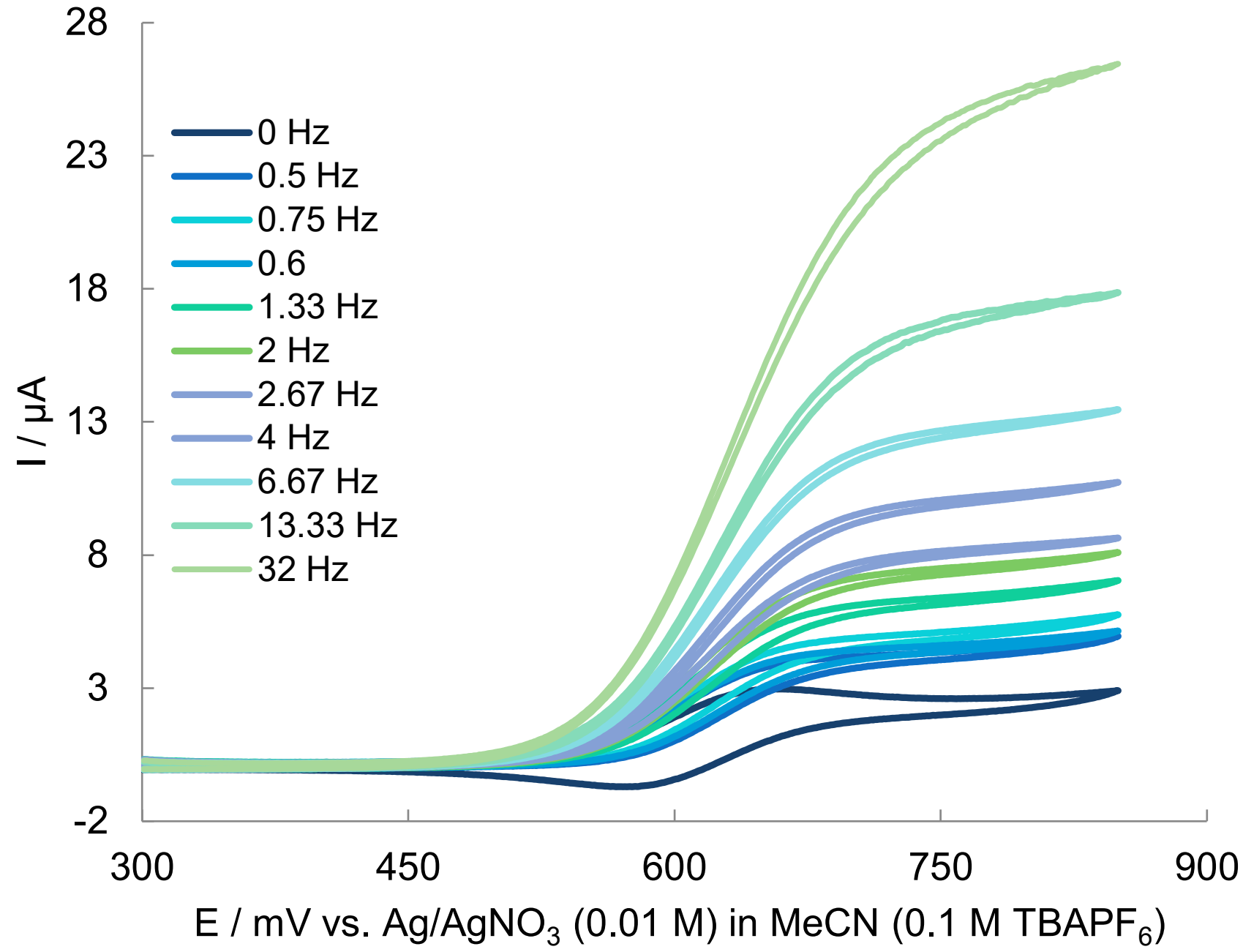

Figure S1. Cyclic voltammograms of 0.1 mM **P7** in 150 mM 2,6-di-tert-butylpyridine in 0.1 M $TBAPF_6$ in DCE.

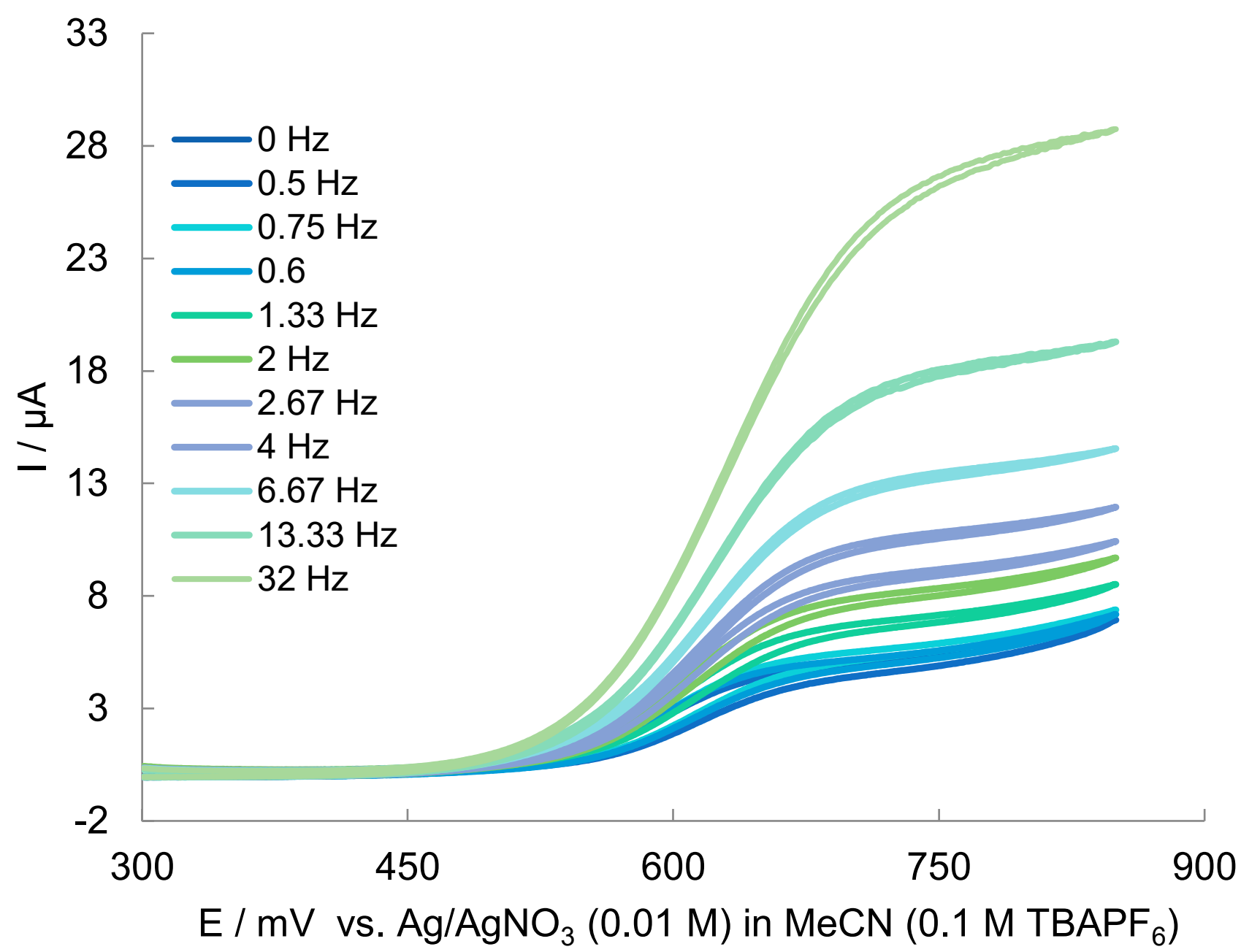

Figure S2. Cyclic voltammograms of 0.1 mM **P7** with 50 mM of **S9** in 150 mM 2,6-di-tert-butylpyridine in 0.1 M $TBAPF_6$ in DCE.

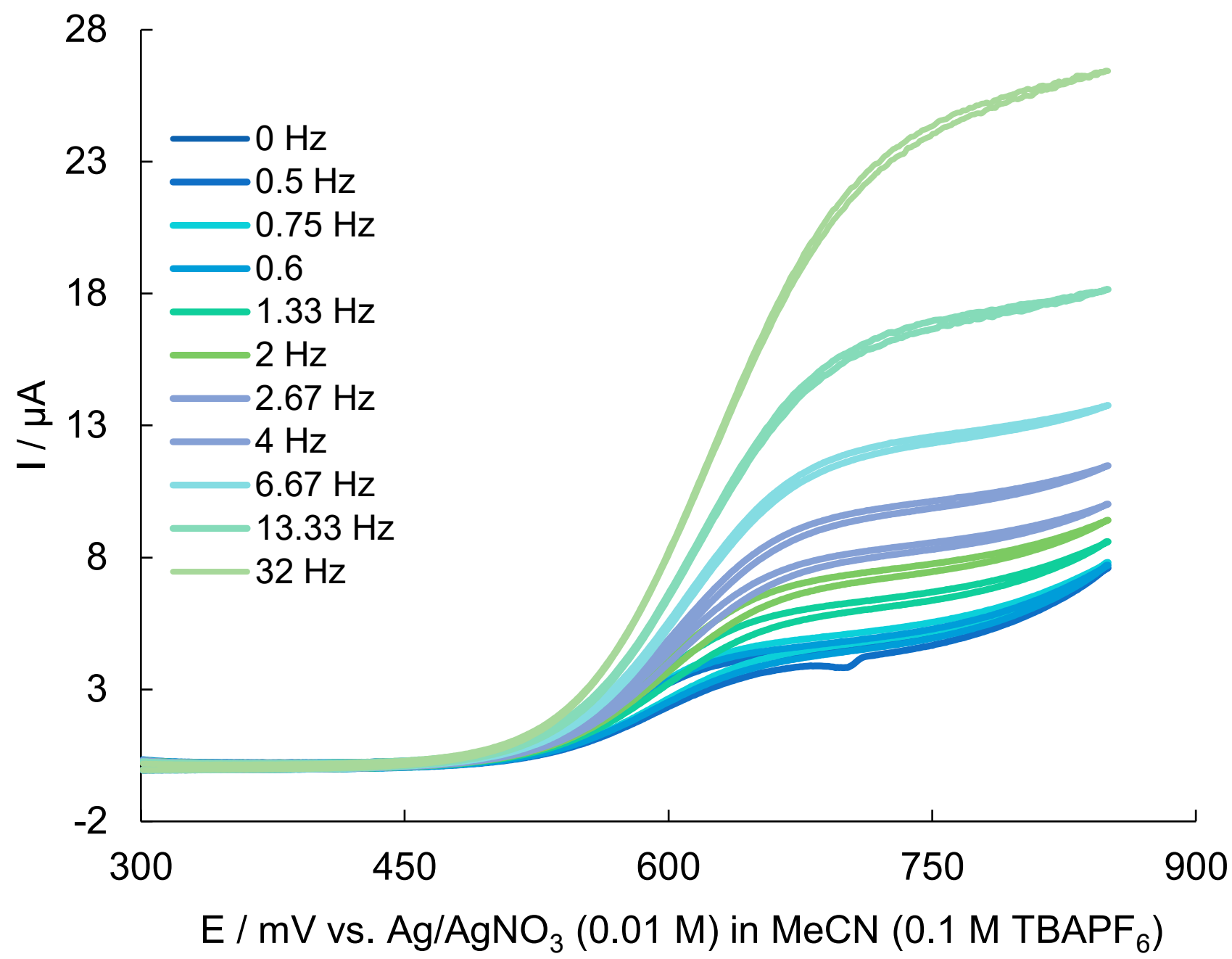

Figure S3. Cyclic voltammograms of 0.1 mM **P7** with 50 mM of **$CyCH_2OH$** in 150 mM 2,6-di-tert-butylpyridine in 0.1 M $TBAPF_6$ in DCE.

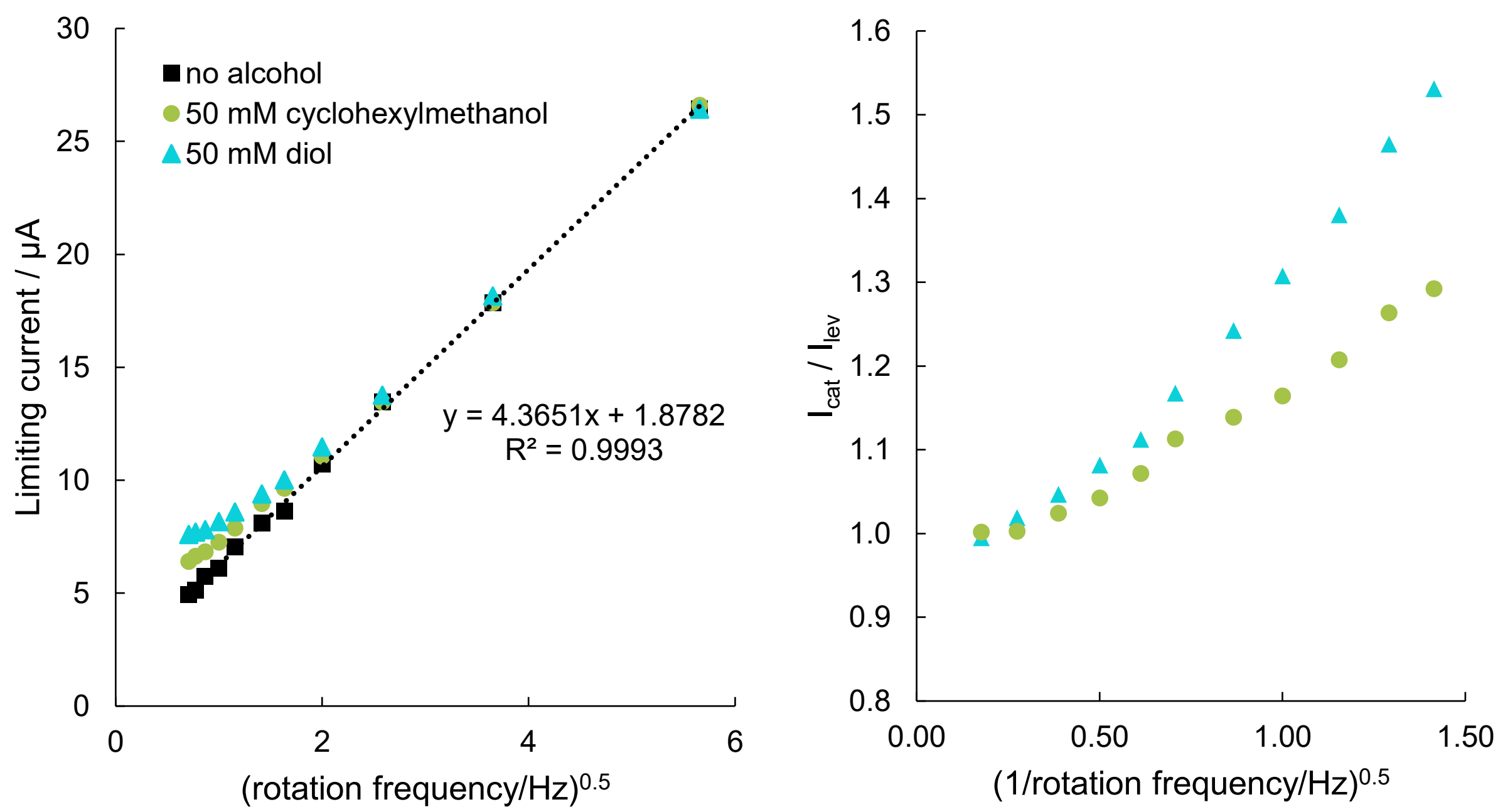

Figure S4. (Right) Levich plot of **P7** without addition of alcohol plotting the limiting current against the square root of the rotation frequency. The same measurements were performed in the presence of **P9** and **$CyCH_2OH$**. The deviation from the Levich plot is due to catalytic turnover and is correlated to the rate of oxidation. (Left) Plot of the limiting current in the presence of each alcohol (I_{cat}) divided by the limiting current measured without alcohol in the Levich plot (I_{lev}).

The ratio of limiting current in the presence of each alcohol (I_{cat}) divided by the limiting current measured without alcohol in the Levich plot (I_{lev}) can be related to the second order rate constants *k* of the alcohol oxidation by **P7**:[1, 3]

$$\frac{I_{cat}}{I_{lev}} = z coth(z)$$

where $z = 1.61 \left(\frac{v}{D}\right)^{1/6} \left(\frac{k c_{P7}}{\omega}\right)^{1/2}$, *v* is the kinetic viscosity of the solution, D is the diffusion constant of **P7**, c_{P7} is the concentration of **P7**, and ω is the rotation frequency. The data presented in **Figure S4** was then fitted with using *OriginPro 2022* with the following general equation.

$$B = \frac{I_{cat}}{I_{lev}} = aA coth(aA)$$

where $a = 1.61 \left(\frac{v}{D}\right)^{1/6} (k c_{P7})^{1/2}$ and A = $\left(\frac{1}{\omega}\right)^{1/2}$:

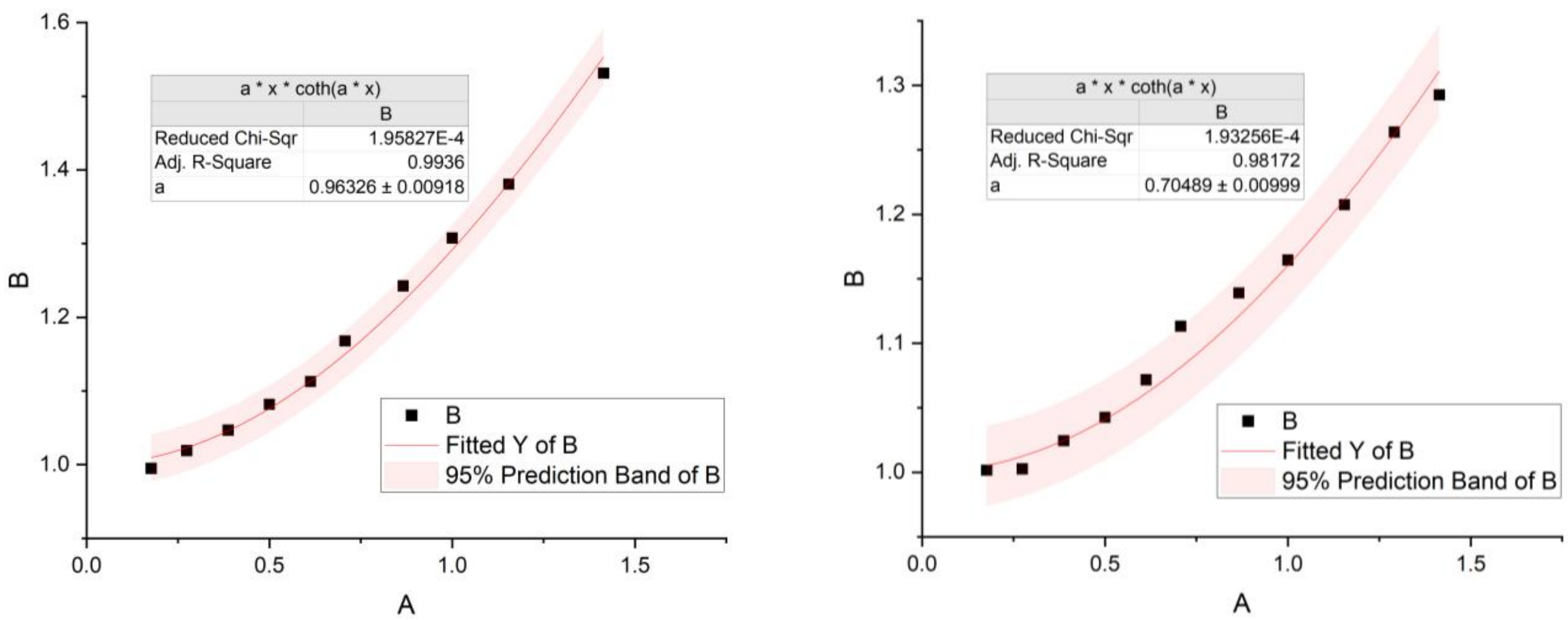

Figure S5. (Right) Fit obtained with **S9**. (Left) fit obtained with **CyCH₂OH**.

With this the relative second order rate constants can be calculated:

$$\frac{a_{S9}}{a_{CyCH_2OH}} = \frac{(k_{S9})^{1/2}}{(k_{CyCH_2OH})^{1/2}}$$

The independent measurement of the rate constants reveals that the oxidation of **S9** by **P7** is 1.87 ± 0.09 times faster than the oxidation of **$CyCH_2OH$** by **P7**. Given that in the competition experiment S9 is preferentially oxidized with selectivity coefficients s of up to 26.8, this provides further evidence for the formation of an alkoxide-oxoammonium complex and indicates that the reaction is governed by saturation kinetics. Thus, the high selectivity observed for the oxidation **S9** over **$CyCH_2OH$** can be attributed to the formation of a more alkoxide-oxoammonium complex presumably due to hydrogen bonding of the free hydroxyl group to the peptide backbone.

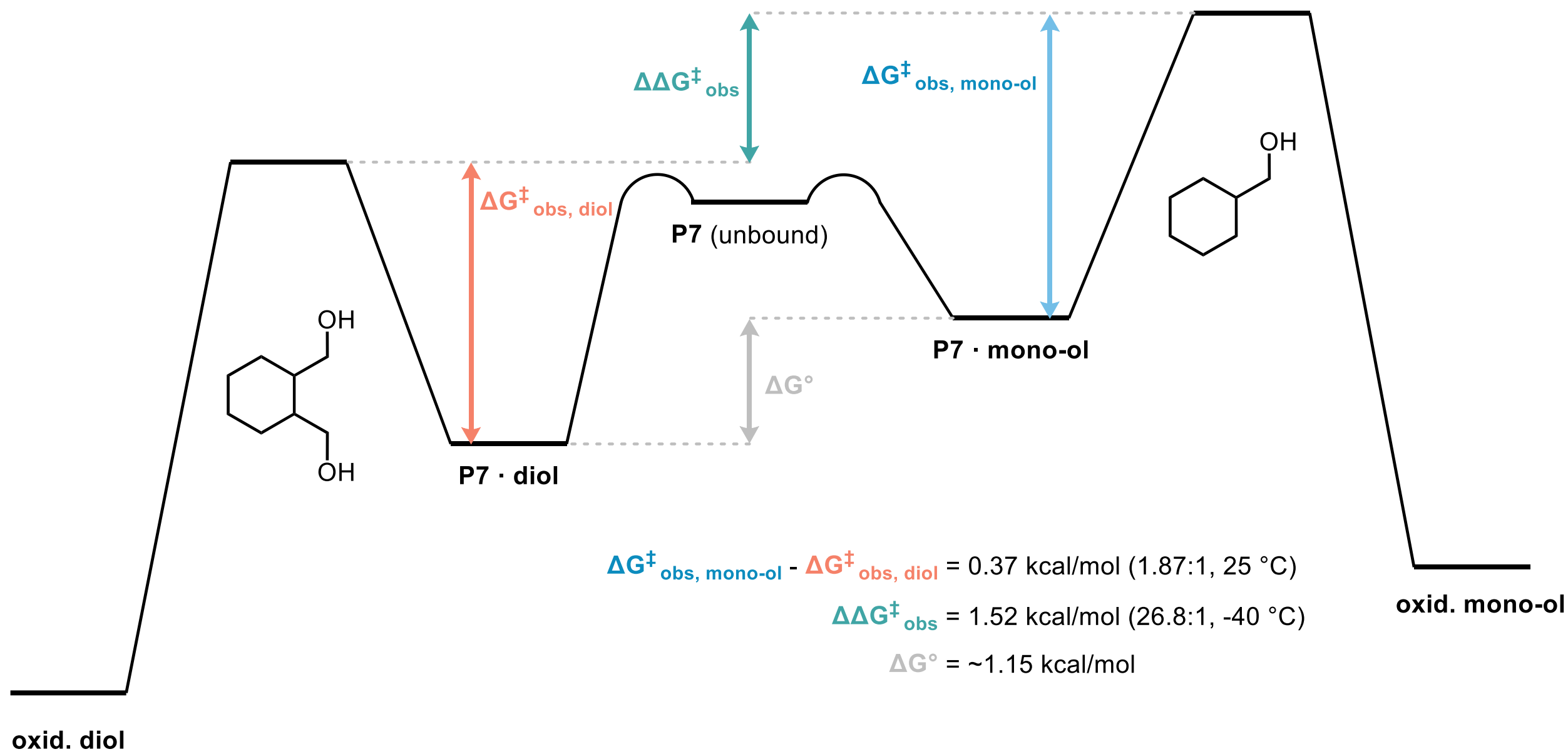

Figure S6. Potential energy surface for the competition experiment composed with experimental electroanalytical rate data. Free energy values calculated with the Boltzmann equation with T=25 °C (independent rate measurements) and T=-40 °C (competition experiment). For similar $\Delta G^{\ddagger}$ values (orange and blue), ΔG° (grey) becomes a good proxy for $\Delta\Delta G^{\ddagger}_{obs}$ (green).

2. Computational Workflow

Initial geometries for the covalent adducts were obtained using the Avogadro GUI.[4] Every complex was modeled using the same absolute configuration for *tert*-leucine (*D* amino acid, *i.e. R* absolute configuration) for ease of comparison.

Conformational searches were performed with CREST[5] 2.12 at the GFN2-xTB[6]//GFN-FF[7] level using the ALPB solvation model[8] for dichloromethane. The resulting conformational ensembles were refined via FIRECODE[9] 1.0.0 at the GFN2-xTB/ALPB(CH_2Cl_2) level (RMSD threshold = 0.5 Å, energy window = 10 kcal/mol).

These ensembles were then subjected to a single-point energy calculation at the R^2SCAN-3c[10-12]/CPCM[13](CH_2Cl_2) level via ORCA 5.0.4[14, 15] (keywords: Defgrid3). Structures within 5 kcal/mol (Electronic Energy) from the most stable were then optimized at the same level of theory. Vibrational analysis was carried out to confirm the nature of the stationary points (zero imaginary modes for equilibrium geometries, one imaginary mode for transition states) and to extract thermochemical corrections at the temperature of -40 °C (233.15 K). Structures within 3 kcal/mol from the most stable (Free Energy, -40 °C, R^2SCAN-3c/CPCM(CH_2Cl_2)), after a similarity pruning process via FIRECODE (RMSD threshold = 0.5 Å), were subjected to single-point energy calculations at the ωB97M-V[16]/def2-TZVPP[17]/SMD[13](CH_2Cl_2) level of theory. The free energies used for qualitative comparisons are therefore obtained by the composite method ωB97M-V/def2-TZVPP/SMD(CH_2Cl_2)//R^2SCAN-3c/CPCM(CH_2Cl_2) adding the electronic energy from the high-level single point calculation to the vibrational correction for free energy at the lower level.

All `.xyz` geometries are available on the Github repository of this work.

2.1. Diol Oxidation: Catalyst Optimization Energetic Dataset

Complete ensembles for the modeled pairs of peptides and substrates [P1, S6, reported absolute configuration (major enantiomer) = `1r6.xyz`; minor (enantiomer) configuration = `1e6.xyz`]. Ensembles were pruned for similarity and for energy at 10 kcal/mol from the most stable [G_{233K}, ωB97M-V/def2-TZVPP/SMD(CH_2Cl_2)//R^2SCAN-3c/CPCM(CH_2Cl_2)]. Following, smaller ensembles are presented, composed of structures within 2.0 kcal/mol from the most stable and removing non-ts-like ground state structures ($d_{O\text{-}H}$>3 Å between oxoammonium alkoxide O^- and diol CH_2 hydrogen atom). These smaller ensembles are named "ts-like" (*i.e.* `1r6_ts-like.xyz`). `SP_EE(Eh)` is the single point energy at the ωB97M-V/def2-TZVPP/SMD(CH_2Cl_2) level, in Hartree. `gcorr(Eh)` is the Free Energy correction (G-EE) at the R^2SCAN-3c/CPCM(CH_2Cl_2) level, in Hartree. `G(Eh)` is the Free Energy at the ωB97M-V/def2-TZVPP/SMD(CH_2Cl_2)//R^2SCAN-3c/CPCM(CH_2Cl_2) level, in Hartree. `Rel. G.` is the relative Free Energy at the same level, in kcal/mol.

1e6.xyz

```
1  SP_EE(Eh)=-3483.15969723, gcorr(Eh)=1.13614343, G(Eh)=-3482.02355380, Rel. G(kcal/mol)=0.00
2  SP_EE(Eh)=-3483.15987057, gcorr(Eh)=1.13649565, G(Eh)=-3482.02337492, Rel. G(kcal/mol)=0.11
3  SP_EE(Eh)=-3483.15424501, gcorr(Eh)=1.13359522, G(Eh)=-3482.02064979, Rel. G(kcal/mol)=1.82
4  SP_EE(Eh)=-3483.15382644, gcorr(Eh)=1.13485200, G(Eh)=-3482.01897444, Rel. G(kcal/mol)=2.87
5  SP_EE(Eh)=-3483.15212884, gcorr(Eh)=1.13354924, G(Eh)=-3482.01857960, Rel. G(kcal/mol)=3.12
6  SP_EE(Eh)=-3483.15291296, gcorr(Eh)=1.13464316, G(Eh)=-3482.01826980, Rel. G(kcal/mol)=3.32
7  SP_EE(Eh)=-3483.15250347, gcorr(Eh)=1.13450035, G(Eh)=-3482.01800312, Rel. G(kcal/mol)=3.48
8  SP_EE(Eh)=-3483.15403000, gcorr(Eh)=1.13634282, G(Eh)=-3482.01768718, Rel. G(kcal/mol)=3.68
9  SP_EE(Eh)=-3483.15179960, gcorr(Eh)=1.13504489, G(Eh)=-3482.01675471, Rel. G(kcal/mol)=4.27
10 SP_EE(Eh)=-3483.15198035, gcorr(Eh)=1.13596970, G(Eh)=-3482.01601065, Rel. G(kcal/mol)=4.73
11 SP_EE(Eh)=-3483.15127484, gcorr(Eh)=1.13686748, G(Eh)=-3482.01440736, Rel. G(kcal/mol)=5.74
12 SP_EE(Eh)=-3483.14833570, gcorr(Eh)=1.13509027, G(Eh)=-3482.01324543, Rel. G(kcal/mol)=6.47
```

1r6.xyz

```
1  SP_EE(Eh)=-3483.15808078, gcorr(Eh)=1.13465102, G(Eh)=-3482.02342976, Rel. G(kcal/mol)=0.00
2  SP_EE(Eh)=-3483.15708150, gcorr(Eh)=1.13488633, G(Eh)=-3482.02219517, Rel. G(kcal/mol)=0.77
3  SP_EE(Eh)=-3483.15579349, gcorr(Eh)=1.13490583, G(Eh)=-3482.02088766, Rel. G(kcal/mol)=1.60
4  SP_EE(Eh)=-3483.15691844, gcorr(Eh)=1.13653654, G(Eh)=-3482.02038190, Rel. G(kcal/mol)=1.91
5  SP_EE(Eh)=-3483.15471877, gcorr(Eh)=1.13463277, G(Eh)=-3482.02008600, Rel. G(kcal/mol)=2.10
6  SP_EE(Eh)=-3483.15538874, gcorr(Eh)=1.13587100, G(Eh)=-3482.01951774, Rel. G(kcal/mol)=2.45
7  SP_EE(Eh)=-3483.15462740, gcorr(Eh)=1.13520154, G(Eh)=-3482.01942586, Rel. G(kcal/mol)=2.51
8  SP_EE(Eh)=-3483.15378652, gcorr(Eh)=1.13479177, G(Eh)=-3482.01899475, Rel. G(kcal/mol)=2.78
9  SP_EE(Eh)=-3483.15453361, gcorr(Eh)=1.13629565, G(Eh)=-3482.01823796, Rel. G(kcal/mol)=3.26
10 SP_EE(Eh)=-3483.15355184, gcorr(Eh)=1.13537699, G(Eh)=-3482.01817485, Rel. G(kcal/mol)=3.30
11 SP_EE(Eh)=-3483.15438694, gcorr(Eh)=1.13644733, G(Eh)=-3482.01793961, Rel. G(kcal/mol)=3.45
```

12 SP_EE(Eh)=-3483.15290412, gcorr(Eh)=1.13531597, G(Eh)=-3482.01758815, Rel. G(kcal/mol)=3.67

13 SP_EE(Eh)=-3483.14900549, gcorr(Eh)=1.13288295, G(Eh)=-3482.01612254, Rel. G(kcal/mol)=4.59

14 SP_EE(Eh)=-3483.15212879, gcorr(Eh)=1.13602564, G(Eh)=-3482.01610315, Rel. G(kcal/mol)=4.60

15 SP_EE(Eh)=-3483.15013681, gcorr(Eh)=1.13410422, G(Eh)=-3482.01603259, Rel. G(kcal/mol)=4.64

16 SP_EE(Eh)=-3483.14652008, gcorr(Eh)=1.13503228, G(Eh)=-3482.01148780, Rel. G(kcal/mol)=7.49

17 SP_EE(Eh)=-3483.14591271, gcorr(Eh)=1.13551647, G(Eh)=-3482.01039624, Rel. G(kcal/mol)=8.18

2e9.xyz

1 SP_EE(Eh)=-2687.39080398, gcorr(Eh)=1.06239749, G(Eh)=-2686.32840649, Rel. G(kcal/mol)=0.00

2 SP_EE(Eh)=-2687.39231390, gcorr(Eh)=1.06446446, G(Eh)=-2686.32784944, Rel. G(kcal/mol)=0.35

3 SP_EE(Eh)=-2687.38987889, gcorr(Eh)=1.06232480, G(Eh)=-2686.32755409, Rel. G(kcal/mol)=0.53

4 SP_EE(Eh)=-2687.38816355, gcorr(Eh)=1.06114815, G(Eh)=-2686.32701540, Rel. G(kcal/mol)=0.87

5 SP_EE(Eh)=-2687.39063507, gcorr(Eh)=1.06489496, G(Eh)=-2686.32574011, Rel. G(kcal/mol)=1.67

6 SP_EE(Eh)=-2687.38927148, gcorr(Eh)=1.06358927, G(Eh)=-2686.32568221, Rel. G(kcal/mol)=1.71

7 SP_EE(Eh)=-2687.38756090, gcorr(Eh)=1.06317640, G(Eh)=-2686.32438450, Rel. G(kcal/mol)=2.52

8 SP_EE(Eh)=-2687.38743616, gcorr(Eh)=1.06401591, G(Eh)=-2686.32342025, Rel. G(kcal/mol)=3.13

9 SP_EE(Eh)=-2687.38561729, gcorr(Eh)=1.06306115, G(Eh)=-2686.32255614, Rel. G(kcal/mol)=3.67

10 SP_EE(Eh)=-2687.38695220, gcorr(Eh)=1.06455858, G(Eh)=-2686.32239362, Rel. G(kcal/mol)=3.77

11 SP_EE(Eh)=-2687.38669287, gcorr(Eh)=1.06462063, G(Eh)=-2686.32207224, Rel. G(kcal/mol)=3.97

12 SP_EE(Eh)=-2687.38497214, gcorr(Eh)=1.06340885, G(Eh)=-2686.32156329, Rel. G(kcal/mol)=4.29

13 SP_EE(Eh)=-2687.38266104, gcorr(Eh)=1.06285231, G(Eh)=-2686.31980873, Rel. G(kcal/mol)=5.40

14 SP_EE(Eh)=-2687.38378253, gcorr(Eh)=1.06449449, G(Eh)=-2686.31928804, Rel. G(kcal/mol)=5.72

15 SP_EE(Eh)=-2687.38299736, gcorr(Eh)=1.06396046, G(Eh)=-2686.31903690, Rel. G(kcal/mol)=5.88

16 SP_EE(Eh)=-2687.38276420, gcorr(Eh)=1.06437217, G(Eh)=-2686.31839203, Rel. G(kcal/mol)=6.28

17 SP_EE(Eh)=-2687.38078618, gcorr(Eh)=1.06274283, G(Eh)=-2686.31804335, Rel. G(kcal/mol)=6.50

18 SP_EE(Eh)=-2687.38026066, gcorr(Eh)=1.06312973, G(Eh)=-2686.31713093, Rel. G(kcal/mol)=7.08

19 SP_EE(Eh)=-2687.38022979, gcorr(Eh)=1.06328333, G(Eh)=-2686.31694646, Rel. G(kcal/mol)=7.19

20 SP_EE(Eh)=-2687.38057854, gcorr(Eh)=1.06384773, G(Eh)=-2686.31673081, Rel. G(kcal/mol)=7.33

21 SP_EE(Eh)=-2687.37873420, gcorr(Eh)=1.06290292, G(Eh)=-2686.31583128, Rel. G(kcal/mol)=7.89

2r9.xyz

1 SP_EE(Eh)=-2687.39294363, gcorr(Eh)=1.06275594, G(Eh)=-2686.33018769, Rel. G(kcal/mol)=0.00

2 SP_EE(Eh)=-2687.39008788, gcorr(Eh)=1.06168065, G(Eh)=-2686.32840723, Rel. G(kcal/mol)=1.12

3 SP_EE(Eh)=-2687.39269135, gcorr(Eh)=1.06444560, G(Eh)=-2686.32824575, Rel. G(kcal/mol)=1.22

4 SP_EE(Eh)=-2687.39024195, gcorr(Eh)=1.06374194, G(Eh)=-2686.32650001, Rel. G(kcal/mol)=2.31

5 SP_EE(Eh)=-2687.38740124, gcorr(Eh)=1.06354804, G(Eh)=-2686.32385320, Rel. G(kcal/mol)=3.97

6 SP_EE(Eh)=-2687.38437434, gcorr(Eh)=1.06218279, G(Eh)=-2686.32219155, Rel. G(kcal/mol)=5.02

7 SP_EE(Eh)=-2687.38361354, gcorr(Eh)=1.06161361, G(Eh)=-2686.32199993, Rel. G(kcal/mol)=5.14

8 SP_EE(Eh)=-2687.38412616, gcorr(Eh)=1.06265367, G(Eh)=-2686.32147249, Rel. G(kcal/mol)=5.47

9 SP_EE(Eh)=-2687.38605828, gcorr(Eh)=1.06476732, G(Eh)=-2686.32129096, Rel. G(kcal/mol)=5.58

10 SP_EE(Eh)=-2687.38340027, gcorr(Eh)=1.06275111, G(Eh)=-2686.32064916, Rel. G(kcal/mol)=5.99

11 SP_EE(Eh)=-2687.38299332, gcorr(Eh)=1.06322287, G(Eh)=-2686.31977045, Rel. G(kcal/mol)=6.54

12 SP_EE(Eh)=-2687.38381312, gcorr(Eh)=1.06469104, G(Eh)=-2686.31912208, Rel. G(kcal/mol)=6.94

13 SP_EE(Eh)=-2687.38379570, gcorr(Eh)=1.06481195, G(Eh)=-2686.31898375, Rel. G(kcal/mol)=7.03

14 SP_EE(Eh)=-2687.38176854, gcorr(Eh)=1.06361172, G(Eh)=-2686.31815682, Rel. G(kcal/mol)=7.55

15 SP_EE(Eh)=-2687.38230056, gcorr(Eh)=1.06420617, G(Eh)=-2686.31809439, Rel. G(kcal/mol)=7.59

16 SP_EE(Eh)=-2687.38218695, gcorr(Eh)=1.06473530, G(Eh)=-2686.31745165, Rel. G(kcal/mol)=7.99

3e4.xyz

1 SP_EE(Eh)=-2686.19835258, gcorr(Eh)=1.04190330, G(Eh)=-2685.15644928, Rel. G(kcal/mol)=0.00

2 SP_EE(Eh)=-2686.19805485, gcorr(Eh)=1.04280988, G(Eh)=-2685.15524497, Rel. G(kcal/mol)=0.76

3 SP_EE(Eh)=-2686.19859857, gcorr(Eh)=1.04380497, G(Eh)=-2685.15479360, Rel. G(kcal/mol)=1.04

4 SP_EE(Eh)=-2686.19761396, gcorr(Eh)=1.04302941, G(Eh)=-2685.15458455, Rel. G(kcal/mol)=1.17

5 SP_EE(Eh)=-2686.19839737, gcorr(Eh)=1.04406833, G(Eh)=-2685.15432904, Rel. G(kcal/mol)=1.33

6 SP_EE(Eh)=-2686.19879227, gcorr(Eh)=1.04480395, G(Eh)=-2685.15398832, Rel. G(kcal/mol)=1.54

7 SP_EE(Eh)=-2686.19618627, gcorr(Eh)=1.04222117, G(Eh)=-2685.15396510, Rel. G(kcal/mol)=1.56

8 SP_EE(Eh)=-2686.19703770, gcorr(Eh)=1.04415853, G(Eh)=-2685.15287917, Rel. G(kcal/mol)=2.24

9 SP_EE(Eh)=-2686.19564183, gcorr(Eh)=1.04328800, G(Eh)=-2685.15235383, Rel. G(kcal/mol)=2.57

10 SP_EE(Eh)=-2686.19532251, gcorr(Eh)=1.04310858, G(Eh)=-2685.15221393, Rel. G(kcal/mol)=2.66

11 SP_EE(Eh)=-2686.19458574, gcorr(Eh)=1.04255807, G(Eh)=-2685.15202767, Rel. G(kcal/mol)=2.77

12 SP_EE(Eh)=-2686.19429430, gcorr(Eh)=1.04227402, G(Eh)=-2685.15202028, Rel. G(kcal/mol)=2.78

13 SP_EE(Eh)=-2686.19476562, gcorr(Eh)=1.04285068, G(Eh)=-2685.15191494, Rel. G(kcal/mol)=2.85

14 SP_EE(Eh)=-2686.19616790, gcorr(Eh)=1.04429551, G(Eh)=-2685.15187239, Rel. G(kcal/mol)=2.87

15 SP_EE(Eh)=-2686.19568149, gcorr(Eh)=1.04399978, G(Eh)=-2685.15168171, Rel. G(kcal/mol)=2.99

16 SP_EE(Eh)=-2686.19687213, gcorr(Eh)=1.04541108, G(Eh)=-2685.15146105, Rel. G(kcal/mol)=3.13

17 SP_EE(Eh)=-2686.19273217, gcorr(Eh)=1.04139626, G(Eh)=-2685.15133591, Rel. G(kcal/mol)=3.21

18 SP_EE(Eh)=-2686.19336017, gcorr(Eh)=1.04247042, G(Eh)=-2685.15088975, Rel. G(kcal/mol)=3.49

19 SP_EE(Eh)=-2686.19342291, gcorr(Eh)=1.04254029, G(Eh)=-2685.15088262, Rel. G(kcal/mol)=3.49

20 SP_EE(Eh)=-2686.19270625, gcorr(Eh)=1.04216721, G(Eh)=-2685.15053904, Rel. G(kcal/mol)=3.71

21 SP_EE(Eh)=-2686.19204796, gcorr(Eh)=1.04156035, G(Eh)=-2685.15048761, Rel. G(kcal/mol)=3.74

22 SP_EE(Eh)=-2686.19290654, gcorr(Eh)=1.04267790, G(Eh)=-2685.15022864, Rel. G(kcal/mol)=3.90

23 SP_EE(Eh)=-2686.19270986, gcorr(Eh)=1.04249608, G(Eh)=-2685.15021378, Rel. G(kcal/mol)=3.91

24 SP_EE(Eh)=-2686.19336462, gcorr(Eh)=1.04371392, G(Eh)=-2685.14965070, Rel. G(kcal/mol)=4.27

25 SP_EE(Eh)=-2686.19211569, gcorr(Eh)=1.04253519, G(Eh)=-2685.14958050, Rel. G(kcal/mol)=4.31

26 SP_EE(Eh)=-2686.19224020, gcorr(Eh)=1.04274758, G(Eh)=-2685.14949262, Rel. G(kcal/mol)=4.37

27 SP_EE(Eh)=-2686.19266023, gcorr(Eh)=1.04365590, G(Eh)=-2685.14900433, Rel. G(kcal/mol)=4.67

28 SP_EE(Eh)=-2686.19312207, gcorr(Eh)=1.04416244, G(Eh)=-2685.14895963, Rel. G(kcal/mol)=4.70

29 SP_EE(Eh)=-2686.19137803, gcorr(Eh)=1.04263540, G(Eh)=-2685.14874263, Rel. G(kcal/mol)=4.84

30 SP_EE(Eh)=-2686.19206057, gcorr(Eh)=1.04362425, G(Eh)=-2685.14843632, Rel. G(kcal/mol)=5.03

31 SP_EE(Eh)=-2686.19235313, gcorr(Eh)=1.04392222, G(Eh)=-2685.14843091, Rel. G(kcal/mol)=5.03

32 SP_EE(Eh)=-2686.19277127, gcorr(Eh)=1.04489887, G(Eh)=-2685.14787240, Rel. G(kcal/mol)=5.38

33 SP_EE(Eh)=-2686.19128307, gcorr(Eh)=1.04344514, G(Eh)=-2685.14783793, Rel. G(kcal/mol)=5.40

34 SP_EE(Eh)=-2686.19145711, gcorr(Eh)=1.04423237, G(Eh)=-2685.14722474, Rel. G(kcal/mol)=5.79

35 SP_EE(Eh)=-2686.19017265, gcorr(Eh)=1.04329742, G(Eh)=-2685.14687523, Rel. G(kcal/mol)=6.01

36 SP_EE(Eh)=-2686.18746449, gcorr(Eh)=1.04218327, G(Eh)=-2685.14528122, Rel. G(kcal/mol)=7.01

37 SP_EE(Eh)=-2686.18936884, gcorr(Eh)=1.04537537, G(Eh)=-2685.14399347, Rel. G(kcal/mol)=7.82

3e6.xyz

1 SP_EE(Eh)=-3443.87031818, gcorr(Eh)=1.10976061, G(Eh)=-3442.76055757, Rel. G(kcal/mol)=0.00

2 SP_EE(Eh)=-3443.86523971, gcorr(Eh)=1.10803792, G(Eh)=-3442.75720179, Rel. G(kcal/mol)=2.11

3 SP_EE(Eh)=-3443.86517204, gcorr(Eh)=1.10948750, G(Eh)=-3442.75568454, Rel. G(kcal/mol)=3.06

4 SP_EE(Eh)=-3443.86409275, gcorr(Eh)=1.10931508, G(Eh)=-3442.75477767, Rel. G(kcal/mol)=3.63

5 SP_EE(Eh)=-3443.86301124, gcorr(Eh)=1.10835519, G(Eh)=-3442.75465605, Rel. G(kcal/mol)=3.70

6 SP_EE(Eh)=-3443.86321527, gcorr(Eh)=1.10870281, G(Eh)=-3442.75451246, Rel. G(kcal/mol)=3.79

7 SP_EE(Eh)=-3443.86399906, gcorr(Eh)=1.10951859, G(Eh)=-3442.75448047, Rel. G(kcal/mol)=3.81

8 SP_EE(Eh)=-3443.86172954, gcorr(Eh)=1.10831740, G(Eh)=-3442.75341214, Rel. G(kcal/mol)=4.48

9 SP_EE(Eh)=-3443.86093233, gcorr(Eh)=1.10757785, G(Eh)=-3442.75335448, Rel. G(kcal/mol)=4.52

10 SP_EE(Eh)=-3443.86240509, gcorr(Eh)=1.10954526, G(Eh)=-3442.75285983, Rel. G(kcal/mol)=4.83

11 SP_EE(Eh)=-3443.86259463, gcorr(Eh)=1.10976058, G(Eh)=-3442.75283405, Rel. G(kcal/mol)=4.85

12 SP_EE(Eh)=-3443.86060330, gcorr(Eh)=1.10845654, G(Eh)=-3442.75214676, Rel. G(kcal/mol)=5.28

13 SP_EE(Eh)=-3443.86201159, gcorr(Eh)=1.11004533, G(Eh)=-3442.75196626, Rel. G(kcal/mol)=5.39

14 SP_EE(Eh)=-3443.86031518, gcorr(Eh)=1.10870368, G(Eh)=-3442.75161150, Rel. G(kcal/mol)=5.61

15 SP_EE(Eh)=-3443.85978861, gcorr(Eh)=1.10843391, G(Eh)=-3442.75135470, Rel. G(kcal/mol)=5.77

16 SP_EE(Eh)=-3443.86033769, gcorr(Eh)=1.10931683, G(Eh)=-3442.75102086, Rel. G(kcal/mol)=5.98

17 SP_EE(Eh)=-3443.86030776, gcorr(Eh)=1.10947535, G(Eh)=-3442.75083241, Rel. G(kcal/mol)=6.10

18 SP_EE(Eh)=-3443.85891045, gcorr(Eh)=1.10856201, G(Eh)=-3442.75034844, Rel. G(kcal/mol)=6.41

3r4.xyz

1 SP_EE(Eh)=-2686.19471817, gcorr(Eh)=1.04262020, G(Eh)=-2685.15209797, Rel. G(kcal/mol)=0.00

2 SP_EE(Eh)=-2686.19629279, gcorr(Eh)=1.04424229, G(Eh)=-2685.15205050, Rel. G(kcal/mol)=0.03

3 SP_EE(Eh)=-2686.19411241, gcorr(Eh)=1.04221155, G(Eh)=-2685.15190086, Rel. G(kcal/mol)=0.12

4 SP_EE(Eh)=-2686.19556570, gcorr(Eh)=1.04375116, G(Eh)=-2685.15181454, Rel. G(kcal/mol)=0.18

5 SP_EE(Eh)=-2686.19274878, gcorr(Eh)=1.04103717, G(Eh)=-2685.15171161, Rel. G(kcal/mol)=0.24

6 SP_EE(Eh)=-2686.19611189, gcorr(Eh)=1.04457362, G(Eh)=-2685.15153827, Rel. G(kcal/mol)=0.35

7 SP_EE(Eh)=-2686.19506904, gcorr(Eh)=1.04359930, G(Eh)=-2685.15146974, Rel. G(kcal/mol)=0.39

8 SP_EE(Eh)=-2686.19569550, gcorr(Eh)=1.04426553, G(Eh)=-2685.15142997, Rel. G(kcal/mol)=0.42

9 SP_EE(Eh)=-2686.19457238, gcorr(Eh)=1.04370028, G(Eh)=-2685.15087210, Rel. G(kcal/mol)=0.77

10 SP_EE(Eh)=-2686.19453091, gcorr(Eh)=1.04402612, G(Eh)=-2685.15050479, Rel. G(kcal/mol)=1.00

11 SP_EE(Eh)=-2686.19356029, gcorr(Eh)=1.04310835, G(Eh)=-2685.15045194, Rel. G(kcal/mol)=1.03

12 SP_EE(Eh)=-2686.19555242, gcorr(Eh)=1.04525543, G(Eh)=-2685.15029699, Rel. G(kcal/mol)=1.13

13 SP_EE(Eh)=-2686.19613771, gcorr(Eh)=1.04584837, G(Eh)=-2685.15028934, Rel. G(kcal/mol)=1.13

14 SP_EE(Eh)=-2686.19321998, gcorr(Eh)=1.04342063, G(Eh)=-2685.14979935, Rel. G(kcal/mol)=1.44

15 SP_EE(Eh)=-2686.19237252, gcorr(Eh)=1.04348760, G(Eh)=-2685.14888492, Rel. G(kcal/mol)=2.02

16 SP_EE(Eh)=-2686.19191378, gcorr(Eh)=1.04311038, G(Eh)=-2685.14880340, Rel. G(kcal/mol)=2.07

17 SP_EE(Eh)=-2686.19379909, gcorr(Eh)=1.04502077, G(Eh)=-2685.14877832, Rel. G(kcal/mol)=2.08

18 SP_EE(Eh)=-2686.19048971, gcorr(Eh)=1.04455258, G(Eh)=-2685.14593713, Rel. G(kcal/mol)=3.87

3r6.xyz

1 SP_EE(Eh)=-3443.86899495, gcorr(Eh)=1.10911145, G(Eh)=-3442.75988350, Rel. G(kcal/mol)=0.00

2 SP_EE(Eh)=-3443.86854131, gcorr(Eh)=1.10876591, G(Eh)=-3442.75977540, Rel. G(kcal/mol)=0.07

3 SP_EE(Eh)=-3443.86668521, gcorr(Eh)=1.10719757, G(Eh)=-3442.75948764, Rel. G(kcal/mol)=0.25

4 SP_EE(Eh)=-3443.86706451, gcorr(Eh)=1.10797958, G(Eh)=-3442.75908493, Rel. G(kcal/mol)=0.50

5 SP_EE(Eh)=-3443.86639856, gcorr(Eh)=1.10816258, G(Eh)=-3442.75823598, Rel. G(kcal/mol)=1.03

6 SP_EE(Eh)=-3443.86642694, gcorr(Eh)=1.10855442, G(Eh)=-3442.75787252, Rel. G(kcal/mol)=1.26

7 SP_EE(Eh)=-3443.86576910, gcorr(Eh)=1.10870087, G(Eh)=-3442.75706823, Rel. G(kcal/mol)=1.77

8 SP_EE(Eh)=-3443.86576645, gcorr(Eh)=1.10924011, G(Eh)=-3442.75652634, Rel. G(kcal/mol)=2.11

9 SP_EE(Eh)=-3443.86296404, gcorr(Eh)=1.10920921, G(Eh)=-3442.75375483, Rel. G(kcal/mol)=3.85

10 SP_EE(Eh)=-3443.86140287, gcorr(Eh)=1.10780314, G(Eh)=-3442.75359973, Rel. G(kcal/mol)=3.94

11 SP_EE(Eh)=-3443.86197905, gcorr(Eh)=1.10869105, G(Eh)=-3442.75328800, Rel. G(kcal/mol)=4.14

12 SP_EE(Eh)=-3443.86220849, gcorr(Eh)=1.10931525, G(Eh)=-3442.75289324, Rel. G(kcal/mol)=4.39

13 SP_EE(Eh)=-3443.86087121, gcorr(Eh)=1.10849711, G(Eh)=-3442.75237410, Rel. G(kcal/mol)=4.71

14 SP_EE(Eh)=-3443.86246339, gcorr(Eh)=1.11028083, G(Eh)=-3442.75218256, Rel. G(kcal/mol)=4.83

15 SP_EE(Eh)=-3443.85986273, gcorr(Eh)=1.10825280, G(Eh)=-3442.75160993, Rel. G(kcal/mol)=5.19

16 SP_EE(Eh)=-3443.85935658, gcorr(Eh)=1.10898287, G(Eh)=-3442.75037371, Rel. G(kcal/mol)=5.97

4e10.xyz

1 SP_EE(Eh)=-2954.18220082, gcorr(Eh)=1.09992111, G(Eh)=-2953.08227971, Rel. G(kcal/mol)=0.00

2 SP_EE(Eh)=-2954.18003606, gcorr(Eh)=1.09915044, G(Eh)=-2953.08088562, Rel. G(kcal/mol)=0.87

3 SP_EE(Eh)=-2954.18099252, gcorr(Eh)=1.10081684, G(Eh)=-2953.08017568, Rel. G(kcal/mol)=1.32

4 SP_EE(Eh)=-2954.17899928, gcorr(Eh)=1.10014595, G(Eh)=-2953.07885333, Rel. G(kcal/mol)=2.15

5 SP_EE(Eh)=-2954.17804720, gcorr(Eh)=1.09920094, G(Eh)=-2953.07884626, Rel. G(kcal/mol)=2.15

6 SP_EE(Eh)=-2954.18006032, gcorr(Eh)=1.10163241, G(Eh)=-2953.07842791, Rel. G(kcal/mol)=2.42

7 SP_EE(Eh)=-2954.17766143, gcorr(Eh)=1.09985552, G(Eh)=-2953.07780591, Rel. G(kcal/mol)=2.81

8 SP_EE(Eh)=-2954.17960351, gcorr(Eh)=1.10182203, G(Eh)=-2953.07778148, Rel. G(kcal/mol)=2.82

9 SP_EE(Eh)=-2954.17852868, gcorr(Eh)=1.10101753, G(Eh)=-2953.07751115, Rel. G(kcal/mol)=2.99

10 SP_EE(Eh)=-2954.17906269, gcorr(Eh)=1.10162739, G(Eh)=-2953.07743530, Rel. G(kcal/mol)=3.04

11 SP_EE(Eh)=-2954.17835185, gcorr(Eh)=1.10111614, G(Eh)=-2953.07723571, Rel. G(kcal/mol)=3.17

12 SP_EE(Eh)=-2954.17858419, gcorr(Eh)=1.10171938, G(Eh)=-2953.07686481, Rel. G(kcal/mol)=3.40

13 SP_EE(Eh)=-2954.17542863, gcorr(Eh)=1.09867073, G(Eh)=-2953.07675790, Rel. G(kcal/mol)=3.46

14 SP_EE(Eh)=-2954.17688082, gcorr(Eh)=1.10027635, G(Eh)=-2953.07660447, Rel. G(kcal/mol)=3.56

15 SP_EE(Eh)=-2954.17694001, gcorr(Eh)=1.10042284, G(Eh)=-2953.07651717, Rel. G(kcal/mol)=3.62

16 SP_EE(Eh)=-2954.17842947, gcorr(Eh)=1.10198628, G(Eh)=-2953.07644319, Rel. G(kcal/mol)=3.66

17 SP_EE(Eh)=-2954.17574128, gcorr(Eh)=1.09944225, G(Eh)=-2953.07629903, Rel. G(kcal/mol)=3.75

18 SP_EE(Eh)=-2954.17694764, gcorr(Eh)=1.10066592, G(Eh)=-2953.07628172, Rel. G(kcal/mol)=3.76

19 SP_EE(Eh)=-2954.17728184, gcorr(Eh)=1.10103452, G(Eh)=-2953.07624732, Rel. G(kcal/mol)=3.79

20 SP_EE(Eh)=-2954.17609353, gcorr(Eh)=1.10007498, G(Eh)=-2953.07601855, Rel. G(kcal/mol)=3.93

21 SP_EE(Eh)=-2954.17581979, gcorr(Eh)=1.09984950, G(Eh)=-2953.07597029, Rel. G(kcal/mol)=3.96

22 SP_EE(Eh)=-2954.17747329, gcorr(Eh)=1.10160615, G(Eh)=-2953.07586714, Rel. G(kcal/mol)=4.02

23 SP_EE(Eh)=-2954.17599801, gcorr(Eh)=1.10013844, G(Eh)=-2953.07585957, Rel. G(kcal/mol)=4.03

24 SP_EE(Eh)=-2954.17732047, gcorr(Eh)=1.10152416, G(Eh)=-2953.07579631, Rel. G(kcal/mol)=4.07

25 SP_EE(Eh)=-2954.17571262, gcorr(Eh)=1.10006658, G(Eh)=-2953.07564604, Rel. G(kcal/mol)=4.16

26 SP_EE(Eh)=-2954.17518312, gcorr(Eh)=1.09956313, G(Eh)=-2953.07561999, Rel. G(kcal/mol)=4.18

27 SP_EE(Eh)=-2954.17636754, gcorr(Eh)=1.10089625, G(Eh)=-2953.07547129, Rel. G(kcal/mol)=4.27

28 SP_EE(Eh)=-2954.17602934, gcorr(Eh)=1.10082133, G(Eh)=-2953.07520801, Rel. G(kcal/mol)=4.44

29 SP_EE(Eh)=-2954.17754626, gcorr(Eh)=1.10234920, G(Eh)=-2953.07519706, Rel. G(kcal/mol)=4.44

30 SP_EE(Eh)=-2954.17532178, gcorr(Eh)=1.10054555, G(Eh)=-2953.07477623, Rel. G(kcal/mol)=4.71

31 SP_EE(Eh)=-2954.17500928, gcorr(Eh)=1.10027377, G(Eh)=-2953.07473551, Rel. G(kcal/mol)=4.73

32 SP_EE(Eh)=-2954.17600709, gcorr(Eh)=1.10132800, G(Eh)=-2953.07467909, Rel. G(kcal/mol)=4.77

33 SP_EE(Eh)=-2954.17499262, gcorr(Eh)=1.10037634, G(Eh)=-2953.07461628, Rel. G(kcal/mol)=4.81

34 SP_EE(Eh)=-2954.17582610, gcorr(Eh)=1.10148908, G(Eh)=-2953.07433702, Rel. G(kcal/mol)=4.98

35 SP_EE(Eh)=-2954.17429747, gcorr(Eh)=1.09999529, G(Eh)=-2953.07430218, Rel. G(kcal/mol)=5.01

36 SP_EE(Eh)=-2954.17424612, gcorr(Eh)=1.10042504, G(Eh)=-2953.07382108, Rel. G(kcal/mol)=5.31

37 SP_EE(Eh)=-2954.17463936, gcorr(Eh)=1.10085862, G(Eh)=-2953.07378074, Rel. G(kcal/mol)=5.33

38 SP_EE(Eh)=-2954.17319377, gcorr(Eh)=1.09962895, G(Eh)=-2953.07356482, Rel. G(kcal/mol)=5.47

39 SP_EE(Eh)=-2954.17475219, gcorr(Eh)=1.10118815, G(Eh)=-2953.07356404, Rel. G(kcal/mol)=5.47

40 SP_EE(Eh)=-2954.17384344, gcorr(Eh)=1.10033820, G(Eh)=-2953.07350524, Rel. G(kcal/mol)=5.51

41 SP_EE(Eh)=-2954.17516900, gcorr(Eh)=1.10177592, G(Eh)=-2953.07339308, Rel. G(kcal/mol)=5.58

42 SP_EE(Eh)=-2954.17431304, gcorr(Eh)=1.10094652, G(Eh)=-2953.07336652, Rel. G(kcal/mol)=5.59

43 SP_EE(Eh)=-2954.17338010, gcorr(Eh)=1.10002860, G(Eh)=-2953.07335150, Rel. G(kcal/mol)=5.60

44 SP_EE(Eh)=-2954.17455040, gcorr(Eh)=1.10125910, G(Eh)=-2953.07329130, Rel. G(kcal/mol)=5.64

45 SP_EE(Eh)=-2954.17381338, gcorr(Eh)=1.10053655, G(Eh)=-2953.07327683, Rel. G(kcal/mol)=5.65

46 SP_EE(Eh)=-2954.17336346, gcorr(Eh)=1.10015789, G(Eh)=-2953.07320557, Rel. G(kcal/mol)=5.69

47 SP_EE(Eh)=-2954.17353547, gcorr(Eh)=1.10046058, G(Eh)=-2953.07307489, Rel. G(kcal/mol)=5.78

48 SP_EE(Eh)=-2954.17407403, gcorr(Eh)=1.10104871, G(Eh)=-2953.07302532, Rel. G(kcal/mol)=5.81

49 SP_EE(Eh)=-2954.17374807, gcorr(Eh)=1.10079394, G(Eh)=-2953.07295413, Rel. G(kcal/mol)=5.85

50 SP_EE(Eh)=-2954.17438632, gcorr(Eh)=1.10147326, G(Eh)=-2953.07291306, Rel. G(kcal/mol)=5.88

51 SP_EE(Eh)=-2954.17399479, gcorr(Eh)=1.10119441, G(Eh)=-2953.07280038, Rel. G(kcal/mol)=5.95

52 SP_EE(Eh)=-2954.17465782, gcorr(Eh)=1.10188330, G(Eh)=-2953.07277452, Rel. G(kcal/mol)=5.96

53 SP_EE(Eh)=-2954.17453989, gcorr(Eh)=1.10180895, G(Eh)=-2953.07273094, Rel. G(kcal/mol)=5.99

54 SP_EE(Eh)=-2954.17296991, gcorr(Eh)=1.10025235, G(Eh)=-2953.07271756, Rel. G(kcal/mol)=6.00

55 SP_EE(Eh)=-2954.17407616, gcorr(Eh)=1.10143341, G(Eh)=-2953.07264275, Rel. G(kcal/mol)=6.05

56 SP_EE(Eh)=-2954.17412503, gcorr(Eh)=1.10166608, G(Eh)=-2953.07245895, Rel. G(kcal/mol)=6.16

57 SP_EE(Eh)=-2954.17406753, gcorr(Eh)=1.10165942, G(Eh)=-2953.07240811, Rel. G(kcal/mol)=6.19

58 SP_EE(Eh)=-2954.17129531, gcorr(Eh)=1.09903441, G(Eh)=-2953.07226090, Rel. G(kcal/mol)=6.29

59 SP_EE(Eh)=-2954.17318819, gcorr(Eh)=1.10100733, G(Eh)=-2953.07218086, Rel. G(kcal/mol)=6.34

60 SP_EE(Eh)=-2954.17221364, gcorr(Eh)=1.10010406, G(Eh)=-2953.07210958, Rel. G(kcal/mol)=6.38

61 SP_EE(Eh)=-2954.17300413, gcorr(Eh)=1.10104662, G(Eh)=-2953.07195751, Rel. G(kcal/mol)=6.48

62 SP_EE(Eh)=-2954.17182939, gcorr(Eh)=1.09997476, G(Eh)=-2953.07185463, Rel. G(kcal/mol)=6.54

63 SP_EE(Eh)=-2954.17312016, gcorr(Eh)=1.10131021, G(Eh)=-2953.07180995, Rel. G(kcal/mol)=6.57

64 SP_EE(Eh)=-2954.17232780, gcorr(Eh)=1.10059540, G(Eh)=-2953.07173240, Rel. G(kcal/mol)=6.62

65 SP_EE(Eh)=-2954.17335032, gcorr(Eh)=1.10170487, G(Eh)=-2953.07164545, Rel. G(kcal/mol)=6.67

66 SP_EE(Eh)=-2954.17218036, gcorr(Eh)=1.10057085, G(Eh)=-2953.07160951, Rel. G(kcal/mol)=6.70

67 SP_EE(Eh)=-2954.17249111, gcorr(Eh)=1.10117114, G(Eh)=-2953.07131997, Rel. G(kcal/mol)=6.88

68 SP_EE(Eh)=-2954.17120593, gcorr(Eh)=1.09995481, G(Eh)=-2953.07125112, Rel. G(kcal/mol)=6.92

69 SP_EE(Eh)=-2954.17048920, gcorr(Eh)=1.09945504, G(Eh)=-2953.07103416, Rel. G(kcal/mol)=7.06

70 SP_EE(Eh)=-2954.17274380, gcorr(Eh)=1.10176985, G(Eh)=-2953.07097395, Rel. G(kcal/mol)=7.09

71 SP_EE(Eh)=-2954.17238016, gcorr(Eh)=1.10142765, G(Eh)=-2953.07095251, Rel. G(kcal/mol)=7.11

72 SP_EE(Eh)=-2954.17177330, gcorr(Eh)=1.10082799, G(Eh)=-2953.07094531, Rel. G(kcal/mol)=7.11

73 SP_EE(Eh)=-2954.17226197, gcorr(Eh)=1.10149605, G(Eh)=-2953.07076592, Rel. G(kcal/mol)=7.23

74 SP_EE(Eh)=-2954.17270526, gcorr(Eh)=1.10205862, G(Eh)=-2953.07064664, Rel. G(kcal/mol)=7.30

75 SP_EE(Eh)=-2954.17073575, gcorr(Eh)=1.10015648, G(Eh)=-2953.07057927, Rel. G(kcal/mol)=7.34

76 SP_EE(Eh)=-2954.17206743, gcorr(Eh)=1.10174607, G(Eh)=-2953.07032136, Rel. G(kcal/mol)=7.50

77 SP_EE(Eh)=-2954.17072786, gcorr(Eh)=1.10042252, G(Eh)=-2953.07030534, Rel. G(kcal/mol)=7.51

78 SP_EE(Eh)=-2954.17171782, gcorr(Eh)=1.10176634, G(Eh)=-2953.06995148, Rel. G(kcal/mol)=7.74

79 SP_EE(Eh)=-2954.17022008, gcorr(Eh)=1.10046513, G(Eh)=-2953.06975495, Rel. G(kcal/mol)=7.86

80 SP_EE(Eh)=-2954.17046162, gcorr(Eh)=1.10083792, G(Eh)=-2953.06962370, Rel. G(kcal/mol)=7.94

4e11.xyz

1 SP_EE(Eh)=-2570.68050781, gcorr(Eh)=1.00023161, G(Eh)=-2569.68027620, Rel. G(kcal/mol)=0.00

2 SP_EE(Eh)=-2570.67956140, gcorr(Eh)=0.99957047, G(Eh)=-2569.67999093, Rel. G(kcal/mol)=0.18

3 SP_EE(Eh)=-2570.67946207, gcorr(Eh)=1.00071296, G(Eh)=-2569.67874911, Rel. G(kcal/mol)=0.96

4 SP_EE(Eh)=-2570.67884983, gcorr(Eh)=1.00053021, G(Eh)=-2569.67831962, Rel. G(kcal/mol)=1.23

5 SP_EE(Eh)=-2570.67838450, gcorr(Eh)=1.00034363, G(Eh)=-2569.67804087, Rel. G(kcal/mol)=1.40

6 SP_EE(Eh)=-2570.67744066, gcorr(Eh)=0.99962971, G(Eh)=-2569.67781095, Rel. G(kcal/mol)=1.55

7 SP_EE(Eh)=-2570.67608529, gcorr(Eh)=0.99868406, G(Eh)=-2569.67740123, Rel. G(kcal/mol)=1.80

8 SP_EE(Eh)=-2570.67740911, gcorr(Eh)=1.00004982, G(Eh)=-2569.67735929, Rel. G(kcal/mol)=1.83

9 SP_EE(Eh)=-2570.67581067, gcorr(Eh)=0.99854352, G(Eh)=-2569.67726715, Rel. G(kcal/mol)=1.89

10 SP_EE(Eh)=-2570.67620221, gcorr(Eh)=0.99960287, G(Eh)=-2569.67659934, Rel. G(kcal/mol)=2.31

11 SP_EE(Eh)=-2570.67627803, gcorr(Eh)=1.00012709, G(Eh)=-2569.67615094, Rel. G(kcal/mol)=2.59

12 SP_EE(Eh)=-2570.67753631, gcorr(Eh)=1.00164432, G(Eh)=-2569.67589199, Rel. G(kcal/mol)=2.75

13 SP_EE(Eh)=-2570.67650385, gcorr(Eh)=1.00078318, G(Eh)=-2569.67572067, Rel. G(kcal/mol)=2.86

14 SP_EE(Eh)=-2570.67702066, gcorr(Eh)=1.00183990, G(Eh)=-2569.67518076, Rel. G(kcal/mol)=3.20

15 SP_EE(Eh)=-2570.67320687, gcorr(Eh)=0.99847774, G(Eh)=-2569.67472913, Rel. G(kcal/mol)=3.48

16 SP_EE(Eh)=-2570.67479651, gcorr(Eh)=1.00039556, G(Eh)=-2569.67440095, Rel. G(kcal/mol)=3.69

17 SP_EE(Eh)=-2570.67499144, gcorr(Eh)=1.00075190, G(Eh)=-2569.67423954, Rel. G(kcal/mol)=3.79

18 SP_EE(Eh)=-2570.67427585, gcorr(Eh)=1.00006685, G(Eh)=-2569.67420900, Rel. G(kcal/mol)=3.81

19 SP_EE(Eh)=-2570.67410494, gcorr(Eh)=0.99995392, G(Eh)=-2569.67415102, Rel. G(kcal/mol)=3.84

20 SP_EE(Eh)=-2570.67492822, gcorr(Eh)=1.00114847, G(Eh)=-2569.67377975, Rel. G(kcal/mol)=4.08

21 SP_EE(Eh)=-2570.67399908, gcorr(Eh)=1.00165645, G(Eh)=-2569.67234263, Rel. G(kcal/mol)=4.98

22 SP_EE(Eh)=-2570.67314127, gcorr(Eh)=1.00162451, G(Eh)=-2569.67151676, Rel. G(kcal/mol)=5.50

23 SP_EE(Eh)=-2570.66966940, gcorr(Eh)=0.99962301, G(Eh)=-2569.67004639, Rel. G(kcal/mol)=6.42

4e4.xyz

1 SP_EE(Eh)=-2686.20062702, gcorr(Eh)=1.04168500, G(Eh)=-2685.15894202, Rel. G(kcal/mol)=0.00

2 SP_EE(Eh)=-2686.19793574, gcorr(Eh)=1.04103900, G(Eh)=-2685.15689674, Rel. G(kcal/mol)=1.28

3 SP_EE(Eh)=-2686.19743447, gcorr(Eh)=1.04248889, G(Eh)=-2685.15494558, Rel. G(kcal/mol)=2.51

4 SP_EE(Eh)=-2686.19832541, gcorr(Eh)=1.04431891, G(Eh)=-2685.15400650, Rel. G(kcal/mol)=3.10

5 SP_EE(Eh)=-2686.19734191, gcorr(Eh)=1.04461531, G(Eh)=-2685.15272660, Rel. G(kcal/mol)=3.90

6 SP_EE(Eh)=-2686.19513877, gcorr(Eh)=1.04267468, G(Eh)=-2685.15246409, Rel. G(kcal/mol)=4.06

7 SP_EE(Eh)=-2686.19584819, gcorr(Eh)=1.04358478, G(Eh)=-2685.15226341, Rel. G(kcal/mol)=4.19

8 SP_EE(Eh)=-2686.19385750, gcorr(Eh)=1.04159947, G(Eh)=-2685.15225803, Rel. G(kcal/mol)=4.19

9 SP_EE(Eh)=-2686.19619934, gcorr(Eh)=1.04394273, G(Eh)=-2685.15225661, Rel. G(kcal/mol)=4.20

10 SP_EE(Eh)=-2686.19581849, gcorr(Eh)=1.04456082, G(Eh)=-2685.15125767, Rel. G(kcal/mol)=4.82

11 SP_EE(Eh)=-2686.19451804, gcorr(Eh)=1.04349150, G(Eh)=-2685.15102654, Rel. G(kcal/mol)=4.97

12 SP_EE(Eh)=-2686.19362602, gcorr(Eh)=1.04277829, G(Eh)=-2685.15084773, Rel. G(kcal/mol)=5.08

13 SP_EE(Eh)=-2686.19343632, gcorr(Eh)=1.04278920, G(Eh)=-2685.15064712, Rel. G(kcal/mol)=5.21

14 SP_EE(Eh)=-2686.19437104, gcorr(Eh)=1.04398190, G(Eh)=-2685.15038914, Rel. G(kcal/mol)=5.37

15 SP_EE(Eh)=-2686.19371239, gcorr(Eh)=1.04382074, G(Eh)=-2685.14989165, Rel. G(kcal/mol)=5.68

16 SP_EE(Eh)=-2686.19411127, gcorr(Eh)=1.04429562, G(Eh)=-2685.14981565, Rel. G(kcal/mol)=5.73

17 SP_EE(Eh)=-2686.19336141, gcorr(Eh)=1.04391431, G(Eh)=-2685.14944710, Rel. G(kcal/mol)=5.96

18 SP_EE(Eh)=-2686.19462102, gcorr(Eh)=1.04518418, G(Eh)=-2685.14943684, Rel. G(kcal/mol)=5.96

19 SP_EE(Eh)=-2686.19294501, gcorr(Eh)=1.04354214, G(Eh)=-2685.14940287, Rel. G(kcal/mol)=5.99

20 SP_EE(Eh)=-2686.19113860, gcorr(Eh)=1.04214245, G(Eh)=-2685.14899615, Rel. G(kcal/mol)=6.24

21 SP_EE(Eh)=-2686.19131774, gcorr(Eh)=1.04275467, G(Eh)=-2685.14856307, Rel. G(kcal/mol)=6.51
22 SP_EE(Eh)=-2686.19045138, gcorr(Eh)=1.04244500, G(Eh)=-2685.14800638, Rel. G(kcal/mol)=6.86
23 SP_EE(Eh)=-2686.19274295, gcorr(Eh)=1.04476566, G(Eh)=-2685.14797729, Rel. G(kcal/mol)=6.88
24 SP_EE(Eh)=-2686.19112836, gcorr(Eh)=1.04413344, G(Eh)=-2685.14699492, Rel. G(kcal/mol)=7.50
25 SP_EE(Eh)=-2686.19109288, gcorr(Eh)=1.04443927, G(Eh)=-2685.14665361, Rel. G(kcal/mol)=7.71
26 SP_EE(Eh)=-2686.19063589, gcorr(Eh)=1.04430857, G(Eh)=-2685.14632732, Rel. G(kcal/mol)=7.92
27 SP_EE(Eh)=-2686.18928102, gcorr(Eh)=1.04310467, G(Eh)=-2685.14617635, Rel. G(kcal/mol)=8.01
28 SP_EE(Eh)=-2686.19071571, gcorr(Eh)=1.04454281, G(Eh)=-2685.14617290, Rel. G(kcal/mol)=8.01
29 SP_EE(Eh)=-2686.19066571, gcorr(Eh)=1.04455125, G(Eh)=-2685.14611446, Rel. G(kcal/mol)=8.05
30 SP_EE(Eh)=-2686.18887340, gcorr(Eh)=1.04297678, G(Eh)=-2685.14589662, Rel. G(kcal/mol)=8.19
31 SP_EE(Eh)=-2686.18979606, gcorr(Eh)=1.04406056, G(Eh)=-2685.14573550, Rel. G(kcal/mol)=8.29
32 SP_EE(Eh)=-2686.18909874, gcorr(Eh)=1.04406538, G(Eh)=-2685.14503336, Rel. G(kcal/mol)=8.73
33 SP_EE(Eh)=-2686.18920341, gcorr(Eh)=1.04429006, G(Eh)=-2685.14491335, Rel. G(kcal/mol)=8.80
34 SP_EE(Eh)=-2686.18825338, gcorr(Eh)=1.04386634, G(Eh)=-2685.14438704, Rel. G(kcal/mol)=9.13
35 SP_EE(Eh)=-2686.18824717, gcorr(Eh)=1.04435346, G(Eh)=-2685.14389371, Rel. G(kcal/mol)=9.44
36 SP_EE(Eh)=-2686.18731686, gcorr(Eh)=1.04376610, G(Eh)=-2685.14355076, Rel. G(kcal/mol)=9.66

4e9.xyz

1 SP_EE(Eh)=-2648.10200750, gcorr(Eh)=1.03569891, G(Eh)=-2647.06630859, Rel. G(kcal/mol)=0.00
2 SP_EE(Eh)=-2648.10058275, gcorr(Eh)=1.03615489, G(Eh)=-2647.06442786, Rel. G(kcal/mol)=1.18
3 SP_EE(Eh)=-2648.10148198, gcorr(Eh)=1.03709329, G(Eh)=-2647.06438869, Rel. G(kcal/mol)=1.20
4 SP_EE(Eh)=-2648.10088819, gcorr(Eh)=1.03701333, G(Eh)=-2647.06387486, Rel. G(kcal/mol)=1.53
5 SP_EE(Eh)=-2648.09998911, gcorr(Eh)=1.03684509, G(Eh)=-2647.06314402, Rel. G(kcal/mol)=1.99
6 SP_EE(Eh)=-2648.09925836, gcorr(Eh)=1.03776089, G(Eh)=-2647.06149747, Rel. G(kcal/mol)=3.02
7 SP_EE(Eh)=-2648.09740221, gcorr(Eh)=1.03821129, G(Eh)=-2647.05919092, Rel. G(kcal/mol)=4.47
8 SP_EE(Eh)=-2648.09452054, gcorr(Eh)=1.03547693, G(Eh)=-2647.05904361, Rel. G(kcal/mol)=4.56
9 SP_EE(Eh)=-2648.09558036, gcorr(Eh)=1.03654896, G(Eh)=-2647.05903140, Rel. G(kcal/mol)=4.57
10 SP_EE(Eh)=-2648.09469780, gcorr(Eh)=1.03590691, G(Eh)=-2647.05879089, Rel. G(kcal/mol)=4.72
11 SP_EE(Eh)=-2648.09501008, gcorr(Eh)=1.03673057, G(Eh)=-2647.05827951, Rel. G(kcal/mol)=5.04
12 SP_EE(Eh)=-2648.09453047, gcorr(Eh)=1.03644013, G(Eh)=-2647.05809034, Rel. G(kcal/mol)=5.16
13 SP_EE(Eh)=-2648.09417141, gcorr(Eh)=1.03628580, G(Eh)=-2647.05788561, Rel. G(kcal/mol)=5.29
14 SP_EE(Eh)=-2648.09371168, gcorr(Eh)=1.03687945, G(Eh)=-2647.05683223, Rel. G(kcal/mol)=5.95
15 SP_EE(Eh)=-2648.09339585, gcorr(Eh)=1.03672529, G(Eh)=-2647.05667056, Rel. G(kcal/mol)=6.05
16 SP_EE(Eh)=-2648.09310091, gcorr(Eh)=1.03644305, G(Eh)=-2647.05665786, Rel. G(kcal/mol)=6.06
17 SP_EE(Eh)=-2648.09428741, gcorr(Eh)=1.03829856, G(Eh)=-2647.05598885, Rel. G(kcal/mol)=6.48
18 SP_EE(Eh)=-2648.09140105, gcorr(Eh)=1.03547532, G(Eh)=-2647.05592573, Rel. G(kcal/mol)=6.52
19 SP_EE(Eh)=-2648.09090396, gcorr(Eh)=1.03623655, G(Eh)=-2647.05466741, Rel. G(kcal/mol)=7.30
20 SP_EE(Eh)=-2648.09269563, gcorr(Eh)=1.03852523, G(Eh)=-2647.05417040, Rel. G(kcal/mol)=7.62
21 SP_EE(Eh)=-2648.08787832, gcorr(Eh)=1.03444422, G(Eh)=-2647.05343410, Rel. G(kcal/mol)=8.08

22 SP_EE(Eh)=-2648.08997520, gcorr(Eh)=1.03659334, G(Eh)=-2647.05338186, Rel. G(kcal/mol)=8.11

23 SP_EE(Eh)=-2648.08756885, gcorr(Eh)=1.03475348, G(Eh)=-2647.05281537, Rel. G(kcal/mol)=8.47

24 SP_EE(Eh)=-2648.08964196, gcorr(Eh)=1.03722407, G(Eh)=-2647.05241789, Rel. G(kcal/mol)=8.72

25 SP_EE(Eh)=-2648.08927482, gcorr(Eh)=1.03864209, G(Eh)=-2647.05063273, Rel. G(kcal/mol)=9.84

4r10.xyz

1 SP_EE(Eh)=-2954.18127852, gcorr(Eh)=1.10027530, G(Eh)=-2953.08100322, Rel. G(kcal/mol)=0.00

2 SP_EE(Eh)=-2954.17982277, gcorr(Eh)=1.10053991, G(Eh)=-2953.07928286, Rel. G(kcal/mol)=1.08

3 SP_EE(Eh)=-2954.17953505, gcorr(Eh)=1.10050211, G(Eh)=-2953.07903294, Rel. G(kcal/mol)=1.24

4 SP_EE(Eh)=-2954.17922776, gcorr(Eh)=1.10124408, G(Eh)=-2953.07798368, Rel. G(kcal/mol)=1.89

5 SP_EE(Eh)=-2954.17835182, gcorr(Eh)=1.10057800, G(Eh)=-2953.07777382, Rel. G(kcal/mol)=2.03

6 SP_EE(Eh)=-2954.17764698, gcorr(Eh)=1.10017900, G(Eh)=-2953.07746798, Rel. G(kcal/mol)=2.22

7 SP_EE(Eh)=-2954.17827025, gcorr(Eh)=1.10089424, G(Eh)=-2953.07737601, Rel. G(kcal/mol)=2.28

8 SP_EE(Eh)=-2954.17702171, gcorr(Eh)=1.09965938, G(Eh)=-2953.07736233, Rel. G(kcal/mol)=2.28

9 SP_EE(Eh)=-2954.17827208, gcorr(Eh)=1.10140800, G(Eh)=-2953.07686408, Rel. G(kcal/mol)=2.60

10 SP_EE(Eh)=-2954.17851645, gcorr(Eh)=1.10193931, G(Eh)=-2953.07657714, Rel. G(kcal/mol)=2.78

11 SP_EE(Eh)=-2954.17648755, gcorr(Eh)=1.10003799, G(Eh)=-2953.07644956, Rel. G(kcal/mol)=2.86

12 SP_EE(Eh)=-2954.17678324, gcorr(Eh)=1.10040969, G(Eh)=-2953.07637355, Rel. G(kcal/mol)=2.91

13 SP_EE(Eh)=-2954.17674426, gcorr(Eh)=1.10038647, G(Eh)=-2953.07635779, Rel. G(kcal/mol)=2.92

14 SP_EE(Eh)=-2954.17635103, gcorr(Eh)=1.10007435, G(Eh)=-2953.07627668, Rel. G(kcal/mol)=2.97

15 SP_EE(Eh)=-2954.17575377, gcorr(Eh)=1.09951741, G(Eh)=-2953.07623636, Rel. G(kcal/mol)=2.99

16 SP_EE(Eh)=-2954.17694627, gcorr(Eh)=1.10111544, G(Eh)=-2953.07583083, Rel. G(kcal/mol)=3.25

17 SP_EE(Eh)=-2954.17746406, gcorr(Eh)=1.10200409, G(Eh)=-2953.07545997, Rel. G(kcal/mol)=3.48

18 SP_EE(Eh)=-2954.17544140, gcorr(Eh)=1.09998834, G(Eh)=-2953.07545306, Rel. G(kcal/mol)=3.48

19 SP_EE(Eh)=-2954.17427769, gcorr(Eh)=1.09889469, G(Eh)=-2953.07538300, Rel. G(kcal/mol)=3.53

20 SP_EE(Eh)=-2954.17346530, gcorr(Eh)=1.09811837, G(Eh)=-2953.07534693, Rel. G(kcal/mol)=3.55

21 SP_EE(Eh)=-2954.17554577, gcorr(Eh)=1.10052059, G(Eh)=-2953.07502518, Rel. G(kcal/mol)=3.75

22 SP_EE(Eh)=-2954.17497940, gcorr(Eh)=1.10042903, G(Eh)=-2953.07455037, Rel. G(kcal/mol)=4.05

23 SP_EE(Eh)=-2954.17549397, gcorr(Eh)=1.10107089, G(Eh)=-2953.07442308, Rel. G(kcal/mol)=4.13

24 SP_EE(Eh)=-2954.17565449, gcorr(Eh)=1.10133015, G(Eh)=-2953.07432434, Rel. G(kcal/mol)=4.19

25 SP_EE(Eh)=-2954.17438833, gcorr(Eh)=1.10010274, G(Eh)=-2953.07428559, Rel. G(kcal/mol)=4.22

26 SP_EE(Eh)=-2954.17429203, gcorr(Eh)=1.10022529, G(Eh)=-2953.07406674, Rel. G(kcal/mol)=4.35

27 SP_EE(Eh)=-2954.17472940, gcorr(Eh)=1.10067124, G(Eh)=-2953.07405816, Rel. G(kcal/mol)=4.36

28 SP_EE(Eh)=-2954.17453661, gcorr(Eh)=1.10054428, G(Eh)=-2953.07399233, Rel. G(kcal/mol)=4.40

29 SP_EE(Eh)=-2954.17539880, gcorr(Eh)=1.10142860, G(Eh)=-2953.07397020, Rel. G(kcal/mol)=4.41

30 SP_EE(Eh)=-2954.17413368, gcorr(Eh)=1.10030692, G(Eh)=-2953.07382676, Rel. G(kcal/mol)=4.50

31 SP_EE(Eh)=-2954.17591096, gcorr(Eh)=1.10209533, G(Eh)=-2953.07381563, Rel. G(kcal/mol)=4.51

32 SP_EE(Eh)=-2954.17486574, gcorr(Eh)=1.10112107, G(Eh)=-2953.07374467, Rel. G(kcal/mol)=4.55

33 SP_EE(Eh)=-2954.17517070, gcorr(Eh)=1.10149651, G(Eh)=-2953.07367419, Rel. G(kcal/mol)=4.60

34 SP_EE(Eh)=-2954.17380761, gcorr(Eh)=1.10027836, G(Eh)=-2953.07352925, Rel. G(kcal/mol)=4.69

35 SP_EE(Eh)=-2954.17294242, gcorr(Eh)=1.09942139, G(Eh)=-2953.07352103, Rel. G(kcal/mol)=4.70

36 SP_EE(Eh)=-2954.17266464, gcorr(Eh)=1.09917690, G(Eh)=-2953.07348774, Rel. G(kcal/mol)=4.72

37 SP_EE(Eh)=-2954.17461984, gcorr(Eh)=1.10131016, G(Eh)=-2953.07330968, Rel. G(kcal/mol)=4.83

38 SP_EE(Eh)=-2954.17323909, gcorr(Eh)=1.10003769, G(Eh)=-2953.07320140, Rel. G(kcal/mol)=4.90

39 SP_EE(Eh)=-2954.17453816, gcorr(Eh)=1.10213096, G(Eh)=-2953.07240720, Rel. G(kcal/mol)=5.39

40 SP_EE(Eh)=-2954.17359079, gcorr(Eh)=1.10123216, G(Eh)=-2953.07235863, Rel. G(kcal/mol)=5.42

41 SP_EE(Eh)=-2954.17342111, gcorr(Eh)=1.10130111, G(Eh)=-2953.07212000, Rel. G(kcal/mol)=5.57

42 SP_EE(Eh)=-2954.17129174, gcorr(Eh)=1.09924933, G(Eh)=-2953.07204241, Rel. G(kcal/mol)=5.62

43 SP_EE(Eh)=-2954.17227431, gcorr(Eh)=1.10045505, G(Eh)=-2953.07181926, Rel. G(kcal/mol)=5.76

44 SP_EE(Eh)=-2954.17185926, gcorr(Eh)=1.10018979, G(Eh)=-2953.07166947, Rel. G(kcal/mol)=5.86

45 SP_EE(Eh)=-2954.17222452, gcorr(Eh)=1.10065823, G(Eh)=-2953.07156629, Rel. G(kcal/mol)=5.92

46 SP_EE(Eh)=-2954.17143227, gcorr(Eh)=1.10271335, G(Eh)=-2953.06871892, Rel. G(kcal/mol)=7.71

4r11.xyz

1 SP_EE(Eh)=-2570.68393673, gcorr(Eh)=1.00108566, G(Eh)=-2569.68285107, Rel. G(kcal/mol)=0.00

2 SP_EE(Eh)=-2570.68063846, gcorr(Eh)=0.99965090, G(Eh)=-2569.68098756, Rel. G(kcal/mol)=1.17

3 SP_EE(Eh)=-2570.67867240, gcorr(Eh)=0.99927447, G(Eh)=-2569.67939793, Rel. G(kcal/mol)=2.17

4 SP_EE(Eh)=-2570.67785643, gcorr(Eh)=0.99981381, G(Eh)=-2569.67804262, Rel. G(kcal/mol)=3.02

5 SP_EE(Eh)=-2570.67726550, gcorr(Eh)=0.99923913, G(Eh)=-2569.67802637, Rel. G(kcal/mol)=3.03

6 SP_EE(Eh)=-2570.67841043, gcorr(Eh)=1.00060419, G(Eh)=-2569.67780624, Rel. G(kcal/mol)=3.17

7 SP_EE(Eh)=-2570.67837801, gcorr(Eh)=1.00078610, G(Eh)=-2569.67759191, Rel. G(kcal/mol)=3.30

8 SP_EE(Eh)=-2570.67780097, gcorr(Eh)=1.00047764, G(Eh)=-2569.67732333, Rel. G(kcal/mol)=3.47

9 SP_EE(Eh)=-2570.67753167, gcorr(Eh)=1.00112453, G(Eh)=-2569.67640714, Rel. G(kcal/mol)=4.04

10 SP_EE(Eh)=-2570.67645467, gcorr(Eh)=1.00064714, G(Eh)=-2569.67580753, Rel. G(kcal/mol)=4.42

4r4.xyz

1 SP_EE(Eh)=-2686.20090672, gcorr(Eh)=1.04314789, G(Eh)=-2685.15775883, Rel. G(kcal/mol)=0.00

2 SP_EE(Eh)=-2686.19802285, gcorr(Eh)=1.04092144, G(Eh)=-2685.15710141, Rel. G(kcal/mol)=0.41

3 SP_EE(Eh)=-2686.20105934, gcorr(Eh)=1.04500323, G(Eh)=-2685.15605611, Rel. G(kcal/mol)=1.07

4r9.xyz

1 SP_EE(Eh)=-2648.10724059, gcorr(Eh)=1.03701814, G(Eh)=-2647.07022245, Rel. G(kcal/mol)=0.00

2 SP_EE(Eh)=-2648.10053624, gcorr(Eh)=1.03612898, G(Eh)=-2647.06440726, Rel. G(kcal/mol)=3.65

3 SP_EE(Eh)=-2648.09900224, gcorr(Eh)=1.03762320, G(Eh)=-2647.06137904, Rel. G(kcal/mol)=5.55

4 SP_EE(Eh)=-2648.09786619, gcorr(Eh)=1.03651230, G(Eh)=-2647.06135389, Rel. G(kcal/mol)=5.57

5 SP_EE(Eh)=-2648.09728726, gcorr(Eh)=1.03632267, G(Eh)=-2647.06096459, Rel. G(kcal/mol)=5.81

6 SP_EE(Eh)=-2648.09707356, gcorr(Eh)=1.03705276, G(Eh)=-2647.06002080, Rel. G(kcal/mol)=6.40

5e10.xyz

1 SP_EE(Eh)=-2993.49026627, gcorr(Eh)=1.13029874, G(Eh)=-2992.35996753, Rel. G(kcal/mol)=0.00

2 SP_EE(Eh)=-2993.48576879, gcorr(Eh)=1.12892444, G(Eh)=-2992.35684435, Rel. G(kcal/mol)=1.96

3 SP_EE(Eh)=-2993.48229085, gcorr(Eh)=1.12697447, G(Eh)=-2992.35531638, Rel. G(kcal/mol)=2.92

4 SP_EE(Eh)=-2993.48595973, gcorr(Eh)=1.13086475, G(Eh)=-2992.35509498, Rel. G(kcal/mol)=3.06

5 SP_EE(Eh)=-2993.48356524, gcorr(Eh)=1.12866262, G(Eh)=-2992.35490262, Rel. G(kcal/mol)=3.18

6 SP_EE(Eh)=-2993.48284528, gcorr(Eh)=1.12795689, G(Eh)=-2992.35488839, Rel. G(kcal/mol)=3.19

7 SP_EE(Eh)=-2993.48273451, gcorr(Eh)=1.12788603, G(Eh)=-2992.35484848, Rel. G(kcal/mol)=3.21

8 SP_EE(Eh)=-2993.48574691, gcorr(Eh)=1.13139458, G(Eh)=-2992.35435233, Rel. G(kcal/mol)=3.52

9 SP_EE(Eh)=-2993.48368182, gcorr(Eh)=1.12983584, G(Eh)=-2992.35384598, Rel. G(kcal/mol)=3.84

10 SP_EE(Eh)=-2993.48267046, gcorr(Eh)=1.12897117, G(Eh)=-2992.35369929, Rel. G(kcal/mol)=3.93

11 SP_EE(Eh)=-2993.48442595, gcorr(Eh)=1.13106832, G(Eh)=-2992.35335763, Rel. G(kcal/mol)=4.15

12 SP_EE(Eh)=-2993.48179858, gcorr(Eh)=1.12854754, G(Eh)=-2992.35325104, Rel. G(kcal/mol)=4.21

13 SP_EE(Eh)=-2993.48186250, gcorr(Eh)=1.12973546, G(Eh)=-2992.35212704, Rel. G(kcal/mol)=4.92

14 SP_EE(Eh)=-2993.48005147, gcorr(Eh)=1.12810952, G(Eh)=-2992.35194195, Rel. G(kcal/mol)=5.04

15 SP_EE(Eh)=-2993.48023627, gcorr(Eh)=1.12979987, G(Eh)=-2992.35043640, Rel. G(kcal/mol)=5.98

16 SP_EE(Eh)=-2993.47892903, gcorr(Eh)=1.12993881, G(Eh)=-2992.34899022, Rel. G(kcal/mol)=6.89

5e12.xyz

1 SP_EE(Eh)=-2725.49024254, gcorr(Eh)=1.06865108, G(Eh)=-2724.42159146, Rel. G(kcal/mol)=0.00

2 SP_EE(Eh)=-2725.49301067, gcorr(Eh)=1.07187709, G(Eh)=-2724.42113358, Rel. G(kcal/mol)=0.29

3 SP_EE(Eh)=-2725.48671178, gcorr(Eh)=1.06822664, G(Eh)=-2724.41848514, Rel. G(kcal/mol)=1.95

4 SP_EE(Eh)=-2725.48589720, gcorr(Eh)=1.07046572, G(Eh)=-2724.41543148, Rel. G(kcal/mol)=3.87

5 SP_EE(Eh)=-2725.48493744, gcorr(Eh)=1.07138737, G(Eh)=-2724.41355007, Rel. G(kcal/mol)=5.05

5r10.xyz

1 SP_EE(Eh)=-2993.49352386, gcorr(Eh)=1.13041411, G(Eh)=-2992.36310975, Rel. G(kcal/mol)=0.00

2 SP_EE(Eh)=-2993.48669775, gcorr(Eh)=1.13046115, G(Eh)=-2992.35623660, Rel. G(kcal/mol)=4.31

3 SP_EE(Eh)=-2993.48576752, gcorr(Eh)=1.12963478, G(Eh)=-2992.35613274, Rel. G(kcal/mol)=4.38

4 SP_EE(Eh)=-2993.48477885, gcorr(Eh)=1.12880972, G(Eh)=-2992.35596913, Rel. G(kcal/mol)=4.48

5 SP_EE(Eh)=-2993.48448366, gcorr(Eh)=1.12942963, G(Eh)=-2992.35505403, Rel. G(kcal/mol)=5.06

6 SP_EE(Eh)=-2993.48311899, gcorr(Eh)=1.12817100, G(Eh)=-2992.35494799, Rel. G(kcal/mol)=5.12

7 SP_EE(Eh)=-2993.48350677, gcorr(Eh)=1.12909062, G(Eh)=-2992.35441615, Rel. G(kcal/mol)=5.46

8 SP_EE(Eh)=-2993.48239905, gcorr(Eh)=1.12890843, G(Eh)=-2992.35349062, Rel. G(kcal/mol)=6.04

9 SP_EE(Eh)=-2993.48246404, gcorr(Eh)=1.12926534, G(Eh)=-2992.35319870, Rel. G(kcal/mol)=6.22

10 SP_EE(Eh)=-2993.48124880, gcorr(Eh)=1.12807103, G(Eh)=-2992.35317777, Rel. G(kcal/mol)=6.23

11 SP_EE(Eh)=-2993.48099539, gcorr(Eh)=1.12870915, G(Eh)=-2992.35228624, Rel. G(kcal/mol)=6.79

12 SP_EE(Eh)=-2993.48115321, gcorr(Eh)=1.12911933, G(Eh)=-2992.35203388, Rel. G(kcal/mol)=6.95

13 SP_EE(Eh)=-2993.48110950, gcorr(Eh)=1.12951133, G(Eh)=-2992.35159817, Rel. G(kcal/mol)=7.22

14 SP_EE(Eh)=-2993.48093041, gcorr(Eh)=1.12999907, G(Eh)=-2992.35093134, Rel. G(kcal/mol)=7.64

15 SP_EE(Eh)=-2993.47874113, gcorr(Eh)=1.12835750, G(Eh)=-2992.35038363, Rel. G(kcal/mol)=7.99

5r12.xyz

1 SP_EE(Eh)=-2725.49371121, gcorr(Eh)=1.06917991, G(Eh)=-2724.42453130, Rel. G(kcal/mol)=0.00

2 SP_EE(Eh)=-2725.49383831, gcorr(Eh)=1.07198425, G(Eh)=-2724.42185406, Rel. G(kcal/mol)=1.68

3 SP_EE(Eh)=-2725.48848069, gcorr(Eh)=1.07064657, G(Eh)=-2724.41783412, Rel. G(kcal/mol)=4.20

4 SP_EE(Eh)=-2725.48575105, gcorr(Eh)=1.06964071, G(Eh)=-2724.41611034, Rel. G(kcal/mol)=5.28

6e11.xyz

1 SP_EE(Eh)=-2907.81729042, gcorr(Eh)=1.00282464, G(Eh)=-2906.81446578, Rel. G(kcal/mol)=0.00

2 SP_EE(Eh)=-2907.81604368, gcorr(Eh)=1.00295740, G(Eh)=-2906.81308628, Rel. G(kcal/mol)=0.87

3 SP_EE(Eh)=-2907.81375964, gcorr(Eh)=1.00164719, G(Eh)=-2906.81211245, Rel. G(kcal/mol)=1.48

4 SP_EE(Eh)=-2907.81350875, gcorr(Eh)=1.00245916, G(Eh)=-2906.81104959, Rel. G(kcal/mol)=2.14

5 SP_EE(Eh)=-2907.81285298, gcorr(Eh)=1.00328543, G(Eh)=-2906.80956755, Rel. G(kcal/mol)=3.07

6 SP_EE(Eh)=-2907.81172551, gcorr(Eh)=1.00253914, G(Eh)=-2906.80918637, Rel. G(kcal/mol)=3.31

7 SP_EE(Eh)=-2907.81239384, gcorr(Eh)=1.00329411, G(Eh)=-2906.80909973, Rel. G(kcal/mol)=3.37

8 SP_EE(Eh)=-2907.81017136, gcorr(Eh)=1.00148582, G(Eh)=-2906.80868554, Rel. G(kcal/mol)=3.63

9 SP_EE(Eh)=-2907.81094046, gcorr(Eh)=1.00340814, G(Eh)=-2906.80753232, Rel. G(kcal/mol)=4.35

10 SP_EE(Eh)=-2907.80952203, gcorr(Eh)=1.00199500, G(Eh)=-2906.80752703, Rel. G(kcal/mol)=4.35

11 SP_EE(Eh)=-2907.81092681, gcorr(Eh)=1.00371307, G(Eh)=-2906.80721374, Rel. G(kcal/mol)=4.55

12 SP_EE(Eh)=-2907.81021372, gcorr(Eh)=1.00313482, G(Eh)=-2906.80707890, Rel. G(kcal/mol)=4.64

13 SP_EE(Eh)=-2907.80987344, gcorr(Eh)=1.00289448, G(Eh)=-2906.80697896, Rel. G(kcal/mol)=4.70

14 SP_EE(Eh)=-2907.80779830, gcorr(Eh)=1.00097604, G(Eh)=-2906.80682226, Rel. G(kcal/mol)=4.80

15 SP_EE(Eh)=-2907.80879375, gcorr(Eh)=1.00201039, G(Eh)=-2906.80678336, Rel. G(kcal/mol)=4.82

16 SP_EE(Eh)=-2907.80867984, gcorr(Eh)=1.00195044, G(Eh)=-2906.80672940, Rel. G(kcal/mol)=4.85

17 SP_EE(Eh)=-2907.80960602, gcorr(Eh)=1.00333917, G(Eh)=-2906.80626685, Rel. G(kcal/mol)=5.14

18 SP_EE(Eh)=-2907.80723776, gcorr(Eh)=1.00208102, G(Eh)=-2906.80515674, Rel. G(kcal/mol)=5.84

19 SP_EE(Eh)=-2907.80612146, gcorr(Eh)=1.00179647, G(Eh)=-2906.80432499, Rel. G(kcal/mol)=6.36

20 SP_EE(Eh)=-2907.80537761, gcorr(Eh)=1.00118767, G(Eh)=-2906.80418994, Rel. G(kcal/mol)=6.45

21 SP_EE(Eh)=-2907.80674701, gcorr(Eh)=1.00273628, G(Eh)=-2906.80401073, Rel. G(kcal/mol)=6.56

22 SP_EE(Eh)=-2907.80634987, gcorr(Eh)=1.00288002, G(Eh)=-2906.80346985, Rel. G(kcal/mol)=6.90

23 SP_EE(Eh)=-2907.80633802, gcorr(Eh)=1.00303050, G(Eh)=-2906.80330752, Rel. G(kcal/mol)=7.00

24 SP_EE(Eh)=-2907.80700898, gcorr(Eh)=1.00389104, G(Eh)=-2906.80311794, Rel. G(kcal/mol)=7.12

25 SP_EE(Eh)=-2907.80493220, gcorr(Eh)=1.00257469, G(Eh)=-2906.80235751, Rel. G(kcal/mol)=7.60

26 SP_EE(Eh)=-2907.80675390, gcorr(Eh)=1.00441509, G(Eh)=-2906.80233881, Rel. G(kcal/mol)=7.61

27 SP_EE(Eh)=-2907.80511679, gcorr(Eh)=1.00371824, G(Eh)=-2906.80139855, Rel. G(kcal/mol)=8.20

28 SP_EE(Eh)=-2907.80565265, gcorr(Eh)=1.00502722, G(Eh)=-2906.80062543, Rel. G(kcal/mol)=8.68

6r11.xyz

1 SP_EE(Eh)=-2907.82325125, gcorr(Eh)=1.00109346, G(Eh)=-2906.82215779, Rel. G(kcal/mol)=0.00

2 SP_EE(Eh)=-2907.82063857, gcorr(Eh)=1.00359299, G(Eh)=-2906.81704558, Rel. G(kcal/mol)=3.21

3 SP_EE(Eh)=-2907.81647019, gcorr(Eh)=1.00239038, G(Eh)=-2906.81407981, Rel. G(kcal/mol)=5.07

4 SP_EE(Eh)=-2907.81304387, gcorr(Eh)=1.00284446, G(Eh)=-2906.81019941, Rel. G(kcal/mol)=7.50

5 SP_EE(Eh)=-2907.81183266, gcorr(Eh)=1.00375804, G(Eh)=-2906.80807462, Rel. G(kcal/mol)=8.84

7e12.xyz

1 SP_EE(Eh)=-3531.53506915, gcorr(Eh)=1.16112565, G(Eh)=-3530.37394350, Rel. G(kcal/mol)=0.00

2 SP_EE(Eh)=-3531.53243589, gcorr(Eh)=1.16020146, G(Eh)=-3530.37223443, Rel. G(kcal/mol)=1.07

3 SP_EE(Eh)=-3531.53179491, gcorr(Eh)=1.15992109, G(Eh)=-3530.37187382, Rel. G(kcal/mol)=1.30

4 SP_EE(Eh)=-3531.53223057, gcorr(Eh)=1.16093921, G(Eh)=-3530.37129136, Rel. G(kcal/mol)=1.66

5 SP_EE(Eh)=-3531.53178895, gcorr(Eh)=1.16152351, G(Eh)=-3530.37026544, Rel. G(kcal/mol)=2.31

6 SP_EE(Eh)=-3531.53065453, gcorr(Eh)=1.16042487, G(Eh)=-3530.37022966, Rel. G(kcal/mol)=2.33

7 SP_EE(Eh)=-3531.52898640, gcorr(Eh)=1.15887106, G(Eh)=-3530.37011534, Rel. G(kcal/mol)=2.40

8 SP_EE(Eh)=-3531.53231802, gcorr(Eh)=1.16257807, G(Eh)=-3530.36973995, Rel. G(kcal/mol)=2.64

9 SP_EE(Eh)=-3531.52952642, gcorr(Eh)=1.16035246, G(Eh)=-3530.36917396, Rel. G(kcal/mol)=2.99

10 SP_EE(Eh)=-3531.52941116, gcorr(Eh)=1.16082584, G(Eh)=-3530.36858532, Rel. G(kcal/mol)=3.36

11 SP_EE(Eh)=-3531.53006654, gcorr(Eh)=1.16163726, G(Eh)=-3530.36842928, Rel. G(kcal/mol)=3.46

12 SP_EE(Eh)=-3531.53130742, gcorr(Eh)=1.16305648, G(Eh)=-3530.36825094, Rel. G(kcal/mol)=3.57

13 SP_EE(Eh)=-3531.52608925, gcorr(Eh)=1.15904363, G(Eh)=-3530.36704562, Rel. G(kcal/mol)=4.33

14 SP_EE(Eh)=-3531.52827031, gcorr(Eh)=1.16229108, G(Eh)=-3530.36597923, Rel. G(kcal/mol)=5.00

15 SP_EE(Eh)=-3531.52451517, gcorr(Eh)=1.15906601, G(Eh)=-3530.36544916, Rel. G(kcal/mol)=5.33

7r12.xyz

1 SP_EE(Eh)=-3531.53986883, gcorr(Eh)=1.15990813, G(Eh)=-3530.37996070, Rel. G(kcal/mol)=0.00

2 SP_EE(Eh)=-3531.53844412, gcorr(Eh)=1.16130404, G(Eh)=-3530.37714008, Rel. G(kcal/mol)=1.77

3 SP_EE(Eh)=-3531.53847921, gcorr(Eh)=1.16182623, G(Eh)=-3530.37665298, Rel. G(kcal/mol)=2.08

4 SP_EE(Eh)=-3531.53432147, gcorr(Eh)=1.15986273, G(Eh)=-3530.37445874, Rel. G(kcal/mol)=3.45

5 SP_EE(Eh)=-3531.53314695, gcorr(Eh)=1.16050608, G(Eh)=-3530.37264087, Rel. G(kcal/mol)=4.59

6 SP_EE(Eh)=-3531.53443339, gcorr(Eh)=1.16210833, G(Eh)=-3530.37232506, Rel. G(kcal/mol)=4.79

7 SP_EE(Eh)=-3531.52829127, gcorr(Eh)=1.15918537, G(Eh)=-3530.36910590, Rel. G(kcal/mol)=6.81

8 SP_EE(Eh)=-3531.53158950, gcorr(Eh)=1.16377921, G(Eh)=-3530.36781029, Rel. G(kcal/mol)=7.62

2.2. TS-like ensembles

1e6_ts-like.xyz

1 SP_EE(Eh)=-3483.15986615, gcorr(Eh)=1.13541558, G(Eh)=-3482.02445057, Rel. G(kcal/mol)=0.00

2 SP_EE(Eh)=-3483.15969723, gcorr(Eh)=1.13614343, G(Eh)=-3482.02355380, Rel. G(kcal/mol)=0.56

3 SP_EE(Eh)=-3483.15987057, gcorr(Eh)=1.13649565, G(Eh)=-3482.02337492, Rel. G(kcal/mol)=0.67

1r6_ts-like.xyz

1 SP_EE(Eh)=-3483.15808078, gcorr(Eh)=1.13465102, G(Eh)=-3482.02342976, Rel. G(kcal/mol)=0.00

2 SP_EE(Eh)=-3483.15697605, gcorr(Eh)=1.13363181, G(Eh)=-3482.02334424, Rel. G(kcal/mol)=0.05

3 SP_EE(Eh)=-3483.15471532, gcorr(Eh)=1.13172995, G(Eh)=-3482.02298537, Rel. G(kcal/mol)=0.28

4 SP_EE(Eh)=-3483.15708150, gcorr(Eh)=1.13488633, G(Eh)=-3482.02219517, Rel. G(kcal/mol)=0.77

5 SP_EE(Eh)=-3483.15579349, gcorr(Eh)=1.13490583, G(Eh)=-3482.02088766, Rel. G(kcal/mol)=1.60

6 SP_EE(Eh)=-3483.15691844, gcorr(Eh)=1.13653654, G(Eh)=-3482.02038190, Rel. G(kcal/mol)=1.91

2e9_ts-like.xyz

1 SP_EE(Eh)=-2687.39080398, gcorr(Eh)=1.06239749, G(Eh)=-2686.32840649, Rel. G(kcal/mol)=0.00

2 SP_EE(Eh)=-2687.39231390, gcorr(Eh)=1.06446446, G(Eh)=-2686.32784944, Rel. G(kcal/mol)=0.35

3 SP_EE(Eh)=-2687.38987889, gcorr(Eh)=1.06232480, G(Eh)=-2686.32755409, Rel. G(kcal/mol)=0.53

4 SP_EE(Eh)=-2687.38816355, gcorr(Eh)=1.06114815, G(Eh)=-2686.32701540, Rel. G(kcal/mol)=0.87

5 SP_EE(Eh)=-2687.39063507, gcorr(Eh)=1.06489496, G(Eh)=-2686.32574011, Rel. G(kcal/mol)=1.67

6 SP_EE(Eh)=-2687.38927148, gcorr(Eh)=1.06358927, G(Eh)=-2686.32568221, Rel. G(kcal/mol)=1.71

2r9_ts-like.xyz

1 SP_EE(Eh)=-2687.39294363, gcorr(Eh)=1.06275594, G(Eh)=-2686.33018769, Rel. G(kcal/mol)=0.00

2 SP_EE(Eh)=-2687.39008788, gcorr(Eh)=1.06168065, G(Eh)=-2686.32840723, Rel. G(kcal/mol)=1.12

3 SP_EE(Eh)=-2687.39269135, gcorr(Eh)=1.06444560, G(Eh)=-2686.32824575, Rel. G(kcal/mol)=1.22

3e4_ts-like.xyz

1 SP_EE(Eh)=-2686.19835258, gcorr(Eh)=1.04190330, G(Eh)=-2685.15644928, Rel. G(kcal/mol)=0.00

2 SP_EE(Eh)=-2686.19805485, gcorr(Eh)=1.04280988, G(Eh)=-2685.15524497, Rel. G(kcal/mol)=0.76

3 SP_EE(Eh)=-2686.19859857, gcorr(Eh)=1.04380497, G(Eh)=-2685.15479360, Rel. G(kcal/mol)=1.04

4 SP_EE(Eh)=-2686.19761396, gcorr(Eh)=1.04302941, G(Eh)=-2685.15458455, Rel. G(kcal/mol)=1.17

5 SP_EE(Eh)=-2686.19839737, gcorr(Eh)=1.04406833, G(Eh)=-2685.15432904, Rel. G(kcal/mol)=1.33

6 SP_EE(Eh)=-2686.19678176, gcorr(Eh)=1.04274319, G(Eh)=-2685.15403857, Rel. G(kcal/mol)=1.51

7 SP_EE(Eh)=-2686.19879227, gcorr(Eh)=1.04480395, G(Eh)=-2685.15398832, Rel. G(kcal/mol)=1.54

8 SP_EE(Eh)=-2686.19618627, gcorr(Eh)=1.04222117, G(Eh)=-2685.15396510, Rel. G(kcal/mol)=1.56

3e6_ts-like.xyz

1 SP_EE(Eh)=-3443.87031818, gcorr(Eh)=1.10976061, G(Eh)=-3442.76055757, Rel. G(kcal/mol)=0.00

3r4_ts-like.xyz

1 SP_EE(Eh)=-2686.19471817, gcorr(Eh)=1.04262020, G(Eh)=-2685.15209797, Rel. G(kcal/mol)=0.00

2 SP_EE(Eh)=-2686.19629279, gcorr(Eh)=1.04424229, G(Eh)=-2685.15205050, Rel. G(kcal/mol)=0.03

3 SP_EE(Eh)=-2686.19411241, gcorr(Eh)=1.04221155, G(Eh)=-2685.15190086, Rel. G(kcal/mol)=0.12

4 SP_EE(Eh)=-2686.19556570, gcorr(Eh)=1.04375116, G(Eh)=-2685.15181454, Rel. G(kcal/mol)=0.18

5 SP_EE(Eh)=-2686.19274878, gcorr(Eh)=1.04103717, G(Eh)=-2685.15171161, Rel. G(kcal/mol)=0.24

6 SP_EE(Eh)=-2686.19611189, gcorr(Eh)=1.04457362, G(Eh)=-2685.15153827, Rel. G(kcal/mol)=0.35

7 SP_EE(Eh)=-2686.19506904, gcorr(Eh)=1.04359930, G(Eh)=-2685.15146974, Rel. G(kcal/mol)=0.39

8 SP_EE(Eh)=-2686.19569550, gcorr(Eh)=1.04426553, G(Eh)=-2685.15142997, Rel. G(kcal/mol)=0.42

9 SP_EE(Eh)=-2686.19457238, gcorr(Eh)=1.04370028, G(Eh)=-2685.15087210, Rel. G(kcal/mol)=0.77

10 SP_EE(Eh)=-2686.19453091, gcorr(Eh)=1.04402612, G(Eh)=-2685.15050479, Rel. G(kcal/mol)=1.00

11 SP_EE(Eh)=-2686.19356029, gcorr(Eh)=1.04310835, G(Eh)=-2685.15045194, Rel. G(kcal/mol)=1.03

12 SP_EE(Eh)=-2686.19555242, gcorr(Eh)=1.04525543, G(Eh)=-2685.15029699, Rel. G(kcal/mol)=1.13

13 SP_EE(Eh)=-2686.19613771, gcorr(Eh)=1.04584837, G(Eh)=-2685.15028934, Rel. G(kcal/mol)=1.13

14 SP_EE(Eh)=-2686.19321998, gcorr(Eh)=1.04342063, G(Eh)=-2685.14979935, Rel. G(kcal/mol)=1.44

3r6_ts-like.xyz

1 SP_EE(Eh)=-3443.86900278, gcorr(Eh)=1.10749804, G(Eh)=-3442.76150474, Rel. G(kcal/mol)=0.00

2 SP_EE(Eh)=-3443.86899495, gcorr(Eh)=1.10911145, G(Eh)=-3442.75988350, Rel. G(kcal/mol)=1.02

3 SP_EE(Eh)=-3443.86854131, gcorr(Eh)=1.10876591, G(Eh)=-3442.75977540, Rel. G(kcal/mol)=1.09

4 SP_EE(Eh)=-3443.86668521, gcorr(Eh)=1.10719757, G(Eh)=-3442.75948764, Rel. G(kcal/mol)=1.27

5 SP_EE(Eh)=-3443.86706451, gcorr(Eh)=1.10797958, G(Eh)=-3442.75908493, Rel. G(kcal/mol)=1.52

4e10_ts-like.xyz

1 SP_EE(Eh)=-2954.18220082, gcorr(Eh)=1.09992111, G(Eh)=-2953.08227971, Rel. G(kcal/mol)=0.00

2 SP_EE(Eh)=-2954.18003606, gcorr(Eh)=1.09915044, G(Eh)=-2953.08088562, Rel. G(kcal/mol)=0.87

3 SP_EE(Eh)=-2954.18099252, gcorr(Eh)=1.10081684, G(Eh)=-2953.08017568, Rel. G(kcal/mol)=1.32

4 SP_EE(Eh)=-2954.17964659, gcorr(Eh)=1.10053354, G(Eh)=-2953.07911305, Rel. G(kcal/mol)=1.99

4e11_ts-like.xyz

1 SP_EE(Eh)=-2570.68055147, gcorr(Eh)=1.00008053, G(Eh)=-2569.68047094, Rel. G(kcal/mol)=0.00

2 SP_EE(Eh)=-2570.68050781, gcorr(Eh)=1.00023161, G(Eh)=-2569.68027620, Rel. G(kcal/mol)=0.12

3 SP_EE(Eh)=-2570.67956140, gcorr(Eh)=0.99957047, G(Eh)=-2569.67999093, Rel. G(kcal/mol)=0.30

4 SP_EE(Eh)=-2570.67946207, gcorr(Eh)=1.00071296, G(Eh)=-2569.67874911, Rel. G(kcal/mol)=1.08

5 SP_EE(Eh)=-2570.67886166, gcorr(Eh)=1.00024734, G(Eh)=-2569.67861432, Rel. G(kcal/mol)=1.17

6 SP_EE(Eh)=-2570.67884983, gcorr(Eh)=1.00053021, G(Eh)=-2569.67831962, Rel. G(kcal/mol)=1.35

7 SP_EE(Eh)=-2570.67838450, gcorr(Eh)=1.00034363, G(Eh)=-2569.67804087, Rel. G(kcal/mol)=1.52

8 SP_EE(Eh)=-2570.67744066, gcorr(Eh)=0.99962971, G(Eh)=-2569.67781095, Rel. G(kcal/mol)=1.67

9 SP_EE(Eh)=-2570.67621411, gcorr(Eh)=0.99873764, G(Eh)=-2569.67747647, Rel. G(kcal/mol)=1.88

10 SP_EE(Eh)=-2570.67608529, gcorr(Eh)=0.99868406, G(Eh)=-2569.67740123, Rel. G(kcal/mol)=1.93

11 SP_EE(Eh)=-2570.67740911, gcorr(Eh)=1.00004982, G(Eh)=-2569.67735929, Rel. G(kcal/mol)=1.95

4e4_ts-like.xyz

1 SP_EE(Eh)=-2686.20062702, gcorr(Eh)=1.04168500, G(Eh)=-2685.15894202, Rel. G(kcal/mol)=0.00

2 SP_EE(Eh)=-2686.19793574, gcorr(Eh)=1.04103900, G(Eh)=-2685.15689674, Rel. G(kcal/mol)=1.28

4e9_ts-like.xyz

1 SP_EE(Eh)=-2648.10200750, gcorr(Eh)=1.03569891, G(Eh)=-2647.06630859, Rel. G(kcal/mol)=0.00

2 SP_EE(Eh)=-2648.10058275, gcorr(Eh)=1.03615489, G(Eh)=-2647.06442786, Rel. G(kcal/mol)=1.18

3 SP_EE(Eh)=-2648.10148198, gcorr(Eh)=1.03709329, G(Eh)=-2647.06438869, Rel. G(kcal/mol)=1.20

4 SP_EE(Eh)=-2648.09998911, gcorr(Eh)=1.03684509, G(Eh)=-2647.06314402, Rel. G(kcal/mol)=1.99

4r10_ts-like.xyz

1 SP_EE(Eh)=-2954.18127852, gcorr(Eh)=1.10027530, G(Eh)=-2953.08100322, Rel. G(kcal/mol)=0.00

2 SP_EE(Eh)=-2954.17982277, gcorr(Eh)=1.10053991, G(Eh)=-2953.07928286, Rel. G(kcal/mol)=1.08

3 SP_EE(Eh)=-2954.17953505, gcorr(Eh)=1.10050211, G(Eh)=-2953.07903294, Rel. G(kcal/mol)=1.24

4 SP_EE(Eh)=-2954.17922776, gcorr(Eh)=1.10124408, G(Eh)=-2953.07798368, Rel. G(kcal/mol)=1.89

4r11_ts-like.xyz

1 SP_EE(Eh)=-2570.68393673, gcorr(Eh)=1.00108566, G(Eh)=-2569.68285107, Rel. G(kcal/mol)=0.00

2 SP_EE(Eh)=-2570.68063846, gcorr(Eh)=0.99965090, G(Eh)=-2569.68098756, Rel. G(kcal/mol)=1.17

4r4_ts-like.xyz

1 SP_EE(Eh)=-2686.20090672, gcorr(Eh)=1.04314789, G(Eh)=-2685.15775883, Rel. G(kcal/mol)=0.00

2 SP_EE(Eh)=-2686.19802285, gcorr(Eh)=1.04092144, G(Eh)=-2685.15710141, Rel. G(kcal/mol)=0.41

3 SP_EE(Eh)=-2686.20105934, gcorr(Eh)=1.04500323, G(Eh)=-2685.15605611, Rel. G(kcal/mol)=1.07

4r9_ts-like.xyz

1 SP_EE(Eh)=-2648.10724059, gcorr(Eh)=1.03701814, G(Eh)=-2647.07022245, Rel. G(kcal/mol)=0.00

5e10_ts-like.xyz

1 SP_EE(Eh)=-2993.49026627, gcorr(Eh)=1.13029874, G(Eh)=-2992.35996753, Rel. G(kcal/mol)=0.00

2 SP_EE(Eh)=-2993.48573528, gcorr(Eh)=1.12707216, G(Eh)=-2992.35866312, Rel. G(kcal/mol)=0.82

3 SP_EE(Eh)=-2993.48690204, gcorr(Eh)=1.12853736, G(Eh)=-2992.35836468, Rel. G(kcal/mol)=1.01

4 SP_EE(Eh)=-2993.48576879, gcorr(Eh)=1.12892444, G(Eh)=-2992.35684435, Rel. G(kcal/mol)=1.96

5e12_ts-like.xyz

1 SP_EE(Eh)=-2725.49024254, gcorr(Eh)=1.06865108, G(Eh)=-2724.42159146, Rel. G(kcal/mol)=0.00

2 SP_EE(Eh)=-2725.49301067, gcorr(Eh)=1.07187709, G(Eh)=-2724.42113358, Rel. G(kcal/mol)=0.29

3 SP_EE(Eh)=-2725.48671178, gcorr(Eh)=1.06822664, G(Eh)=-2724.41848514, Rel. G(kcal/mol)=1.95

5r10_ts-like.xyz

1 SP_EE(Eh)=-2993.49352386, gcorr(Eh)=1.13041411, G(Eh)=-2992.36310975, Rel. G(kcal/mol)=0.00

5r12_ts-like.xyz

1 SP_EE(Eh)=-2725.49383831, gcorr(Eh)=1.07198425, G(Eh)=-2724.42185406, Rel. G(kcal/mol)=0.00

6e11_ts-like.xyz

1 SP_EE(Eh)=-2907.81729042, gcorr(Eh)=1.00282464, G(Eh)=-2906.81446578, Rel. G(kcal/mol)=0.00

2 SP_EE(Eh)=-2907.81604368, gcorr(Eh)=1.00295740, G(Eh)=-2906.81308628, Rel. G(kcal/mol)=0.87

3 SP_EE(Eh)=-2907.81375964, gcorr(Eh)=1.00164719, G(Eh)=-2906.81211245, Rel. G(kcal/mol)=1.48

6r11_ts-like.xyz

1 SP_EE(Eh)=-2907.82325125, gcorr(Eh)=1.00109346, G(Eh)=-2906.82215779, Rel. G(kcal/mol)=0.00

7e12_ts-like.xyz

1 SP_EE(Eh)=-3531.53506915, gcorr(Eh)=1.16112565, G(Eh)=-3530.37394350, Rel. G(kcal/mol)=0.00

2 SP_EE(Eh)=-3531.53243589, gcorr(Eh)=1.16020146, G(Eh)=-3530.37223443, Rel. G(kcal/mol)=1.07

3 SP_EE(Eh)=-3531.53179491, gcorr(Eh)=1.15992109, G(Eh)=-3530.37187382, Rel. G(kcal/mol)=1.30

4 SP_EE(Eh)=-3531.53223057, gcorr(Eh)=1.16093921, G(Eh)=-3530.37129136, Rel. G(kcal/mol)=1.66

7r12_ts-like.xyz

1 SP_EE(Eh)=-3531.53847921, gcorr(Eh)=1.16182623, G(Eh)=-3530.37665298, Rel. G(kcal/mol)=0.00

2 SP_EE(Eh)=-3531.53432147, gcorr(Eh)=1.15986273, G(Eh)=-3530.37445874, Rel. G(kcal/mol)=1.38

Regioselective Primary Alcohol Oxidation: Energetic Dataset

OAzcP7_free.xyz

1 G=-3411.599724089610 Eh - Rel. G.=0.000 kcal/mol - G(corr)(Eh)=1.04395247

2 G=-3411.598551098119 Eh - Rel. G.=0.736 kcal/mol - G(corr)(Eh)=1.04222229

3 G=-3411.598415740634 Eh - Rel. G.=0.821 kcal/mol - G(corr)(Eh)=1.04331813

4 G=-3411.598361333003 Eh - Rel. G.=0.855 kcal/mol - G(corr)(Eh)=1.04382897

5 G=-3411.597542519802 Eh - Rel. G.=1.369 kcal/mol - G(corr)(Eh)=1.04153938

6 G=-3411.596744540123 Eh - Rel. G.=1.870 kcal/mol - G(corr)(Eh)=1.04310644

OAzcP7_hindered.xyz

1 G=-3411.6044560382097 Eh - Rel. G.=0.000 kcal/mol - G(corr)(Eh)=1.04345094

2 G=-3411.6039091052960 Eh - Rel. G.=0.343 kcal/mol - G(corr)(Eh)=1.04269571

3 G=-3411.6035832716793 Eh - Rel. G.=0.548 kcal/mol - G(corr)(Eh)=1.04263435

4 G=-3411.6026579146410 Eh - Rel. G.=1.128 kcal/mol - G(corr)(Eh)=1.04303523

5 G=-3411.6013004289170 Eh - Rel. G.=1.980 kcal/mol - G(corr)(Eh)=1.04273948

2.3. Ensemble sizes

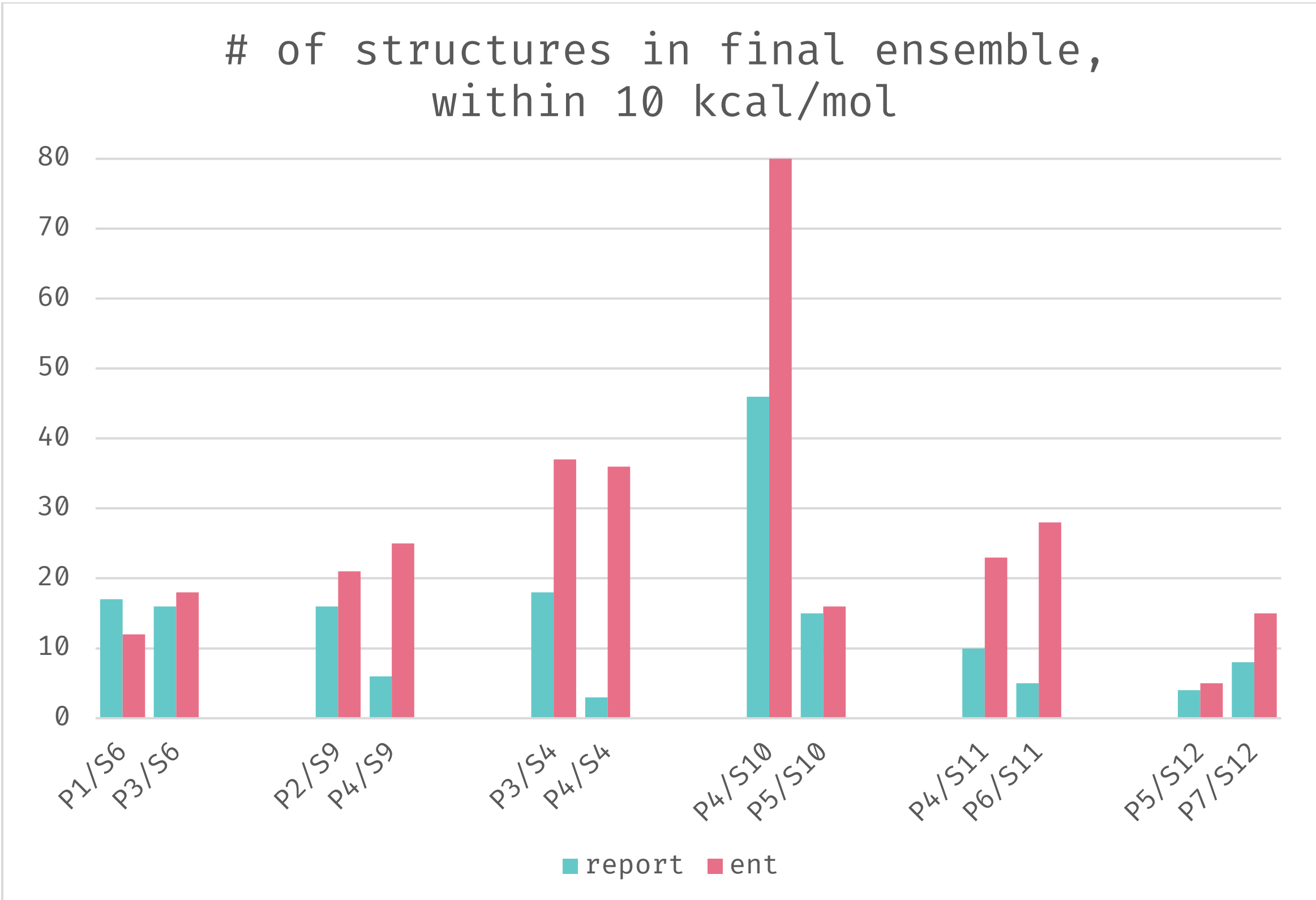

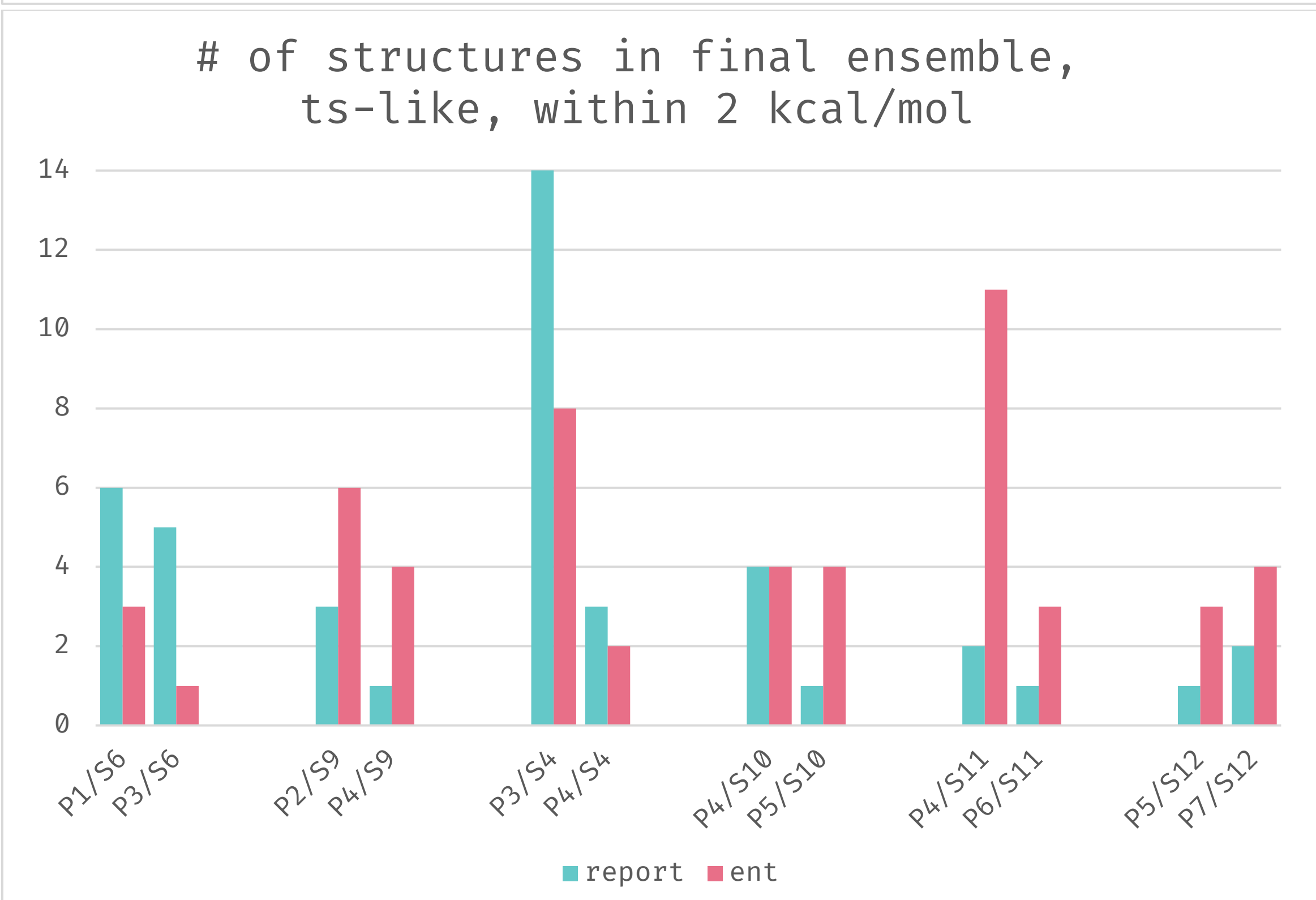

2.4. Additional P7 modeling

The ground state complexes of additional substrates (**S4**, **S10**, **S15**) with **P7** were modeled to probe the activation mode landscape of the optimized catalyst with different substrates.

P7_S10_ent.xyz

```
1  G = -3798.341551484714 Eh - Rel. G. = 0.000 kcal/mol - G(corr)(Eh) = 1.19195630

2  G = -3798.341308032216 Eh - Rel. G. = 0.153 kcal/mol - G(corr)(Eh) = 1.19203484

3  G = -3798.341206137354 Eh - Rel. G. = 0.217 kcal/mol - G(corr)(Eh) = 1.19222940

4  G = -3798.339464487129 Eh - Rel. G. = 1.310 kcal/mol - G(corr)(Eh) = 1.19233828

5  G = -3798.336031973637 Eh - Rel. G. = 3.464 kcal/mol - G(corr)(Eh) = 1.19164203

6  G = -3798.334619305918 Eh - Rel. G. = 4.350 kcal/mol - G(corr)(Eh) = 1.19111190

7  G = -3798.333586734929 Eh - Rel. G. = 4.998 kcal/mol - G(corr)(Eh) = 1.19132989

8  G = -3798.332170816900 Eh - Rel. G. = 5.886 kcal/mol - G(corr)(Eh) = 1.19066223

9  G = -3798.332146405581 Eh - Rel. G. = 5.902 kcal/mol - G(corr)(Eh) = 1.19065667

10 G = -3798.329143501202 Eh - Rel. G. = 7.786 kcal/mol - G(corr)(Eh) = 1.18936361

11 G = -3798.329057090994 Eh - Rel. G. = 7.840 kcal/mol - G(corr)(Eh) = 1.19140744

12 G = -3798.328226017899 Eh - Rel. G. = 8.362 kcal/mol - G(corr)(Eh) = 1.18931243

13 G = -3798.328013169582 Eh - Rel. G. = 8.495 kcal/mol - G(corr)(Eh) = 1.18904874

14 G = -3798.327780749122 Eh - Rel. G. = 8.641 kcal/mol - G(corr)(Eh) = 1.18984190
```

P7_S10_rep

```
1  G = -3798.350060709359  Eh - Rel. G. = 0.000 kcal/mol - G(corr)(Eh) = 1.19298362

2  G = -3798.3497802981133 Eh - Rel. G. = 0.176 kcal/mol - G(corr)(Eh) = 1.19338411

3  G = -3798.349426467118  Eh - Rel. G. = 0.398 kcal/mol - G(corr)(Eh) = 1.19362180

4  G = -3798.348852166775  Eh - Rel. G. = 0.758 kcal/mol - G(corr)(Eh) = 1.19245287

5  G = -3798.348055562768  Eh - Rel. G. = 1.258 kcal/mol - G(corr)(Eh) = 1.19259269

6  G = -3798.346445947092  Eh - Rel. G. = 2.268 kcal/mol - G(corr)(Eh) = 1.19268253

7  G = -3798.3461473101856 Eh - Rel. G. = 2.456 kcal/mol - G(corr)(Eh) = 1.19413479

8  G = -3798.3447399391544 Eh - Rel. G. = 3.339 kcal/mol - G(corr)(Eh) = 1.19225123

9  G = -3798.343607442884  Eh - Rel. G. = 4.049 kcal/mol - G(corr)(Eh) = 1.19344589

10 G = -3798.342000607248  Eh - Rel. G. = 5.058 kcal/mol - G(corr)(Eh) = 1.19266831

11 G = -3798.3391758642288 Eh - Rel. G. = 6.830 kcal/mol - G(corr)(Eh) = 1.19248452

12 G = -3798.3389662034    Eh - Rel. G. = 6.962 kcal/mol - G(corr)(Eh) = 1.19242710

13 G = -3798.338752078407  Eh - Rel. G. = 7.096 kcal/mol - G(corr)(Eh) = 1.19090441
```

P7_S15_ent

1 G = -3374.455945241312 Eh - Rel. G. = 0.000 kcal/mol - G(corr)(Eh) = 1.04440371

2 G = -3374.454031474744 Eh - Rel. G. = 1.201 kcal/mol - G(corr)(Eh) = 1.04367172

3 G = -3374.453676364536 Eh - Rel. G. = 1.424 kcal/mol - G(corr)(Eh) = 1.04497289

4 G = -3374.453567616183 Eh - Rel. G. = 1.492 kcal/mol - G(corr)(Eh) = 1.04620935

5 G = -3374.451570197085 Eh - Rel. G. = 2.745 kcal/mol - G(corr)(Eh) = 1.04676664

6 G = -3374.450686106803 Eh - Rel. G. = 3.300 kcal/mol - G(corr)(Eh) = 1.04258123

7 G = -3374.450503410496 Eh - Rel. G. = 3.415 kcal/mol - G(corr)(Eh) = 1.04340689

8 G = -3374.450474148718 Eh - Rel. G. = 3.433 kcal/mol - G(corr)(Eh) = 1.04432822

9 G = -3374.450336669217 Eh - Rel. G. = 3.519 kcal/mol - G(corr)(Eh) = 1.04545931

10 G = -3374.446869543723 Eh - Rel. G. = 5.695 kcal/mol - G(corr)(Eh) = 1.04345512

11 G = -3374.446675113837 Eh - Rel. G. = 5.817 kcal/mol - G(corr)(Eh) = 1.04385377

12 G = -3374.4465587005197 Eh - Rel. G. = 5.890 kcal/mol - G(corr)(Eh) = 1.04363868

13 G = -3374.445323600783 Eh - Rel. G. = 6.665 kcal/mol - G(corr)(Eh) = 1.04368935

14 G = -3374.445254293079 Eh - Rel. G. = 6.709 kcal/mol - G(corr)(Eh) = 1.04290003

15 G = -3374.444163224838 Eh - Rel. G. = 7.393 kcal/mol - G(corr)(Eh) = 1.04468163

16 G = -3374.441176545439 Eh - Rel. G. = 9.267 kcal/mol - G(corr)(Eh) = 1.04396647

17 G = -3374.440938815829 Eh - Rel. G. = 9.417 kcal/mol - G(corr)(Eh) = 1.04324621

18 G = -3374.4408701932316 Eh - Rel. G. = 9.460 kcal/mol - G(corr)(Eh) = 1.04296925

19 G = -3374.440214896888 Eh - Rel. G. = 9.871 kcal/mol - G(corr)(Eh) = 1.04192080

20 G = -3374.440196971806 Eh - Rel. G. = 9.882 kcal/mol - G(corr)(Eh) = 1.04286343

P7_S15_rep

1 G = -3374.4577148101357 Eh - Rel. G. = 0.000 kcal/mol - G(corr)(Eh) = 1.04438431

2 G = -3374.455702724285 Eh - Rel. G. = 1.263 kcal/mol - G(corr)(Eh) = 1.04499542

3 G = -3374.455631378184 Eh - Rel. G. = 1.307 kcal/mol - G(corr)(Eh) = 1.04397140

4 G = -3374.455483817172 Eh - Rel. G. = 1.400 kcal/mol - G(corr)(Eh) = 1.04430791

5 G = -3374.452456674449 Eh - Rel. G. = 3.300 kcal/mol - G(corr)(Eh) = 1.04381848

6 G = -3374.449704273257 Eh - Rel. G. = 5.027 kcal/mol - G(corr)(Eh) = 1.04503186

7 G = -3374.449465457055 Eh - Rel. G. = 5.177 kcal/mol - G(corr)(Eh) = 1.04476865

8 G = -3374.449103830605 Eh - Rel. G. = 5.403 kcal/mol - G(corr)(Eh) = 1.04456166

9 G = -3374.4489453660617 Eh - Rel. G. = 5.503 kcal/mol - G(corr)(Eh) = 1.04409531

10 G = -3374.448759215796 Eh - Rel. G. = 5.620 kcal/mol - G(corr)(Eh) = 1.04374389

11 G = -3374.4486466786584 Eh - Rel. G. = 5.690 kcal/mol - G(corr)(Eh) = 1.04327175

12 G = -3374.448561266067 Eh - Rel. G. = 5.744 kcal/mol - G(corr)(Eh) = 1.04378477

13 G = -3374.448220673896 Eh - Rel. G. = 5.958 kcal/mol - G(corr)(Eh) = 1.04305346

14 G = -3374.447895306222 Eh - Rel. G. = 6.162 kcal/mol - G(corr)(Eh) = 1.04386397

15 G = -3374.443713681677 Eh - Rel. G. = 8.786 kcal/mol - G(corr)(Eh) = 1.04258552

16 G = -3374.443457950211 Eh - Rel. G. = 8.946 kcal/mol - G(corr)(Eh) = 1.04275881

17 G = -3374.44342369924   Eh - Rel. G. = 8.968 kcal/mol - G(corr)(Eh) = 1.04304445

P7_S4_ent

1 G = -3530.3896437053354 Eh - Rel. G. = 0.000 kcal/mol - G(corr)(Eh) = 1.16616735

2 G = -3530.3855601260743 Eh - Rel. G. = 2.562 kcal/mol - G(corr)(Eh) = 1.16382266

3 G = -3530.3842030941782 Eh - Rel. G. = 3.414 kcal/mol - G(corr)(Eh) = 1.16476724

4 G = -3530.382481108541 Eh - Rel. G. = 4.495 kcal/mol - G(corr)(Eh) = 1.16504551

5 G = -3530.382070197979 Eh - Rel. G. = 4.752 kcal/mol - G(corr)(Eh) = 1.16574313

6 G = -3530.382004439334 Eh - Rel. G. = 4.794 kcal/mol - G(corr)(Eh) = 1.16611893

7 G = -3530.381313725572 Eh - Rel. G. = 5.227 kcal/mol - G(corr)(Eh) = 1.16456803

8 G = -3530.381012211448 Eh - Rel. G. = 5.416 kcal/mol - G(corr)(Eh) = 1.16425603

9 G = -3530.3799961390378 Eh - Rel. G. = 6.054 kcal/mol - G(corr)(Eh) = 1.16425999

10 G = -3530.379333646623 Eh - Rel. G. = 6.470 kcal/mol - G(corr)(Eh) = 1.16410725

11 G = -3530.379244251595 Eh - Rel. G. = 6.526 kcal/mol - G(corr)(Eh) = 1.16353615

12 G = -3530.378966813786 Eh - Rel. G. = 6.700 kcal/mol - G(corr)(Eh) = 1.16456637

13 G = -3530.375323693876 Eh - Rel. G. = 8.986 kcal/mol - G(corr)(Eh) = 1.16292169

14 G = -3530.374268507087 Eh - Rel. G. = 9.648 kcal/mol - G(corr)(Eh) = 1.16385038

P7_S4_rep

1 G = -3530.395807273837 Eh - Rel. G. = 0.000 kcal/mol - G(corr)(Eh) = 1.16585597

2 G = -3530.3954383115642 Eh - Rel. G. = 0.232 kcal/mol - G(corr)(Eh) = 1.16514553

3 G = -3530.395288891341 Eh - Rel. G. = 0.325 kcal/mol - G(corr)(Eh) = 1.16534425

4 G = -3530.393073254837 Eh - Rel. G. = 1.716 kcal/mol - G(corr)(Eh) = 1.16569794

5 G = -3530.392304911012 Eh - Rel. G. = 2.198 kcal/mol - G(corr)(Eh) = 1.16589346

6 G = -3530.390228499616 Eh - Rel. G. = 3.501 kcal/mol - G(corr)(Eh) = 1.16607700

7 G = -3530.38957565402   Eh - Rel. G. = 3.910 kcal/mol - G(corr)(Eh) = 1.16557741

8 G = -3530.388999498765 Eh - Rel. G. = 4.272 kcal/mol - G(corr)(Eh) = 1.16546241

9 G = -3530.388985141583 Eh - Rel. G. = 4.281 kcal/mol - G(corr)(Eh) = 1.16593456

10 G = -3530.388891589328 Eh - Rel. G. = 4.340 kcal/mol - G(corr)(Eh) = 1.16496848

11 G = -3530.388705213316 Eh - Rel. G. = 4.457 kcal/mol - G(corr)(Eh) = 1.16487754

12 G = -3530.388153491889 Eh - Rel. G. = 4.803 kcal/mol - G(corr)(Eh) = 1.16638518

13 G = -3530.387873611977 Eh - Rel. G. = 4.978 kcal/mol - G(corr)(Eh) = 1.16505173

14 G = -3530.387831953313 Eh - Rel. G. = 5.005 kcal/mol - G(corr)(Eh) = 1.16507399

15 G = -3530.3873341255457 Eh - Rel. G. = 5.317 kcal/mol - G(corr)(Eh) = 1.16551625

16 G = -3530.387222338673 Eh - Rel. G. = 5.387 kcal/mol - G(corr)(Eh) = 1.16547584

17 G = -3530.3867092677997 Eh - Rel. G. = 5.709 kcal/mol - G(corr)(Eh) = 1.16512486

18 G = -3530.385443352627 Eh - Rel. G. = 6.503 kcal/mol - G(corr)(Eh) = 1.16441692

TS-like ensembles

P7_S10_ent

1 SP_EE(Eh) = -3799.53350778, gcorr(Eh) = 1.19195630, G(Eh) = -3798.34155148, Rel. G(kcal/mol) = 0.00

2 SP_EE(Eh) = -3799.53334287, gcorr(Eh) = 1.19203484, G(Eh) = -3798.34130803, Rel. G(kcal/mol) = 0.15

3 SP_EE(Eh) = -3799.53343554, gcorr(Eh) = 1.19222940, G(Eh) = -3798.34120614, Rel. G(kcal/mol) = 0.22

4 SP_EE(Eh) = -3799.53180277, gcorr(Eh) = 1.19233828, G(Eh) = -3798.33946449, Rel. G(kcal/mol) = 1.31

P7_S10_rep

1 SP_EE(Eh) = -3799.54304433, gcorr(Eh) = 1.19298362, G(Eh) = -3798.35006071, Rel. G(kcal/mol) = 0.00

2 SP_EE(Eh) = -3799.54316441, gcorr(Eh) = 1.19338411, G(Eh) = -3798.34978030, Rel. G(kcal/mol) = 0.18

3 SP_EE(Eh) = -3799.54304827, gcorr(Eh) = 1.19362180, G(Eh) = -3798.34942647, Rel. G(kcal/mol) = 0.40

4 SP_EE(Eh) = -3799.54130504, gcorr(Eh) = 1.19245287, G(Eh) = -3798.34885217, Rel. G(kcal/mol) = 0.76

5 SP_EE(Eh) = -3799.54064825, gcorr(Eh) = 1.19259269, G(Eh) = -3798.34805556, Rel. G(kcal/mol) = 1.26

P7_S15_ent

1 SP_EE(Eh) = -3375.49833684, gcorr(Eh) = 1.04676664, G(Eh) = -3374.45157020, Rel. G(kcal/mol) = 0.00

2 SP_EE(Eh) = -3375.49326734, gcorr(Eh) = 1.04258123, G(Eh) = -3374.45068611, Rel. G(kcal/mol) = 0.55

3 SP_EE(Eh) = -3375.49391030, gcorr(Eh) = 1.04340689, G(Eh) = -3374.45050341, Rel. G(kcal/mol) = 0.67

P7_S15_rep

1 SP_EE(Eh) = -3375.50209912, gcorr(Eh) = 1.04438431, G(Eh) = -3374.45771481, Rel. G(kcal/mol) = 0.00

2 SP_EE(Eh) = -3375.50069814, gcorr(Eh) = 1.04499542, G(Eh) = -3374.45570272, Rel. G(kcal/mol) = 1.26

3 SP_EE(Eh) = -3375.49960278, gcorr(Eh) = 1.04397140, G(Eh) = -3374.45563138, Rel. G(kcal/mol) = 1.31

4 SP_EE(Eh) = -3375.49979173, gcorr(Eh) = 1.04430791, G(Eh) = -3374.45548382, Rel. G(kcal/mol) = 1.40

P7_S4_ent

1 SP_EE(Eh) = -3531.54938279, gcorr(Eh) = 1.16382266, G(Eh) = -3530.38556013, Rel. G(kcal/mol) = 0.00

2 SP_EE(Eh) = -3531.54897033, gcorr(Eh) = 1.16476724, G(Eh) = -3530.38420309, Rel. G(kcal/mol) = 0.85

3 SP_EE(Eh) = -3531.54752662, gcorr(Eh) = 1.16504551, G(Eh) = -3530.38248111, Rel. G(kcal/mol) = 1.93

P7_S4_rep

1 SP_EE(Eh) = -3531.56166324, gcorr(Eh) = 1.16585597, G(Eh) = -3530.39580727, Rel. G(kcal/mol) = 0.00

2 SP_EE(Eh) = -3531.56058384, gcorr(Eh) = 1.16514553, G(Eh) = -3530.39543831, Rel. G(kcal/mol) = 0.23

3 SP_EE(Eh) = -3531.56063314, gcorr(Eh) = 1.16534425, G(Eh) = -3530.39528889, Rel. G(kcal/mol) = 0.33

4 SP_EE(Eh) = -3531.55877119, gcorr(Eh) = 1.16569794, G(Eh) = -3530.39307325, Rel. G(kcal/mol) = 1.72

3. Additional figures

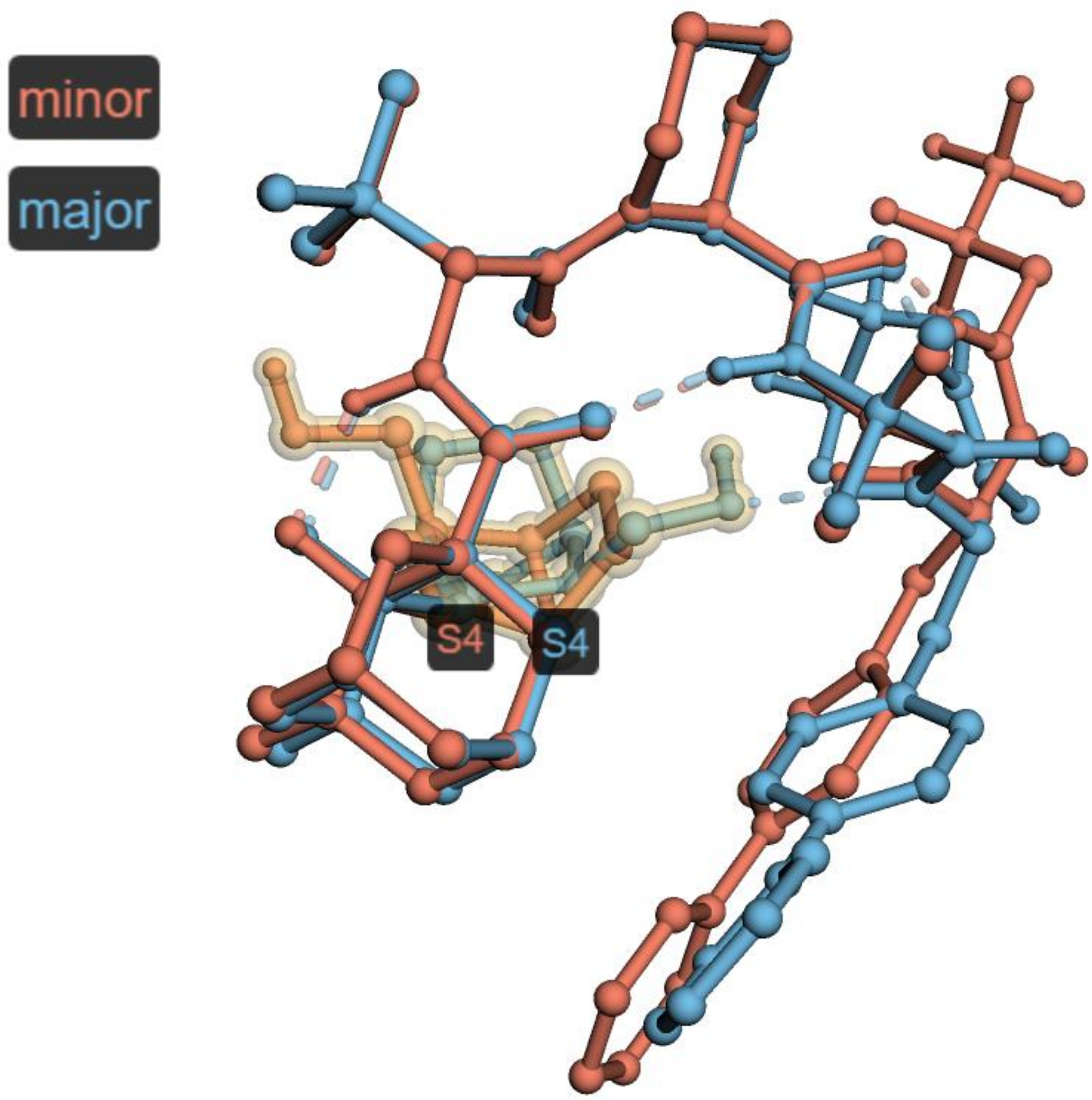

Figure S7. Best TS-like ground state conformers for the major and minor diastereomers of the **P7/S4** oxoammonium alkoxide complex. Both complexes feature deviations from the “main” binding mode observed throughout the optimization due to the conformational restriction of the cyclic substrate. The major complex features one less hydrogen bond than the usually observed “main” mode, and the minor complex features two less. This is consistent with the observed high selectivity of the reaction (96% ee)

4. References

(1) Kishioka, S.-y.; Yamada, A. Kinetic study of the catalytic oxidation of benzyl alcohols by phthalimide-N-oxyl radical electrogenerated in acetonitrile using rotating disk electrode voltammetry. *J. Electroanal. Chem.* **2005**, *578* (1), 71-77. DOI: https://doi.org/10.1016/j.jelechem.2004.12.021.

(2) Ahn, S. D.; Fisher, A. C.; Buchard, A.; Bull, S. D.; Bond, A. M.; Marken, F. Hydrodynamic Rocking Disc Electrode Study of the TEMPO-mediated Catalytic Oxidation of Primary Alcohols. *Electroanalysis* **2016**, *28* (9), 2093-2103. DOI: https://doi.org/10.1002/elan.201600141.

(3) Haberland, D.; Landsberg, R. Zur Anwendung der rotierenden Scheibenelektrode beim Studium nachgelagerter chemischer Reaktionen. *Berichte der Bunsengesellschaft für physikalische Chemie* **1966**, *70* (7), 724-727. DOI: https://doi.org/10.1002/bbpc.19660700708 (acccessed 2025/08/13).

(4) *Avogadro*; https://avogadro.cc/ (accessed.

(5) Pracht, P.; Bohle, F.; Grimme, S. Automated exploration of the low-energy chemical space with fast quantum chemical methods. *Physical Chemistry Chemical Physics* **2020**, *22* (14), 7169-7192, 10.1039/C9CP06869D. DOI: 10.1039/C9CP06869D.

(6) Bannwarth, C.; Ehlert, S.; Grimme, S. GFN2-xTB—An Accurate and Broadly Parametrized Self-Consistent Tight-Binding Quantum Chemical Method with Multipole Electrostatics and Density-Dependent Dispersion Contributions. *J. Chem. Theory Comput.* **2019**, *15* (3), 1652-1671. DOI: 10.1021/acs.jctc.8b01176.

(7) Spicher, S.; Grimme, S. Robust Atomistic Modeling of Materials, Organometallic, and Biochemical Systems. *Angew. Chem. Int. Ed.* **2020**, *59* (36), 15665-15673. DOI: https://doi.org/10.1002/anie.202004239 (acccessed 2023/09/18).

(8) Ehlert, S.; Stahn, M.; Spicher, S.; Grimme, S. Robust and Efficient Implicit Solvation Model for Fast Semiempirical Methods. *J. Chem. Theory Comput.* **2021**, *17* (7), 4250-4261. DOI: 10.1021/acs.jctc.1c00471.

(9) Tampellini, N. *FIRECODE - Filtering Refiner and Embedder for Conformationally Dense Ensembles (version 1.1.0)*. 2020-2025. https://github.com/ntampellini/FIRECODE (accessed 2025.

(10) Furness, J. W.; Kaplan, A. D.; Ning, J.; Perdew, J. P.; Sun, J. Accurate and Numerically Efficient r2SCAN Meta-Generalized Gradient Approximation. *The Journal of Physical Chemistry Letters* **2020**, *11* (19), 8208-8215. DOI: 10.1021/acs.jpclett.0c02405.

(11) Furness, J. W.; Kaplan, A. D.; Ning, J.; Perdew, J. P.; Sun, J. Correction to "Accurate and Numerically Efficient r2SCAN Meta-Generalized Gradient Approximation". *The Journal of Physical Chemistry Letters* **2020**, *11* (21), 9248-9248. DOI: 10.1021/acs.jpclett.0c03077.

(12) Grimme, S.; Hansen, A.; Ehlert, S.; Mewes, J.-M. r2SCAN-3c: A "Swiss army knife" composite electronic-structure method. *J. Chem. Phys.* **2021**, *154* (6), 064103. DOI: 10.1063/5.0040021 (acccessed 7/15/2023).

(13) Cossi, M.; Rega, N.; Scalmani, G.; Barone, V. Energies, structures, and electronic properties of molecules in solution with the C-PCM solvation model. *J. Comput. Chem.* **2003**, *24* (6), 669-681, https://doi.org/10.1002/jcc.10189. DOI: https://doi.org/10.1002/jcc.10189 (acccessed 2023/07/15).

(14) Neese, F. Software update: The ORCA program system—Version 5.0. *WIREs Computational Molecular Science* **2022**, *12* (5), e1606, https://doi.org/10.1002/wcms.1606. DOI: https://doi.org/10.1002/wcms.1606 (acccessed 2023/07/15).

(15) Neese, F.; Wennmohs, F.; Becker, U.; Riplinger, C. The ORCA quantum chemistry program package. *J. Chem. Phys.* **2020**, *152* (22), 224108. DOI: 10.1063/5.0004608 (acccessed 7/15/2023).

(16) Mardirossian, N.; Head-Gordon, M. ωB97M-V: A combinatorially optimized, range-separated hybrid, meta-GGA density functional with VV10 nonlocal correlation. *J. Chem. Phys.* **2016**, *144* (21), 214110. DOI: 10.1063/1.4952647 (acccessed 10/18/2023).

(17) Weigend, F.; Ahlrichs, R. Balanced basis sets of split valence, triple zeta valence and quadruple zeta valence quality for H to Rn: Design and assessment of accuracy. *Physical Chemistry Chemical Physics* **2005**, *7* (18), 3297-3305, 10.1039/B508541A. DOI: 10.1039/B508541A.
